# Genetics of long‐distance runners and road cyclists—A systematic review with meta‐analysis

**DOI:** 10.1111/sms.14212

**Published:** 2022-07-26

**Authors:** Magdalena Johanna Konopka, Jorn Carlos Maria Leonardus van den Bunder, Gerard Rietjens, Billy Sperlich, Maurice Petrus Zeegers

**Affiliations:** ^1^ Care and Public Health Research Institute (CAPHRI) Maastricht University Maastricht The Netherlands; ^2^ Department of Epidemiology Maastricht University Medical Centre Maastricht The Netherlands; ^3^ Department of Human Physiology and Sports Medicine Vrije Universiteit Brussel Brussels Belgium; ^4^ Integrative & Experimental Exercise Science & Training, Institute of Sport Science University of Würzburg Würzburg Germany; ^5^ School of Nutrition and Translational Research in Metabolism (NUTRIM) Maastricht University Maastricht The Netherlands

**Keywords:** DNA, endurance athletes, genetic variant, polymorphism, predisposition, sport genetics

## Abstract

The aim of this systematic review and meta‐analysis was to identify the genetic variants of (inter)national competing long‐distance runners and road cyclists compared with controls. The Medline and Embase databases were searched until 15 November 2021. Eligible articles included genetic epidemiological studies published in English. A homogenous group of endurance athletes competing at (inter)national level and sedentary controls were included. Pooled odds ratios based on the genotype frequency with corresponding 95% confidence intervals (95%CI) were calculated using random effects models. Heterogeneity was addressed by Q‐statistics, and *I* ^2^. Sources of heterogeneity were examined by meta‐regression and risk of bias was assessed with the Clark Baudouin scale. This systematic review comprised of 43 studies including a total of 3938 athletes and 10 752 controls in the pooled analysis. Of the 42 identified genetic variants, 13 were investigated in independent studies. Significant associations were found for five polymorphisms. Pooled odds ratio [95%CI] favoring athletes compared with controls was 1.42 [1.12–1.81] for *ACE* II (I/D), 1.66 [1.26–2.19] for *ACTN3* TT (rs1815739), 1.75 [1.34–2.29] for *PPARGC1A* GG (rs8192678), 2.23 [1.42–3.51] for *AMPD1* CC (rs17602729), and 2.85 [1.27–6.39] for *HFE* GG + CG (rs1799945). Risk of bias was low in 25 (58%) and unclear in 18 (42%) articles. Heterogeneity of the results was low (0%–20%) except for *HFE* (71%), *GNB3* (80%), and *NOS3* (76%). (Inter)national competing runners and cyclists have a higher probability to carry specific genetic variants compared with controls. This study confirms that (inter)national competing endurance athletes constitute a unique genetic make‐up, which likely contributes to their performance level.

## 1 INTRODUCTION

Endurance performance is a complex trait and the necessary phenotypes (e.g., maximal oxygen uptake) for success are influenced by numerous internal and external factors such as genetic composition,^1^ training process,^2^ psychological factors,^3^, ^4^ or nutrition.^5^ It is well known that purposeful (exercise) behavior and a promising degree of talent are necessary for success. Therefore, the theoretical probability of becoming a successful athlete is likely to increase proportionately with a higher number of advantageous alleles.^6^, ^7^ For instance, athletes with a certain genetic make‐up are more prone to achieve favorable physiological adaptation for high levels of performance compared with athletes with a less advantageous genetic profile.

One of the first sport genetic publications estimated the heritability of maximal oxygen uptake in response to training approximately at 47%^8^ and a genome wide linkage scan of female twins reported a heritability of 66% for athletic performance.^9^ Later on, a comprehensive review identified 155 genetic markers linked to athletic status.^10^ The angiotensin I converting enzyme insertion/deletion variant (*ACE* I/D) is one of the most frequently studied variation in sport genetics, and the insertion allele has been linked to endurance performance, while the deletion allele has been linked to muscular power.^11^, ^12^ Traditionally, polymorphism investigations in sport science have focused on the two opposite sides of the neuromuscular spectrum, that is, power vs. endurance sports.^13^, ^14^ Unfortunately, this distinct classification of “power” vs. “endurance” results in heterogeneous study populations,^15^, ^16^, ^17^ since, for example, badminton, soccer, or rowing altogether have been classified as “endurance” sport.^18^ This somewhat “rough” clustering of sport disciplines into one trait is problematic because each discipline encompasses unique phenotypic characteristics for success.^19^ Therefore, it seems reasonable to focus on more homogenous endurance disciplines, for example, runners and cyclists since these disciplines are predominantly leg‐dominated sports with similar and distinct physiological surrogates (e.g., peak oxygen uptake) explaining the level of performance to a high degree.^20^, ^21^, ^22^

Moreover, within bio‐medical research the term “athlete” is used inconsistently leading to frequent misinterpretations of the performance level.^23^ The literature contains a variety of definitions including “highly trained,” “elite,” and “professional” altogether attempting to describe an exclusive population with extraordinary (genetic) ability to adapt. However, these definitions are rather subjective than objective^24^ and become problematic especially when comparing data from different studies dealing with different performance levels. For several reasons, it may be more appropriate to simply classify performance level into “national” or “international” competing athletes: (i) there are documented norms for competing on (inter)national level and (ii) classification into (inter)national competing athletes allows for retrieval of a larger sample size to evaluate genetic associations.^23^

To the best of our knowledge, no comprehensive meta‐analysis has been conducted to identify the interplay between genetic variants and performance level in a homogeneous group of endurance athletes (i.e., runners and cyclists). By combining the results of multiple scientific studies in the form of a meta‐analysis, the problem of small sample sizes can be partially overcome and at the same time provides a more valid pooled estimate.^25^ Therefore, the aim of this study was to systematically identify genetic variants of (inter)national competing runners and cyclists compared to sedentary controls.

## 2 METHODS

### 2.1 Eligibility criteria

This meta‐analysis was conducted according to the 2020 PRISMA guidelines^26^ (see Appendix S1 for the PRISMA checklist) and eligible reports composed of peer reviewed genetic association studies investigating (inter)national competing runners and cyclists published in English. To ensure a certain degree of homogeneity, we included only long‐distance runners (main discipline ≥5000 m) as well as road cyclists (i.e., no mountain bikers or cyclocross racers) competing on (inter)national level and sedentary controls.^23^ A non‐athletic population was chosen as reference, because the pool of this group is relatively extensive, so, the chances of this group for possessing alleles connected to (inter)national performance are low. National performance level was defined as participating in national championship events and international performance level as participating in world championship events. To further classify runners, marathon performance times under 2 h 20 min (males) and 2 h 45 min (females) were considered international performance level.^27^, ^28^, ^29^, ^30^, ^31^, ^32^ For cyclists, a maximal oxygen uptake of ≥ 71 ml/kg/min confirmed high level of competition.^22^, ^33^, ^34^ Case studies and conference abstracts were excluded. Articles were also excluded when no correct rs‐number or no information about genotype frequency was reported or when no nuclear genome data was analyzed. Candidate gene studies analyzing more than 30 different genetic variants were excluded for efficiency reasons but will be addressed in the discussion section. When articles involved identical participants the most recent article was included for analysis. An exception was made when the older study reported stratified results for discipline (runners vs. cyclists), performance level (national vs. international), or sex (male vs. female). In case studies employed, partly same athletic cohorts only new sub‐cohorts were included.^35^

### 2.2 Study selection and data extraction

Medline and Embase databases were searched on July 1, 2020, and updated on November 15, 2021. All relevant studies and reviews related to the topic were checked for cross references. The search strategy for both databases consisted of three online searches. The search terms and the full search strategies are summarized in ESM 2, 3, and 4. No filters were applied during the search. Identified studies from databases were extracted to Endnote (Clarivate Analytics) and automatically screened for duplicates. Title and abstract of the retrieved reports were initially read by one reviewer (MK). Two independent reviewers (MK and JB) then screened the full texts for eligibility. Disagreement between MK and JB was solved by discussion. Data collection was performed independently by the two reviewers using Excel. The following items were extracted: first author's name, publication year, journal, country of origin, ethnicity and sex of participants, number and performance level of athletes (runners and cyclists), number of controls, rs‐number, and genotype frequencies. The authors were contacted in case the full text was not available or relevant information for data extraction was missing or unclear.

### 2.3 Risk of bias assessment

The risk of bias within each study was assessed independently by the two reviewers using the Clark Baudouin scale, a 10‐point scoring system for the quality of genetic association studies.^36^ This scale assesses size of cases and controls, Hardy Weinberg equilibrium of controls, adequate definition of cases, primer sequence, genotyping accuracy and reproducibility, statistical power, corrections for multiplicity, and replication of results. The scores range from 0 (worst) to 10 (best), and overall scoring is based on quality (low ≤ 4, moderate 5–7, and high ≥ 8). Risk of bias was rated as “high” for low quality studies, as “unclear” for moderate quality and as “low” for high quality studies. The Robvis tool was used to visualize risk‐of‐bias.^37^

### 2.4 Synthesis of results

Odds ratios between (inter)national competing runners and cyclists and controls were calculated for genotype status and genetic variant assuming a recessive inheritance model. A dominant inheritance model was assumed when zero counts were reported for the effect allele. Eligible for the synthesis of results were at least two independent studies analyzing identical variants. All statistical analyses were performed with R (version 4.0.3). Pooling of effect sizes (odds ratio) was performed based on raw genotype data using the meta package/metabin function. The random effects model for pooling of effect size was applied because between‐study heterogeneity was expected.^38^ The exact Mantel–Haenszel method for statistical pooling was used and the Wald test for the confidence interval of the summary effect. Furthermore, 95% prediction intervals were calculated.^39^

### 2.5 Heterogeneity and sensitivity analyses

Heterogeneity was analyzed by Q‐statistics based on the Mantel–Haenszel estimator and tau^2^ (DerSimonian and Laird).^40^ *I* ^2^ represented inconsistency and was categorized as low (<25%), moderate (25%–75%), or high (>75%). Heterogeneity, outliers, and influential cases were inspected with Baujat and Gosh plots^41^, ^42^ and the leave‐one‐out method. In case the meta‐analysis included ≥5 reports, we examined publication bias with funnel plot asymmetry. In case the meta‐analysis included ≥10 reports,^43^ publication bias was statistically tested with linear regression analysis.^44^ In addition, we employed random effects meta‐regressions to examine statistical heterogeneity in case ≥10 articles were included in the pooled analysis.^45^ When significant, we performed subgroup analysis using a random effects model comparing “discipline,” “ethnicity,” “performance level,” and “sex.” International level of competition was expected to have a greater effect size compared with national level of competition. No expectations were set for sex, discipline, or ethnicity. In addition, we performed several sensitivity analyses: (i) a fixed effect model, (ii) a dominant inheritance model, (iii) Hartung–Knapp adjustment,^46^ (iv) exclusion of studies violating the Hardy Weinberg equilibrium,^47^ and (v) exclusion of studies with high risk of bias. Significance for all analyses was *p* < 0.05.

### 2.6 Certainty of evidence

Certainty of the evidence was assessed using the GRADE framework categorizing the evidence as high, moderate, low, or very low.^48^ Certainty of evidence was graded by the two reviewers independently, and disagreement was solved by discussion.

## 3 RESULTS

### 3.1 Study selection

**FIGURE 1:**
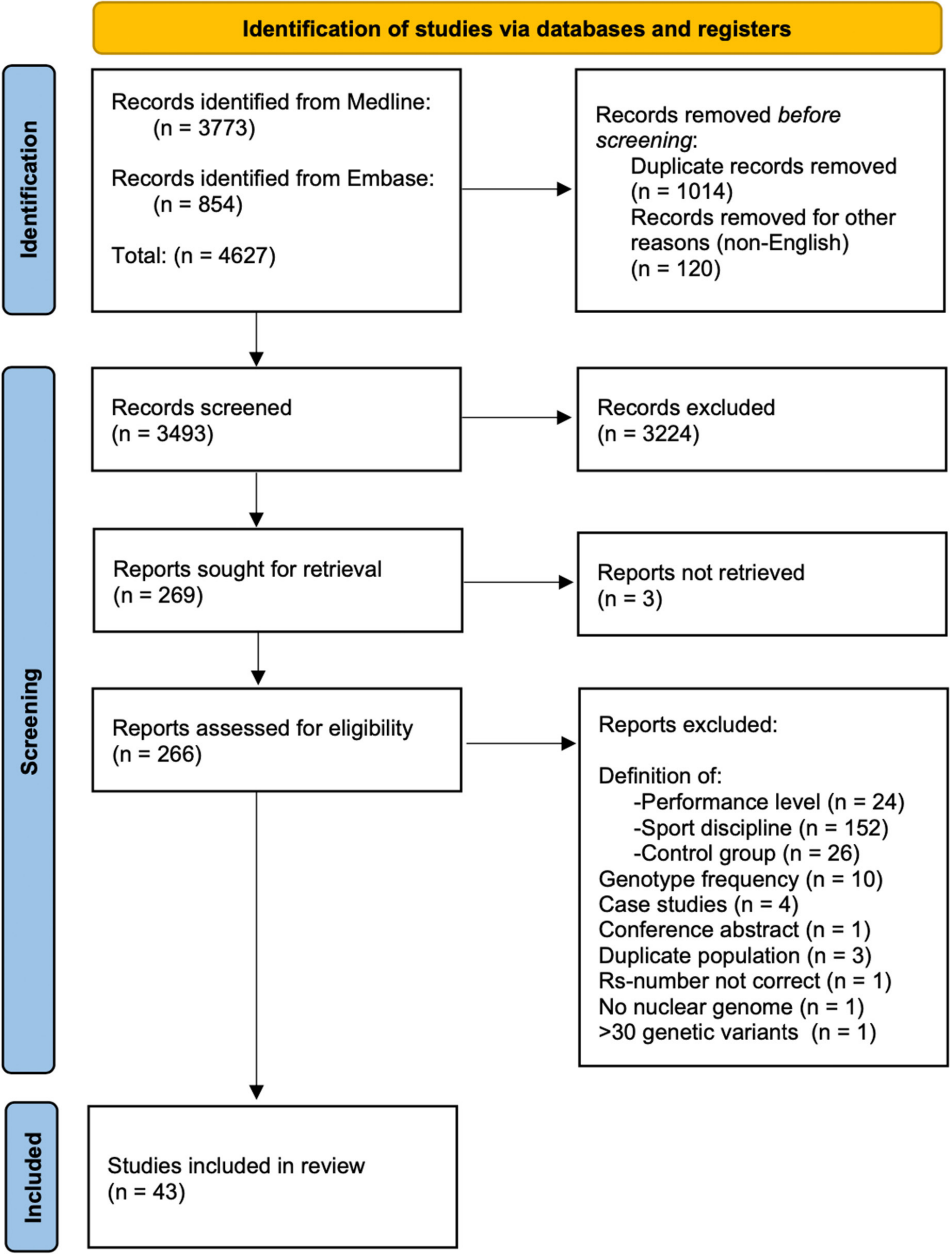
PRISMA flow chart for the study selection

The literature search identified 4627 records. Of the initial dataset, 1014 duplicates were removed as well as 120 non‐English studies. Consequently, 3493 articles were screened based on title and abstract thereby excluding another 3224 records. Next, 269 articles were read for detailed evaluation of which three articles were not retrievable even after contacting the author(s).^49^, ^50^, ^51^ Of the remaining 266 studies, 43 were eligible for inclusion. The journals of all included articles are summarized in ESM 5. Figure 1 depicts the flow of study selection. The reasons for study exclusion were other definitions of the performance level (*n* = 24), other definitions of the sport disciplines (*n* = 152) or control group (*n* = 26), unclear genotype frequency (*n* = 10), case studies (*n* = 4),^52^, ^53^, ^54^, ^55^ conference abstracts (*n* = 1), duplicate study populations (*n* = 3),^32^, ^56^, ^57^ incorrect rs‐number (*n* = 1),^58^ no nuclear genome (*n* = 1),^59^ and >30 genetic variants analyzed (*n* = 1).^60^ The reasons for exclusion are summarized in ESM 6.

### 3.2 Study characteristics, risk of bias, and results of individual studies

ESM 7 displays the key characteristics of all included studies. ESM 8 presents the observed genotype and allele frequencies as well as the estimated population frequencies. Of the 43 articles, 25 (58%) were rated as low and 18 (42%) were rated as unclear risk of bias. Detailed information about the risk of bias assessment is summarized in ESM 9. Overall, we identified 42 genetic variants. Of those, 29 variants were investigated by single studies included in this meta‐analysis (ESM 10). Therefore, these 29 polymorphisms could not statistically be pooled and 12 of the 29 variants showed a significant association with runners and cyclists competing at (inter)national level compared with controls.

### 3.3 Results of synthesis

Thirteen genetic variants were examined in at least two independent studies, yielding 3938 (inter)national runners and cyclists and 10 752 controls in total (Table 1). Of the 13 variants, five showed significant associations with runners and cyclists compared with controls. The pooled odds ratio [95%CI] favoring (inter)national competing runners and cyclists was 1.42 [1.12–1.81] for *ACE* I/D (II vs. ID+DD), 1.66 [1.26–2.19] for alpha‐actinin 3 (*ACTN3*) rs1815739 (TT vs. CT + CC), 1.75 [1.34–2.29] for peroxisome proliferator‐activated receptor gamma coactivator 1‐alpha (*PPARGC1A*) polymorphism rs8192678 (GG vs. AG + AA), 2.23 [1.42–3.51] for adenosine monophosphate deaminase 1 (*AMPD1*) rs17602729 (CC vs. CT + TT), and 2.85 [1.27–6.39] for homeostatic iron regulator (*HFE*) rs1799945 (GG + CG vs. CC). The remaining eight variants did not show a significant result in the main analysis: myostatin (*MSTN*, rs1805086), bradykinin receptor B2 (*BDKRB*, −9/+9), interleukin‐6 (*IL6*, rs1800795), adrenoceptor beta 2 (*ADRB2*, rs1042713 and rs1042714), creatine kinase M‐type (*CKMM*, rs8111989), G protein subunit beta 3 (*GNB3*, rs5443), and nitric oxide synthase 3 (*NOS3*, rs2070744). Heterogeneity of the pooled results was low (0%–20%) except for *HFE* (71%), *GNB3* (80%) and *NOS3* (76%).

**TABLE 1:** Summary of findings table including the pooled results of the 13 genetic variants investigated in at least two independent studies

Genetic variations associated with (inter)national competing runners and cyclists compared to controls. Population: healthy runners and cyclists competing on national or international level, healthy non‐athletic controls. Setting: community. Intervention: effect allele. Comparison: non‐effect allele.						
Rs‐number/ marker	Genotypes	Gene (dbSNP) (Reported in article)	Number of participants Athletes/controls (n studies)	Pooled Odds Ratio [95% confidence interval] [Prediction interval]	Certainty of evidence (GRADE)	Comments
*CE* I/D^a^ ^,^ ^61^, ^62^, ^63^, ^64^, ^65^, ^66^, ^67^, ^68^, ^69^, ^70^, ^71^, ^72^, ^73^, ^83^	**II vs. ID + DD**	***ACE***	**782/4637 (14)**	**1.42 [1.12–1.81]** **[0.85–2.39]**	**High**	**Risk of bias: low‐unclear** **Inconsistency (*I*** ^**2**^ **): low (20%)** **Imprecision: low** **Indirectness: low** **Publication bias: low**
rs1815739^66^, ^67^, ^68^, ^69^, ^73^, ^74^, ^75^, ^76^, ^77^, ^78^	**TT vs. CT + CC**	***ACTN3***	**557/1085 (9)**	**1.66 [1.26–2.19]** **[0.96–2.88]**	**High**	**Risk of bias: low‐unclear** **Inconsistency (*I*** ^**2**^ **): low (19%)** **Imprecision: low** **Indirectness: low** **Publication bias: low**
rs8192678^28^, ^67^, ^68^, ^79^, ^80^	**GG vs.** **AG + AA**	***PPARGC1A***	**359/1292 (5)**	**1.75 [1.34–2.29]** **[1.13–2.71]**	**High**	**Risk of bias: low‐unclear** **Inconsistency (*I*** ^**2**^ **): low (0%)** **Imprecision: low** **Indirectness: low‐unclear** **Publication bias: low**
rs17602729^28^, ^67^, ^68^	**CC vs. CT + TT**	***AMPD1***	**271/368 (3)**	**2.23 [1.42–3.51]** **[0.12–41.61]**	**Moderate**	**Risk of bias: unclear** **Inconsistency (*I*** ^**2**^ **): low (0%)** **Imprecision: unclear** **Indirectness: low** **Publication bias: high**
rs1799945^28^, ^67^ DM	**GG + CG vs. CC**	***HFE***	**169/245 (2)**	**2.85 [1.27–6.39]**	**Moderate**	**Risk of bias: unclear** **Inconsistency (*I*** ^**2**^ **): moderate (71%)** **Imprecision: high** **Indirectness: low** **Publication bias: high**
rs1805086^67^, ^68^, ^69^, ^81^ DM, HAKN	GG + AG vs. AA	*MSTN* (GDF8)	361/464 (4)	1.40 [0.90–2.19] [0.53–3.74]	Moderate	Risk of bias: unclear Inconsistency (*I* ^2^): low, (0%) Imprecision: high Indirectness: low Publication bias: unclear
*BDKRB* −9/+9^30^, ^82^, ^83^	+9 + 9 vs. +9–9/−9–9	*BDKRB2*	211/1046 (3)	1.44 [0.98–2.11] [0.12–17.07]	Moderate	Risk of bias: low‐unclear Inconsistency (*I* ^2^): low, (0%) Imprecision: high Indirectness: low Publication bias: high
rs1800795^31^, ^84^, ^85^	CC vs. CG + GG	*IL6*	237/369 (3)	1.23 [0.62–2.44] [0.01–105.40]	Moderate	Risk of bias: low‐unclear Inconsistency (*I* ^2^): low, (0%) Imprecision: high Indirectness: low Publication bias: high
rs1042713^83^, ^86^	GG vs. AG + AA	*ADRB2*	223/222 (2)	0.99 [0.67–1.46]	Moderate	Risk of bias: unclear Inconsistency (*I* ^2^): low, (0%) Imprecision: high Indirectness: low Publication bias: high
rs1042714^83^, ^86^	CC vs. GC + GG	*ADRB2*	223/222 (2)	1.29 [0.86–1.96]	Moderate	Risk of bias: unclear Inconsistency (*I* ^2^): low, (0%) Imprecision: high Indirectness: low Publication bias: high
rs8111989^a^ ^,^ 67, 68 HAKN	TT vs. TC + CC	*CKMM*	148/246 (2)	1.50 [0.98–2.30]	Moderate	Risk of bias: unclear Inconsistency (*I* ^2^): low, (0%) Imprecision: high Indirectness: low Publication bias: high
rs5443^87^, ^88^	TT vs. CT + CC	*GNB3*	174/334 (2)	1.32 [0.36–4.86]	Low	Risk of bias: low Inconsistency (I^2^): high, (80%) Imprecision: high Indirectness: low Publication bias: high
rs2070744^83^, ^89^	TT vs. CT + CC	*NOS3*	223/222 (2)	1.46 [0.65–3.24]	Low	Risk of bias: low‐unclear Inconsistency (I^2^): high, (76%) Imprecision: high Indirectness: low Publication bias: high

*Note*: Significant results are displayed in bold.

Prediction intervals have been calculated for *n* > 2 studies.

**FIGURE 2:**
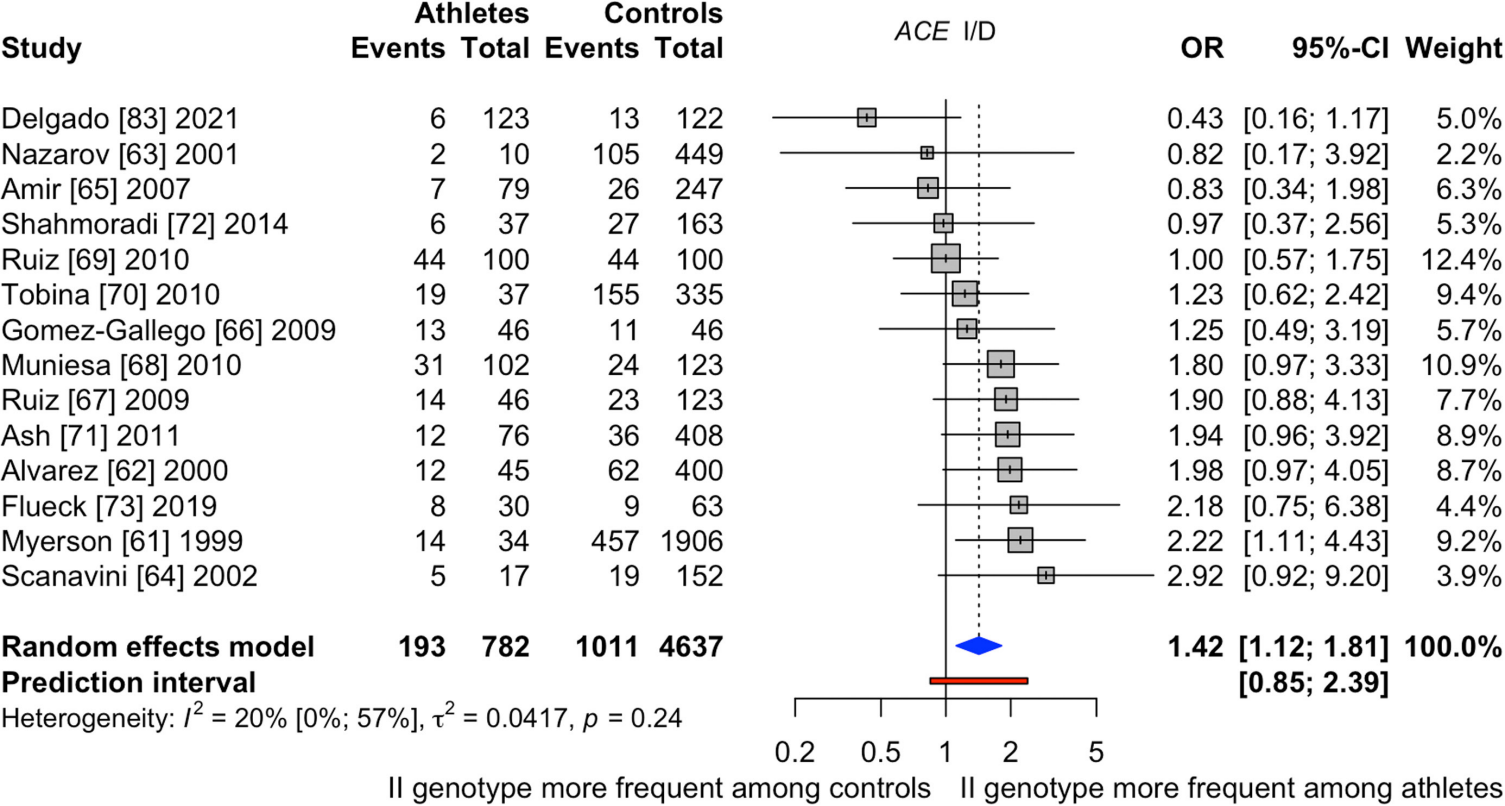
Results of the meta‐analysis investigating *ACE* I/D when comparing (inter)national runners and cyclists to controls; Events = II genotype

Exact values on inconsistency and imprecision for significant results are shown in Figure 2–6.

Study characteristics, risk of bias assessment, and funnel plots of significant results are presented in the ESM.

Abbreviations: ADM, dominant inheritance model assumed; HAKN, Significant when applying the Hartung–Knapp adjustment.

^a^ Rs‐number not mentioned in original article.

#### 3.3.1 ACE

The following figures illustrate the forest plots of the significant genetic variants associated with (inter)national runners and cyclists compared with controls. Figure 2 displays 14 studies analyzing *ACE* I/D with in total 782 (inter)national competing runners and cyclists and 4637 controls.^61^, ^62^, ^63^, ^64^, ^65^, ^66^, ^67^, ^68^, ^69^, ^70^, ^71^, ^72^, ^73^, ^83^ Seven publications were rated as low^61^, ^62^, ^65^, ^66^, ^71^, ^72^, ^73^ and seven as unclear^63^, ^64^, ^67^, ^68^, ^69^, ^70^, ^83^ risk of bias. ESM 11 and 12 present the *ACE*‐specific study characteristics and the risk of bias assessment. The reports mainly involved Caucasian participants (*n* = 12). One study comprised African^71^ and one Asian^70^ participants. Seven articles analyzed international,^61^, ^64^, ^66^, ^68^, ^71^, ^73^, ^83^ two national,^63^, ^65^ and the remaining studies investigated both national and international athletes together.^62^, ^67^, ^69^, ^72^ Finally, five publications investigated runners,^61^, ^63^, ^65^, ^70^, ^71^ two examined cyclists,^66^, ^72^ and the remaining seven publications analyzed both disciplines of which three analyzed runners and cyclists apart.^62^, ^64^, ^68^

In general, (inter)national competing runners and cyclists showed a significantly higher prevalence of the II genotype (II vs. ID + DD) compared with controls (pooled odds ratio [95%CI]: 1.42 [1.12–1.81]). The II genotype frequency was 24.7% in athletes and 21.8% in controls. The between‐study heterogeneity variance was estimated with *T* ^2^ = 0.04 and an *I* ^2^ [95%CI] value of 20.0% [0.0%–57.2%]. Meta‐regression results for sex (*p* = 0.23), discipline (*p* = 0.79), ethnicity (*p* = 0.74), and performance level (*p* = 0.26) were not significant. Finally, reporting biases were not detected, funnel plot (ESM 13) asymmetry was not significant (*p* = 0.24), and certainty of evidence was rated as high (Table 1).

#### 3.3.2 ACTN3

**FIGURE 3:**
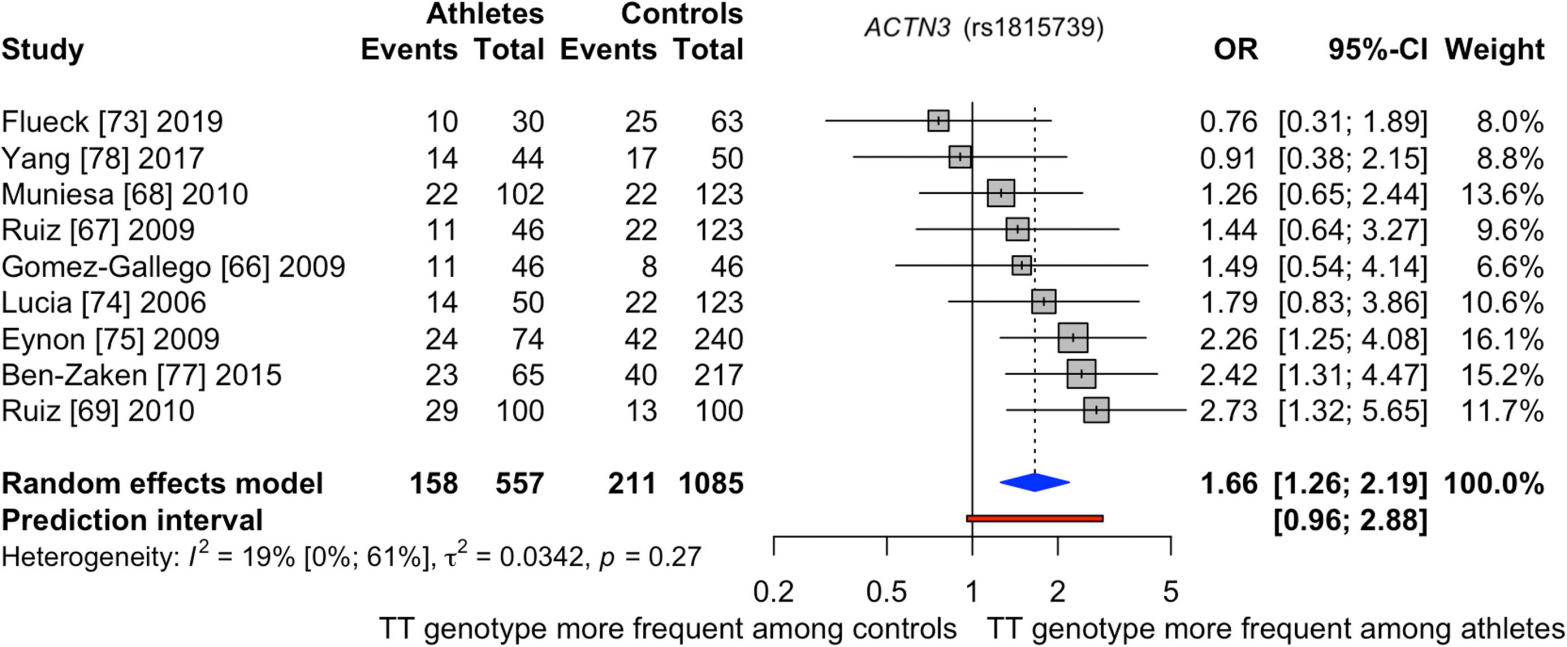
Results of the meta‐analysis investigating *ACTN3* (rs1815739) when comparing (inter)national runners and cyclists to controls; Events = TT genotype

Figure 3 illustrates the pooled results for *ACTN3* in which 557 (inter)national competing runners and cyclists as well as 1085 controls were analyzed (see ESM 14 for the study characteristics analyzing *ACTN3*). In total, nine studies were included in the meta‐analysis of *ACTN3*.^66^, ^67^, ^68^, ^69^, ^73^, ^74^, ^75^, ^77^, ^78^ Four reports showed low^73^, ^74^, ^75^, ^78^ and five showed unclear^66^, ^67^, ^68^, ^69^, ^77^ risk of bias (ESM 15). The study populations consisted mainly of Caucasian origin. Only one study involved Chinese participants.^78^ Three articles investigated international,^66^, ^68^, ^73^ one national,^74^ and the remaining five^67^, ^69^, ^75^, ^77^, ^78^ employed runners and cyclists competing at national and international level as one group. In addition, three publications examined runners,^75^, ^77^, ^78^ two cyclists,^66^, ^74^ and four investigated runners and cyclists together.^67^, ^68^, ^69^, ^73^

(Inter)national competing runners and cyclists showed 1.66 times the odds of possessing the TT genotype (TT vs. CT + CC) compared with controls (pooled odds ratio [95%CI]: 1.66 [1.26–2.19]). The frequency of the TT genotype was 28.4% in the athlete and 19.5% in the control group. The between‐study heterogeneity variance was estimated with *T* ^2^ = 0.03 and *I* ^2^ at 19.1% [0.0%–60.8%]. Meta‐regression results regarding sex (*p* = 0.86), discipline (*p* = 0.77), ethnicity (*p* = 0.16), and performance level (*p* = 0.17) were not significant. Noteworthy, two publications^67^, ^68^ might have employed the same control group (*N* = 123) but different athletes. Excluding one or both studies did not change the present finding (*p* < 0.01). The control group of four reports^66^, ^73^, ^75^, ^77^ deviated from Hardy Weinberg equilibrium and excluding those studies also did not change the main result (*p* < 0.01). ESM 16 displays the funnel plot for *ACTN3*, which did not indicate the presence of publication bias. Finally, certainty of evidence was rated as high (see Table 1).

#### 3.3.3 PPARGC1A

**FIGURE 4:**
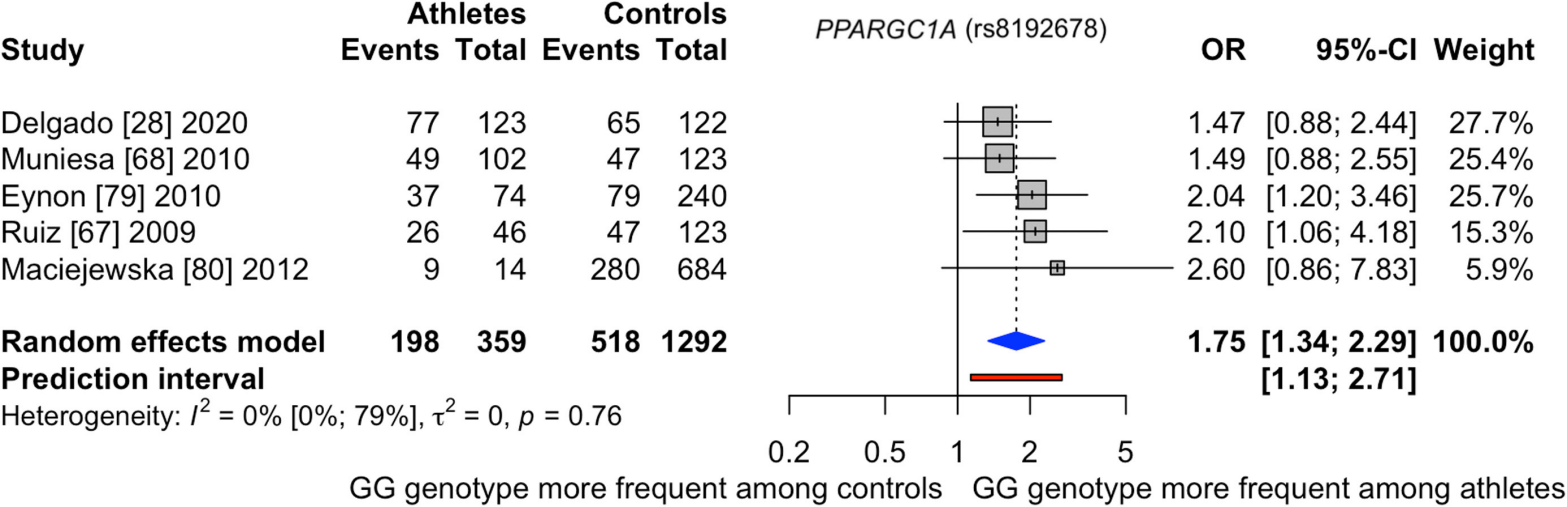
Results of the meta‐analysis investigating *PPARGC1A* (rs8192678) when comparing (inter)national runners and cyclists to controls; Events = GG genotype

ESM 17 summarizes the study characteristics of *PPARGC1A*. Five studies with in total 359 (inter)national competing Caucasian runners and cyclists and 1292 controls were investigated in the meta‐analysis for *PPARGC1A* (Figure 4).^28^, ^67^, ^68^, ^79^, ^80^ One publication was rated as low^79^ and four as unclear^28^, ^67^, ^68^, ^80^ risk of bias (ESM 18). Three articles examined runners and cyclists competing at international level^28^, ^68^, ^80^ and two studies^67^, ^79^ analyzed national and international competing runners and cyclists as one group. One report explored only runners,^79^ one only cyclists,^80^ and the remaining three articles^28^, ^67^, ^68^ analyzed runners and cyclists together.

The results showed that (inter)national competing runners and cyclists had 1.75 times the odds of carrying the GG genotype (GG vs. AG + AA) compared with controls (pooled odds ratio [95%CI]: 1.75 [1.34–2.29]). GG genotype frequency was 55.2% in the athlete and 40.1% in the control group. The between‐study heterogeneity variance was estimated at *T* ^2^ = 0.00, with an *I* ^2^ value of 0% [0.0%–79.2%]. Noteworthy, two publications might have employed the same control group but different athletes.^67^, ^68^ Excluding one or both studies did not change the result (*p* < 0.01). Lastly, publication bias was not detected (ESM 19) and certainty of evidence for *PPARGC1A* was rated high (Table 1).

#### 3.3.4 *AMPD1* and *HFE*

**FIGURE 5:**
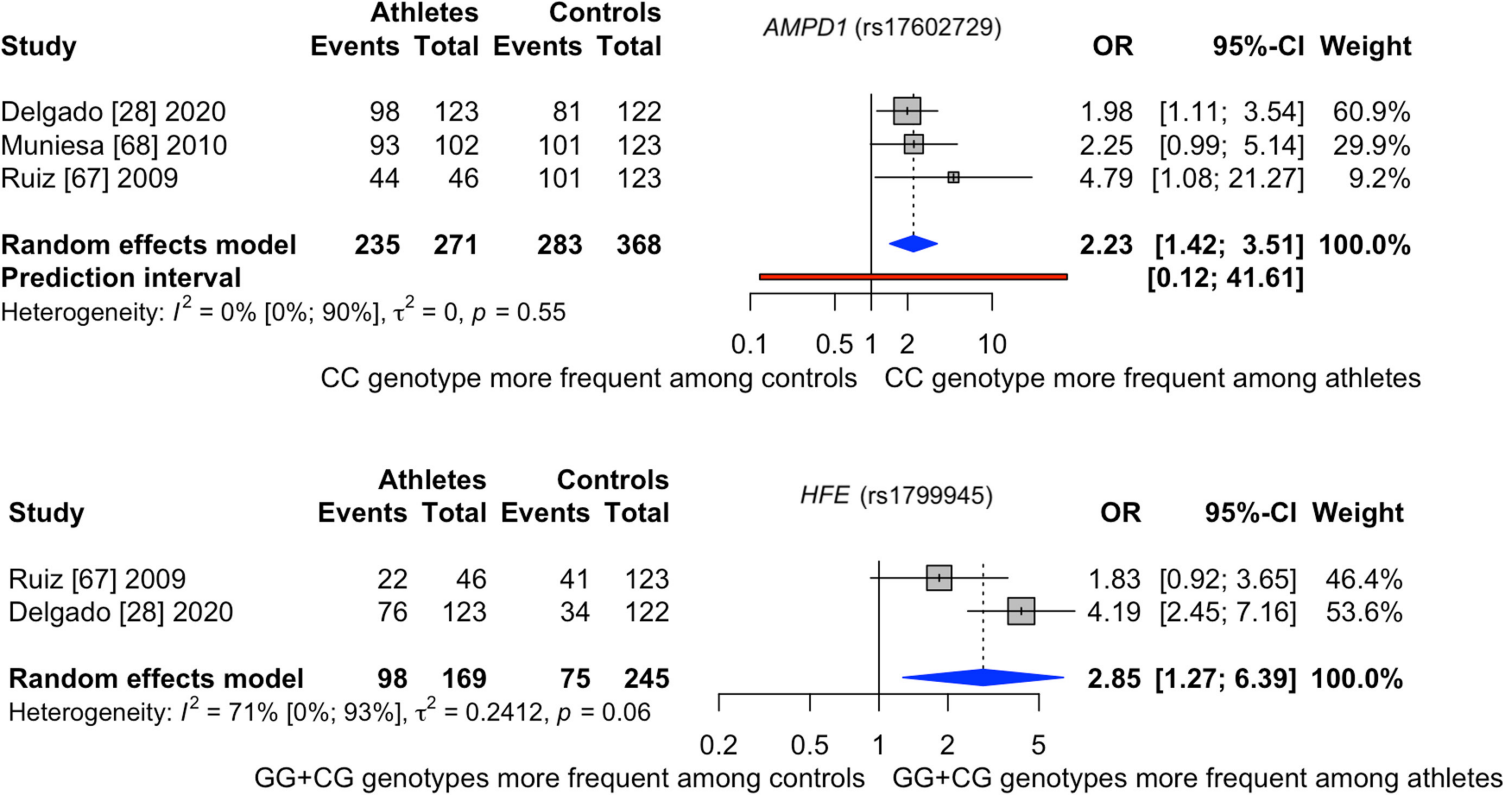
Results of the meta‐analysis investigating *AMPD1* (rs17602729) at the top and *HFE* (rs1799945) at the bottom when comparing (inter)national runners and cyclists to controls; Events = CC genotype for *AMPD1* and GG + CG genotype for *HFE*

Figure 5 presents the results for *AMPD1* ^28^, ^67^, ^68^ on the top and *HFE* ^28^, ^67^ on the bottom. ESM 20 and 22 summarizes the study characteristics of *AMPD1* and *HFE*, respectively. All publications were rated with unclear risk of bias (ESM 21 and 23) and examined male Caucasian runners and cyclists together.

For *AMPD1*, 271 (inter)national competing runners and cyclists and 368 controls were analyzed. Two studies^28^, ^68^ investigated international competing runners and cyclists. The remaining report employed national and international runners and cyclists together.^67^ (Inter)national competing runners and cyclists had significantly higher prevalence of carrying the CC genotype (CC vs. CT + TT) compared with controls (pooled odds ratio [95%CI]: 2.23 [1.42–3.51]). The frequency of the CC genotype was 86.7% in the athlete and 76.9% in the control group. The between‐study heterogeneity variance was estimated with *T* ^2^ = 0.00 and an *I* ^2^ value of 0% [0.0%–89.6%]. Two articles involved the same control group but different athletes.^67^, ^68^ Excluding one of the two studies did not change the result (*p* < 0.01). Furthermore, one control group deviated from Hardy–Weinberg equilibrium,^28^ but exclusion did not affect the finding (*p* < 0.01).

For HFE a dominant inheritance model was assumed where in total 169 runners and cyclists and 245 controls were investigated. One publication examined runners and cyclists competing internationally^28^ and one employed both national and international runners and cyclists.^67^ (Inter)national competing runners and cyclists had 2.85 times the odds of carrying the effect allele (G) (GG + CG vs. CC) when compared with controls (pooled odds ratio [95%CI]: 2.85 [1.27–6.39]). The frequency of the GG + CG genotype was 58.0% in athletes and 30.6% in controls. The between‐study heterogeneity variance was estimated with *T* ^2^ = 0.24 and an *I* ^2^ value of 70.8% [0.0%; 93.4%]. Certainty of evidence for *AMPD1* as well as for *HFE* was moderate (Table 1).

### 3.4 Sensitivity analysis

Most of the sensitivity analyses did not change our main findings (ESM 24). In sensitivity analysis 1, we applied a fixed effect model which did not change the results. In sensitivity analysis 2, in which a dominant inheritance model was assumed, only *HFE* (rs1799945) was significantly associated with (inter)national competing runners and cyclists when compared with controls. In sensitivity analysis 3, the Hartung–Knapp adjustment for random effects model was applied and *HFE* was no longer significant (*p* = 0.24). However, in addition to *ACE*, *ACTN3*, *PPARGC1A*, and *AMPD1*, two other genetic variants showed a significant association. For *MSTN* (rs1805086), (inter)national competing runners and cyclists showed a greater prevalence of carrying the G allele (GG + AG) than controls (pooled odds ratio [95%CI]: 1.40 [1.11–1.78]). For *CKMM* (rs8111989) runners and cyclists had 1.50 [1.03–2.20] times the odds of carrying the TT genotype (TT vs. TC + CC) compared with controls. Further, in sensitivity analysis 4, we excluded studies in which control groups deviated from Hardy–Weinberg equilibrium, however, sensitivity analysis 4 did not influence the results. Finally, sensitivity analysis 5 was not applied because no report in the pooled analysis encompassed high risk of bias.

## 4 DISCUSSION

The aim of this systematic review with meta‐analysis was to identify genetic variants contributing to (inter)national competing long‐distance runners and road cyclists. Based on our pooled analysis, the following five genetic variants were significantly associated with runners and cyclists competing at (inter)national level when compared with sedentary controls: *ACE* II, *ACTN3* TT (rs1815739), *PPARGC1A* GG (rs8192678), *AMPD1* CC (rs17602729), and *HFE* GG + CG (rs1799945).

### 4.1 ACE

The *ACE* insertion/deletion variant, which plays a key role in the renin‐angiotensin system, has been a major focus of sport genetics.^11^ The renin‐angiotensin system is responsible for blood pressure and electrolyte homeostasis.^90^ Individuals carrying the I allele (i.e., ID and II genotypes) demonstrate lower serum levels of the *ACE* enzyme than DD individuals.^91^ When performing endurance exercise, I allele individuals demonstrate increased capillary perfusion whereas DD individuals show decreased capillary perfusion.^92^ Accordingly, a recent meta‐analysis found a significant difference in the *ACE* I/D genotype distribution (II vs. ID+DD) between 2979 “endurance” athletes (biathlon, cycling, running, rowing, skiing, swimming, and pentathlon) and 10 048 controls (pooled odds ratio [95%CI]: 1.48 [0.30–2.67]).^93^ Another meta‐analysis from 2013 also reported that the II genotype was higher in “endurance” athletes (cycling, gymnastics, hockey, rowing, skiing, swimming, and running) compared with controls (II vs. ID+DD) (pooled odds ratio [95%CI]: 1.35 [1.17–1.55]).^12^ Based on the present meta‐analysis, involving a homogeneous group of endurance athletes (i.e., runners and cyclists), we can confirm that endurance athletes possess a higher prevalence of the II genotype compared with sedentary controls (pooled odds ratio [95%CI]: 1.42 [1.12–1.81]).

### 4.2 ACTN3

The *ACTN3* (rs1815739) TT genotype results in α‐actinin‐3 deficiency which affects the muscle's ability to generate rapid contractions.^94^, ^95^ In 2019, a meta‐analysis investigated *ACTN3* among power athletes and concluded that the C allele was associated with elite power sports.^96^ Further, a meta‐analysis from 2013 including 15 studies observed no significant association with endurance athlete status (TT vs. CT + CC; pooled odds ratio [95%CI]: 0.92 [0.68–1.25]).^12^ Finally, a meta‐analysis from 2011 also concluded that the TT genotype (TT vs. CT + CC) was not associated with endurance athlete status by employing nine European cohorts (pooled odds ratio [95%CI]: 1.11 [0.69–1.79]).^97^ The present result, that (inter)national competing runners and cyclists have a significantly higher prevalence of the TT genotype (TT vs. CT + CC) (pooled odds ratio [95%CI]: 1.66 [1.26–2.19]) contrasts with the previous findings. One explanation for the different outcome may be that we investigated a homogenous group of endurance athletes (runners and cyclists) whereas the previous meta‐analysis comprised of various sport disciplines (biathlon, cycling, gymnastics, hockey, rowing, running, and triathlon).^12^, ^97^ It is well known that runners and cyclists usually possess a larger cross‐section of slow twitch fibers when compared with other sports involving frequent sprinting and jumping.^98^ Based on our and the previous findings within different athletic groups, it seems reasonable to advise future studies to distinctively analyze homogenous groups of sport disciplines as the specific muscle fiber type involved may influence the outcome.^99^ Noteworthy, a study among 698 Caucasian elite runners (1500 m‐marathon) did not find an association between the *ACE* I/D nor the *ACTN3* (rs1815739) genotypes and personal‐best running times.^100^ This result underlines the need for other research employing physically active control groups.

### 4.3 PPARGC1A

The *PPARGC1A* polymorphism (rs8192678) has been associated with multiple functions such as mitochondrial biogenesis, energy metabolism, oxidative phosphorylation, angiogenesis, and antioxidant defense.^101^, ^102^, ^103^ Research demonstrated that the GG genotype is associated with higher expression of *PPARGC1A* mRNA levels (GG vs. AG + AA),^104^ which in turn initiates the transition of fast‐twitch muscle fibers to slow‐twitch muscle fibers.^105^ The GG genotype therefore may facilitate endurance performance by the increased expression of *PPARGC1A* mRNA levels. In the present analysis, we found a clear association of the GG genotype (GG vs. AG + AA) with (inter)national competing runners and cyclists compared with controls (pooled odds ratio [95%CI]: 1.75 [1.34–2.29]). This is in accordance with the results of two recent meta‐analyses involving various “endurance” disciplines (canoeing, orienteering, running, rowing, speed skating, triathlon, and water polo).^101^, ^106^ Interestingly, a study from 2020 demonstrated that 107 Japanese non‐active women possessing the AA genotype were associated with increased proportions of oxidative muscle fibers compared with women carrying the AG + GG genotype.^107^ A reason for the discrepant results between the aforementioned study and our finding might be due to the different ethnicities investigated (Asians vs. Caucasians).

### 4.4 AMPD1

A recent review highlighted 16 polymorphisms potentially associated with marathon running performance, however, most of the results have not been replicated.^108^ Of the 16 polymorphisms, based on our approach encompassing strict inclusion criteria (i.e., runners, cyclists, and sedentary controls), we can confirm next to *ACE* only one polymorphism (*AMPD1* (rs17602729)) to be associated with (inter)national performance level of runners and cyclists. By pooling three cohorts, we found that (inter)national competing runners and cyclists had a higher prevalence of the CC genotype (CC vs. CT + TT) compared with controls (pooled odds ratio [95%CI]: 2.23 [1.42–3.51]). The *AMPD1* polymorphism plays an important role in energy metabolism and is a key enzyme necessary to produce adenosine triphosphate (ATP). ATP is the main molecule responsible to store and transfer energy in cells.^109^, ^110^

### 4.5 HFE

The *HFE* gene regulates iron absorption and individuals carrying one or two mutations (GG or CG) of the rs1799945 polymorphism show higher circulating iron concentrations than individuals without mutation.^111^ Circulating iron level is well known for its association with oxygen transport and endurance performance.^112^, ^113^ The main role of iron is to transport oxygen within the red blood cells and a normal level of iron is critical to maintain the electron transfer to produce mitochondrial energy.^114^, ^115^ A recent meta‐analysis pooled three publications analyzing the rs1799945 (*HFE*) polymorphism and “endurance” athletes revealing a higher prevalence of the G allele (GG + CG vs. CC) in athletes compared with controls (pooled odds ratio [95%CI]: 1.96 [1.58–2.45]).^116^ This is in accordance with our result for HFE (pooled odds ratio [95%CI]: 2.85 [1.27–6.39]). Nonetheless, our finding warrants careful interpretation since it was based on two reports only.

### 4.6 Strengths and limitations

We would like to highlight several strengths of this systematic review and meta‐analysis. (i) This is the first comprehensive systematic review with meta‐analysis identifying genetic variants associated with (inter)national competing long‐distance runners and road cyclists. (ii) We only included leg‐dominated disciplines and excluded whole‐body sports such as triathlon, rowing, or cross‐country skiing. This distinction is important since differences in muscle mass, oxygen extraction,^21^ contractile properties of muscle fiber,^117^ and glucose and lipid oxidative capacity^20^, ^21^, ^118^, ^119^, ^120^ between the upper and lower body require different training stimuli for adaptation. Grouping different sport disciplines together increases the inter‐cohort phenotypic variability.^19^ Performing a meta‐analysis is an effective method to increase statistical power and to analyze a homogenous group of athletes. (iii) In the present analysis, we employed strict inclusion and exclusion criteria thereby stimulating high internal validity, however, differences between and within runners and cyclists competing in various distances and performance levels cannot be fully ruled out. Furthermore, the results are likely to apply to other leg‐dominated endurance disciplines such as race walking. (iv) We performed thorough sensitivity analyses which confirmed the robustness of our main results. (v) Only two of the 13 pooled results were graded with a low certainty of evidence. The GRADE approach increases transparency and was graded independently by two reviewers. (vi) Lastly, the review process, data extraction, and risk of bias assessment were conducted by two independent reviewers with an agreement of 100%.

We also would like to acknowledge some limitations. (i) Most publications in the field of sport genetics make use of a candidate gene approach in which replication of results is often lacking.^10^, ^121^ We also found 29 genetic variants that were not replicated, at least not within (inter)national competing runners and cyclists. To date, only a handful of genome wide association studies have been conducted.^15^, ^16^, ^122^, ^123^, ^124^ Genome wide association studies require large sample sizes which is challenging within the small group of (inter)national competing athletes. Unfortunately, we had to exclude all genome wide association studies due to the variety of included sport disciplines. In addition, one case–control study investigating >30 polymorphisms within five genes (*PPP3CA*, *PPP3CB*, *PPP3CC*, *PPP3R1*, and *PPP3R2*) between 123 elite runners and 125 healthy controls has been excluded due to efficiency reasons.^60^ The authors found two polymorphisms (rs3804358 in the *PPP3CA* gene and rs3763679 in the *PPP3CB* gene) significantly associated with elite endurance athlete status in Han Chinese, but not in Caucasians. (ii) Most of the study subjects included in this review were male, and due to this sample sex imbalance, we were not able to analyze male and female athletes apart. In future, both sexes with adequate sample sizes for both should be investigated.^125^ (iii) In sport genetic studies, the physiological, anthropometric, or biomechanical characteristics of athletes are often not well described. Consequently, the definition of performance level remains ambiguous. Further, we excluded articles in which detailed information (e.g., sport discipline, performance level, or genotype frequency) could not be retrieved after contacting the authors. Therefore, a small number of potentially relevant articles could have been missed. (iv) We analyzed cohorts with different ethnicities, which could result in biases because of population stratification. However, in the present analysis, 38 of 43 studies (88%) employed Caucasians and when excluding non‐Caucasian cohorts, the odds ratios remained constant. (v) When assessing the risk of bias, only two reports^73^, ^126^ performed a power calculation. The low statistical power due to the relatively small number of athletes available is a common problem in exercise genetics and the resulting unbalanced number of athletes and controls in the current meta‐analysis lowers the generalizability of the findings. In addition, we identified two publications that most likely included identical athletes and controls but reported different genotype frequencies for *AGT* (rs699).^69^, ^127^ (vi) Furthermore, we merely searched two databases and included articles published in peer reviewed journals potentially leading to publication bias. Although funnel plots did not indicate the presence of publication bias. (vii) The control group consisted of sedentary individuals (i.e., non‐athletes), and it cannot be ruled out that controls (when engaging in training) could potentially reach (inter)national performance level. (viii) Finally, a common pitfall within exercise genetics is the inconsistent reporting of genes and/or alleles, which we also encountered when analyzing the *ACE* and *BDKRB2* gene. Future studies should adopt reporting genes consistently using rs‐numbers and correct allele forms.

### 4.7 Perspective

It seems that (inter)national competing runners and cyclists constitute a unique genetic make‐up, which likely contribute to the high level of performance. We advise future studies to analyze homogenous group of athletes as well as different types of control groups (e.g., physically active adults, power athletes) and to keep the necessity of sex balance in mind. It is important to emphasize that the observed associations between the genetic variants and the endurance performance level do not necessarily equate causation.

## CONFLICT OF INTEREST

Magdalena Johanna Konopka, Jorn Carlos Maria Leonardus van den Bunder, Gerard Rietjens, Billy Sperlich, and Maurice Petrus Zeegers declare that they have no conflict of interest.

## Supporting information


Appendix S1

## Data Availability

The template data collection form, the data used for all analyses, and the analytic code are available at: www.dataverse.nl.
