# Supplementary material for: Genetics of long‐distance runners and road cyclists—A systematic review with meta‐analysis
Source: Scand J Med Sci Sports. 2022 Jul 26;32(10):1414–29. doi: 10.1111/sms.14212 (PMC9544934; doi:10.1111/sms.14212)
Supplement: Supplementary file 1 — Appendix S1 [file SMS-32-1414-s001.docx]

**Electronic supplementary material**

**Genetics of long-distance runners and road cyclists - a systematic review with meta-analysis**

Magdalena Johanna Konopka^1,2^, Jorn Carlos Maria Leonardus van den Bunder^2^, Gerard Rietjens^3^, Billy Sperlich^4^, Maurice Petrus Zeegers^1,2,5^

1 Care and Public Health Research Institute (CAPHRI), Maastricht University, Maastricht, the Netherlands

2 Department of Epidemiology, Maastricht University Medical Centre, Maastricht, the Netherlands

3 Department of Human Physiology and Sports Medicine, Vrije Universiteit Brussel, Brussels, Belgium

4 Integrative & Experimental Exercise Science & Training, Institute of Sport Science, University of Würzburg, Würzburg, Germany

5 School of Nutrition and Translational Research in Metabolism (NUTRIM), Maastricht University, Maastricht, the Netherlands

Corresponding author: Magdalena Johanna Konopka

E-mail address: [magdalena.konopka@maastrichtuniversity.nl](mailto:magdalena.konopka@maastrichtuniversity.nl)

**TABLE OF CONTENTS**

[Supplementary 1. PRISMA checklist. 3](#_Toc101356627)

[Supplementary 2. Search terms. 6](#_Toc101356628)

[Supplementary 3. Full search strategy Medline. 7](#_Toc101356629)

[Supplementary 4. Full search strategy Embase. 8](#_Toc101356630)

[Supplementary 5. Journals of included studies. 9](#_Toc101356631)

[Supplementary 6. Reason for exclusion. 10](#_Toc101356632)

[Supplementary 7. Key characteristics. 23](#_Toc101356633)

[Supplementary 8. Allele & genotype frequencies. 27](#_Toc101356634)

[Supplementary 9. Risk of bias assessment. 31](#_Toc101356635)

[Supplementary 10. Unique polymorphisms (no pooling). 32](#_Toc101356636)

[Supplementary 11. Characteristics of studies analyzing *ACE* I/D. 35](#_Toc101356637)

[Supplementary 12. Risk of bias assessment of *ACE* I/D. 36](#_Toc101356638)

[Supplementary 13. Funnel plot *ACE* I/D. 37](#_Toc101356639)

[Supplementary 14. Characteristics of studies analyzing *ACTN3* (rs1815739). 38](#_Toc101356640)

[Supplementary 15. Risk of bias assessment of *ACTN3* (rs1815739). 39](#_Toc101356641)

[Supplementary 16. Funnel plot *ACTN3* (rs1815739). 40](#_Toc101356642)

[Supplementary 17. Characteristics of studies analyzing *PPARGC1A* (rs8192678). 41](#_Toc101356643)

[Supplementary 18. Risk of bias assessment of *PPARGC1A* (rs8192678). 42](#_Toc101356644)

[Supplementary 19. Funnel plot *PPARGC1A* (rs8192678). 43](#_Toc101356645)

[Supplementary 20. Characteristics of studies analyzing *AMPD1* (rs17602729). 44](#_Toc101356646)

[Supplementary 21. Risk of bias assessment *AMPD1* (rs17602729). 45](#_Toc101356647)

[Supplementary 22. Characteristics of studies analyzing *HFE* (rs1799945). 46](#_Toc101356648)

[Supplementary 23. Risk of bias assessment HFE (rs1799945). 47](#_Toc101356649)

[Supplementary 24. Sensitivity analysis. 48](#_Toc101356650)

[REFERENCES 49](#_Toc101356651)

# **Supplementary 1.** PRISMA checklist.

| Section and Topic | Item # | Checklist item | Location where item is reported |
| --- | --- | --- | --- |
| TITLE | | | Page |
| Title | 1 | Identify the report as a systematic review. | 1 |
| ABSTRACT | | |  |
| Abstract | 2 | See the PRISMA 2020 for Abstracts checklist. | 2 |
| INTRODUCTION | | |  |
| Rationale | 3 | Describe the rationale for the review in the context of existing knowledge. | 3 |
| Objectives | 4 | Provide an explicit statement of the objective(s) or question(s) the review addresses. | 3 |
| METHODS | | |  |
| Eligibility criteria | 5 | Specify the inclusion and exclusion criteria for the review and how studies were grouped for the syntheses. | 3 |
| Information sources | 6 | Specify all databases, registers, websites, organisations, reference lists and other sources searched or consulted to identify studies. Specify the date when each source was last searched or consulted. | 4 |
| Search strategy | 7 | Present the full search strategies for all databases, registers and websites, including any filters and limits used. | Supplement 3, 4 |
| Selection process | 8 | Specify the methods used to decide whether a study met the inclusion criteria of the review, including how many reviewers screened each record and each report retrieved, whether they worked independently, and if applicable, details of automation tools used in the process. | 4 |
| Data collection process | 9 | Specify the methods used to collect data from reports, including how many reviewers collected data from each report, whether they worked independently, any processes for obtaining or confirming data from study investigators, and if applicable, details of automation tools used in the process. | 4 |
| Data items | 10a | List and define all outcomes for which data were sought. Specify whether all results that were compatible with each outcome domain in each study were sought (e.g. for all measures, time points, analyses), and if not, the methods used to decide which results to collect. | 4 |
|  | 10b | List and define all other variables for which data were sought (e.g. participant and intervention characteristics, funding sources). Describe any assumptions made about any missing or unclear information. | 4 |
| Study risk of bias assessment | 11 | Specify the methods used to assess risk of bias in the included studies, including details of the tool(s) used, how many reviewers assessed each study and whether they worked independently, and if applicable, details of automation tools used in the process. | 4 |
| Effect measures | 12 | Specify for each outcome the effect measure(s) (e.g. risk ratio, mean difference) used in the synthesis or presentation of results. | 4 |
| Synthesis methods | 13a | Describe the processes used to decide which studies were eligible for each synthesis (e.g. tabulating the study intervention characteristics and comparing against the planned groups for each synthesis (item #5)). | 4 |
|  | 13b | Describe any methods required to prepare the data for presentation or synthesis, such as handling of missing summary statistics, or data conversions. | 4 |
|  | 13c | Describe any methods used to tabulate or visually display results of individual studies and syntheses. | 4 |
|  | 13d | Describe any methods used to synthesize results and provide a rationale for the choice(s). If meta-analysis was performed, describe the model(s), method(s) to identify the presence and extent of statistical heterogeneity, and software package(s) used. | 4 |
|  | 13e | Describe any methods used to explore possible causes of heterogeneity among study results (e.g. subgroup analysis, meta-regression). | 4 |
|  | 13f | Describe any sensitivity analyses conducted to assess robustness of the synthesized results. | 4 |
| Reporting bias assessment | 14 | Describe any methods used to assess risk of bias due to missing results in a synthesis (arising from reporting biases). | 4 |
| Certainty assessment | 15 | Describe any methods used to assess certainty (or confidence) in the body of evidence for an outcome. | 4 |
| RESULTS | | |  |
| Study selection | 16a | Describe the results of the search and selection process, from the number of records identified in the search to the number of studies included in the review, ideally using a flow diagram. | 5 |
|  | 16b | Cite studies that might appear to meet the inclusion criteria, but which were excluded, and explain why they were excluded. | 5 |
| Study characteristics | 17 | Cite each included study and present its characteristics. | Supplement 7 |
| Risk of bias in studies | 18 | Present assessments of risk of bias for each included study. | Supplement 9 |
| Results of individual studies | 19 | For all outcomes, present, for each study: (a) summary statistics for each group (where appropriate) and (b) an effect estimate and its precision (e.g. confidence/credible interval), ideally using structured tables or plots. | Supplement 7 |
| Results of syntheses | 20a | For each synthesis, briefly summarise the characteristics and risk of bias among contributing studies. | Supplement 11-23 |
|  | 20b | Present results of all statistical syntheses conducted. If meta-analysis was done, present for each the summary estimate and its precision (e.g. confidence/credible interval) and measures of statistical heterogeneity. If comparing groups, describe the direction of the effect. | 7-8 |
|  | 20c | Present results of all investigations of possible causes of heterogeneity among study results. | 7-8 |
|  | 20d | Present results of all sensitivity analyses conducted to assess the robustness of the synthesized results. | 12-13 |
| Reporting biases | 21 | Present assessments of risk of bias due to missing results (arising from reporting biases) for each synthesis assessed. | Supplement 13,16,19 |
| Certainty of evidence | 22 | Present assessments of certainty (or confidence) in the body of evidence for each outcome assessed. | 7-8 |
| DISCUSSION | | |  |
| Discussion | 23a | Provide a general interpretation of the results in the context of other evidence. | 13-14 |
|  | 23b | Discuss any limitations of the evidence included in the review. | 14 |
|  | 23c | Discuss any limitations of the review processes used. | 14 |
|  | 23d | Discuss implications of the results for practice, policy, and future research. | 15 |
| OTHER INFORMATION | | |  |
| Registration and protocol | 24a | Provide registration information for the review, including register name and registration number, or state that the review was not registered. | 15 |
|  | 24b | Indicate where the review protocol can be accessed, or state that a protocol was not prepared. | 15 |
|  | 24c | Describe and explain any amendments to information provided at registration or in the protocol. | 15 |
| Support | 25 | Describe sources of financial or non-financial support for the review, and the role of the funders or sponsors in the review. | 15 |
| Competing interests | 26 | Declare any competing interests of review authors. | 15 |
| Availability of data, code and other materials | 27 | Report which of the following are publicly available and where they can be found: template data collection forms; data extracted from included studies; data used for all analyses; analytic code; any other materials used in the review. | 15 |

# **Supplementary 2.** Search terms.

| Category | Search terms |
| --- | --- |
| Outcome | athlete status  elite athlete  professional athlete  master athlete  athlete |
| Athlete characteristics | endurance  endurance performance  athletic performance  maximal oxygen consumption  peak oxygen uptake  VO2max  VO2 max  VO2 peak  VO2peak  lactate threshold  aerobic capacity  aerobic fitness  exercise capacity  economy of movement  cardiorespiratory fitness  cardiovascular fitness |
| Study design | genome wide association study  gwas  candidate gene study  genetics |
| Intervention | single nucleotide polymorphism  “snp”  genetic polymorphism  polymorphism  gene  allele  genetic variant  genomic predictor  *ACE*  *ACTN3*  *PPARA*  *HFE*  *PPARGC1A*  *PPARD*  *AQP1* |

# **Supplementary 3.** Full search strategy Medline.

| SEARCH | Search strategy Medline (filter: most recent)  Search performed on 15-11-2021 | N |
| --- | --- | --- |
| 1 | ((((((athlete) OR (elite athlete)) OR (professional athlete)) OR (master athlete)) OR (athlete status)) AND (((((((((((((((((endurance) OR (endurance performance)) OR (athletic performance)) OR (maximal oxygen consumption)) OR (peak oxygen uptake)) OR (VO2max)) OR (VO2 max)) OR (VO2 peak)) OR (VO2peak)) OR (lactate threshold)) OR (aerobic capacity)) OR (aerobic fitness)) OR (exercise capacity)) OR (economy of movement)) OR (cardiorespiratory fitness)) OR (cardiovascular fitness)))) AND ((((((((((((genome wide association study) OR (gwas)) OR (candidate gene study)) OR (single nucleotide polymorphism)) OR ("snp")) OR (genetic polymorphism)) OR (polymorphism)) OR (gene)) OR (allele)) OR (genetic variant)) OR (genomic predictor))) | 2454 |
| 2 | (athlete status) AND (((single nucleotide polymorphism) AND ("snp")) OR (genetics)) | 915 |
| 3 | ((ACE OR ACTN3 OR (PPARA) OR HFE OR PPARGC1A OR PPARD OR AQP1) AND (athlete) AND endurance) | 404 |
| Total |  | **3773** |

No filters were applied during the search.

# **Supplementary 4.** Full search strategy Embase.

| SEARCH | Search strategy Embase  Search performed on 15-11-2021 | N |
| --- | --- | --- |
| 1 | ((((((athlete) OR (elite athlete)) OR (professional athlete)) OR (master athlete)) OR (athlete status)) AND (((((((((((((((((endurance) OR (endurance performance)) OR (athletic performance)) OR (maximal oxygen consumption)) OR (peak oxygen uptake)) OR (VO2max)) OR (VO2 max)) OR (VO2 peak)) OR (VO2peak)) OR (lactate threshold)) OR (aerobic capacity)) OR (aerobic fitness)) OR (exercise capacity)) OR (economy of movement)) OR (cardiorespiratory fitness)) OR (cardiovascular fitness)))) AND ((((((((((((genome wide association study) OR (gwas)) OR (candidate gene study)) OR (single nucleotide polymorphism)) OR ("snp")) OR (genetic polymorphism)) OR (polymorphism)) OR (gene)) OR (allele)) OR (genetic variant)) OR (genomic predictor))) | 661 |
| 2 | (athlete status) AND (((single nucleotide polymorphism) AND ("snp")) OR (genetics)) | 47 |
| 3 | ((ACE OR ACTN3 OR (PPARA) OR HFE OR PPARGC1A OR PPARD OR AQP1) AND (athlete) AND endurance) | 146 |
| Total |  | **854** |

No filters were applied during the search.

# **Supplementary 5.** Journals of included studies.

| Journal | Frequency |
| --- | --- |
| Advanced Biomedical Research | 1 |
| Applied Physiology Nutrition and Metabolism | 2 |
| British Journal of Sports medicine | 2 |
| European Journal of Applied Physiology | 1 |
| European Journal of Human Genetics | 2 |
| European Journal of sport science | 1 |
| Experimental physiology | 4 |
| Free Radical Research | 1 |
| Frontiers in Physiology | 1 |
| Genes | 1 |
| Growth Hormone & IGF Research | 2 |
| Human Biology | 1 |
| International Journal of Sports Medicine | 5 |
| Journal of Applied Physiology | 3 |
| Journal of Science and Medicine in Sports | 1 |
| Journal of Sports Sciences | 1 |
| Journal of Strength and Conditioning Research | 2 |
| Medicine & Science in Sports & Exercise | 1 |
| Metabolism: Clinical and experimental | 1 |
| Physiological Genomics | 1 |
| PLOS One | 1 |
| Scandinavian Journal of Medicine & Science in Sports | 3 |
| Sports Medicine | 2 |
| The Journal of Physiological Sciences | 1 |
| The Journal of Physiology | 1 |
| The Journal of Sports Medicine and physical fitness | 1 |

# **Supplementary 6.** Reason for exclusion of articles based on full text screening (n=226) including the articles not retrievable (n=3), sorted alphabetically by year.

| First author | Year | Title | Reason for exclusion | Details |
| --- | --- | --- | --- | --- |
| Bouchard | 1989 | Muscle genetic variants and relationship with performance and trainability | Performance level | athlete level not defined |
| Rivera | 1997 | Muscle-specific creatine kinase gene polymorphisms in elite endurance athletes and sedentary controls | Sport disciplines | included biathletes, triathletes etc. |
| Gayagay | 1998 | Elite endurance athletes and the ACE I allele--the role of genes in athletic performance | Sport disciplines | included rowers |
| Montgomery | 1998 | Human gene for physical performance | Sport disciplines | included mountaineers |
| Rivera | 1998 | Three mitochondrial DNA restriction polymorphisms in elite endurance athletes and sedentary controls | Sport disciplines | included biathletes, triathletes etc. |
| Taylor | 1999 | Elite athletes and the gene for angiotensin-converting enzyme | Sport disciplines | included swimmers, rowers etc. |
| Rankinen | 2000 | No association between the angiotensin-converting enzyme ID polymorphism and elite endurance athlete status | Sport disciplines | included cross-country skiers, biathletes etc. |
| Wolfarth | 2000 | A polymorphism in the alpha2a-adrenoceptor gene and endurance athlete status | Sport disciplines | sport disciplines not specified |
| Klissouras | 2001 | Genes and olympic performance: a co-twin study | Sport disciplines | included race walkers |
| Moore | 2001 | Obesity gene variant and elite endurance performance | Performance level | athlete level not defined |
| Woods | 2001 | Elite swimmers and the D allele of the ACE I/D polymorphism | Sport disciplines | included swimmers |
| Deugnier | 2002 | Increased body iron stores in elite road cyclists | Genotype frequency | genotype frequency not reported |
| Data | 2003 | eNOS T-786C genotype, physical activity, and peak forearm blood flow in females | Performance level | athlete level not defined |
| Kang | 2003 | The protein polymorphism of haptoglobin in Korean elite athletes | Control definition | no control group |
| Yang | 2003 | ACTN3 genotype is associated with human elite athletic performance | Sport disciplines | included swimmers, rowers etc. |
| Yoshioka | 2003 | Serial analysis of gene expression in the skeletal muscle of endurance athletes compared to sedentary men | Sport disciplines | included triathletes |
| Zanoteli | 2003 | Deficiency of muscle alpha-actinin-3 is compatible with high muscle performance | Performance level | athlete level not defined |
| Chicharro | 2004 | Mutations in the hereditary haemochromatosis gene HFE in professional endurance athletes | Sport disciplines | sport disciplines not specified |
| Collins | 2004 | The ACE gene and endurance performance during the South African Ironman Triathlons | Sport disciplines | included triathletes |
| Hudson | 2004 | The -55 C/T polymorphism within the UCP3 gene and performance during the South African Ironman Triathlon | Sport disciplines | included triathletes |
| Tsianos | 2004 | The ACE gene insertion/deletion polymorphism and elite endurance swimming | Control definition | included swimmers |
| Turgut | 2004 | The angiotensin converting enzyme I/D polymorphism in Turkish athletes and sedentary controls | Sport disciplines | included basketball players, football players etc. |
| Williams | 2004 | Bradykinin receptor gene variant and human physical performance | Genotype frequency | Genotype frequency not reported for >5000m runners |
| Henderson | 2005 | The EPAS1 gene influences the aerobic-anaerobic contribution in elite endurance athletes | Sport disciplines | included <5000m runners, rowers, etc. |
| Lucia | 2005 | PPARGC1A genotype (Gly482Ser) predicts exceptional endurance capacity in European men | Sport disciplines | sport disciplines not specified (middle to long distance track athletes) |
| Lucía | 2005 | Is there an association between ACE and CKMM polymorphisms and cycling performance status during 3-week races? | Genotype frequency | genotype frequency not reported, included <5000m runners |
| Niemi | 2005 | Mitochondrial DNA and ACTN3 genotypes in Finnish elite endurance and sprint athletes | Sport disciplines | included <5000m runners |
| Rubio | 2005 | Frequency of the C34T mutation of the AMPD1 gene in world-class endurance athletes: does this mutation impair performance? | Sport disciplines | included <5000m runners |
| Scott | 2005 | Mitochondrial DNA lineages of elite Ethiopian athletes | Genotype frequency | genotype frequency not reported |
| Scott | 2005 | No association between Angiotensin Converting Enzyme (ACE) gene variation and endurance athlete status in Kenyans | Sport disciplines | included <5000m runners |
| Ahmetov | 2006 | PPARalpha gene variation and physical performance in Russian athletes | Performance level | included regional level athletes |
| Dékány | 2006 | The role of insertion allele of angiotensin converting enzyme gene in higher endurance efficiency and some aspects of pathophysiological and drug effects | Sport disciplines | included triathletes, rowers etc. |
| Hruskovicová | 2006 | The angiotensin converting enzyme I/D polymorphism in long distance runners | Sport disciplines | included inline-skaters, performance level not specified |
| Lucia | 2006 | C34T mutation of the AMPD1 gene in an elite white runner | Case study | case study |
| Saunders | 2006 | The bradykinin beta 2 receptor (BDKRB2) and endothelial nitric oxide synthase 3 (NOS3) genes and endurance performance during Ironman Triathlons | Sport disciplines | included triathletes |
| Walpole | 2006 | Growth hormone 1 (GH1) gene and performance and post-race rectal temperature during the South African Ironman triathlon | Sport disciplines | included triathletes |
| Castro | 2007 | Mitochondrial haplogroup T is negatively associated with the status of elite endurance athlete | Sport disciplines | included rowers |
| De Moor | 2007 | Genome-wide linkage scan for athlete status in 700 British female DZ twin pairs | Performance level | self-reported performance level,  included swimmers, gymnasts, etc. |
| McConell | 2007 | Skeletal muscle nNOS mu protein content is increased by exercise training in humans | Performance level | VO2max<71 ml/kg/min,  included triathletes |
| Oh | 2007 | The distribution of I/D polymorphism in the ACE gene among Korean male elite athletes | Sport disciplines | included basketball players, soccer players etc. |
| Paparini | 2007 | ACTN3 genotyping by real-time PCR in the Italian population and athletes | Sport disciplines | included rowers |
| Saunders | 2007 | No association of the ACTN3 gene R577X polymorphism with endurance performance in Ironman Triathlons | Sport disciplines | included triathletes |
| Wolfarth | 2007 | Association between a beta2-adrenergic receptor polymorphism and elite endurance performance | Sport disciplines | included biathletes, rowers etc. |
| Yang | 2007 | The ACTN3 R577X polymorphism in East and West African athletes | Sport disciplines | included <5000m runners |
| He | 2008 | Is there an association between PPARGC1A genotypes and endurance capacity in Chinese men? | Sport disciplines | included soldiers |
| He | 2008 | NRF-1 genotypes and endurance exercise capacity in young Chinese men | Sport disciplines | included soldiers |
| Papadimitriou | 2008 | The ACTN3 gene in elite Greek track and field athletes | Sport disciplines | included <5000m runners, triathletes etc. |
| Santiago | 2008 | ACTN3 genotype in professional soccer players | Genotype frequency | genotype frequency not reported |
| Wolfarth | 2008 | Endothelial nitric oxide synthase gene polymorphism and elite endurance athlete status: the Genathlete study | Sport disciplines | included cross-country skiers, biathletes etc. |
| Ahmetov | 2009 | Association of the VEGFR2 gene His472Gln polymorphism with endurance-related phenotypes | Performance level | included regional level athletes |
| Ahmetov | 2009 | The combined impact of metabolic gene polymorphisms on elite endurance athlete status and related phenotypes | Sport disciplines | included swimmers, triathletes etc. |
| Bentley | 2009 | The relationship between monocarboxylate transporters 1 and 4 expression in skeletal muscle and endurance performance in athletes | Sport disciplines | athlete level not specified |
| Cieszczyk | 2009 | The angiotensin converting enzyme gene I/D polymorphism in Polish rowers | Sport disciplines | included rowers |
| Costa | 2009 | Angiotensin-converting enzyme genotype affects skeletal muscle strength in elite athletes | Sport disciplines | included swimmers and triathletes |
| Costa | 2009 | Association between ACE D allele and elite short distance swimming | Sport disciplines | included swimmers |
| de Milander | 2009 | The interleukin-6, serotonin transporter, and monoamine oxidase A genes and endurance performance during the South African Ironman Triathlon | Sport disciplines | included triathletes |
| Gómez-Gallego | 2009 | The -786 T/C polymorphism of the NOS3 gene is associated with elite performance in power sports | Duplicate study population | See reference 89 |
| Gonzalez-Freire | 2009 | Unique among unique. Is it genetically determined? | Case study | case study |
| Juffer | 2009 | Genotype distributions in top-level soccer players: a role for ACE? | Genotype frequency | included soccer players |
| Min | 2009 | Is there a gender difference between ACE gene and race distance? | Sport disciplines | included <5000m runners |
| Papadimitriou | 2009 | The ACE I/D polymorphism in elite Greek track and field athletes | Sport disciplines | included <5000m runners |
| Scott | 2009 | Mitochondrial haplogroups associated with elite Kenyan athlete status | No nuclear genome | MtDNA, included <5000m runners |
| Ahmetov | 2010 | The ACTN3 R577X polymorphism in Russian endurance athletes | Performance level | included regional level athletes |
| Boraita | 2010 | Cardiovascular adaptation, functional capacity and Angiotensin-converting enzyme I/D polymorphism in elite athletes | Control definition | no control group |
| Cauci | 2010 | Variable number of tandem repeat polymorphisms of the interleukin-1 receptor antagonist gene IL-1RN: a novel association with the athlete status | Sport disciplines | sport disciplines not specified |
| Doring | 2010 | ACTN3 R577X and other polymorphisms are not associated with elite endurance athlete status in the Genathlete study | Sport disciplines | included cross-country skiers and biathletes |
| Döring | 2010 | A common haplotype and the Pro582Ser polymorphism of the hypoxia-inducible factor-1alpha (HIF1A) gene in elite endurance athletes | Sport disciplines | included cross-country skiers and biathletes |
| Santiago | 2010 | Does the polygenic profile determine the potential for becoming a world-class athlete? Insights from the sport of rowing | Sport disciplines | included rowers |
| Shang | 2010 | Association between the ACTN3 R577X polymorphism and female endurance athletes in China | Sport disciplines | included swimmers and rowers |
| Tamura | 2010 | Preliminary report: mitochondrial DNA 5178 polymorphism in male elite Japanese endurance runners | Control definition | control group were athletes |
| Tsianos | 2010 | Associations of polymorphisms of eight muscle- or metabolism-related genes with performance in Mount Olympus marathon runners | Performance level | athlete level not defined |
| Ahmetov | 2011 | The dependence of preferred competitive racing distance on muscle fibre type composition and ACTN3 genotype in speed skaters | Sport disciplines | included skaters |
| Brown | 2011 | The COL5A1 gene, ultra-marathon running performance, and range of motion | Control definition | no control group |
| Buxens | 2011 | Can we predict top-level sports performance in power vs endurance events? A genetic approach | Control definition | control group were athletes |
| Chiu | 2011 | ACTN3 genotype and swimming performance in Taiwan | Sport disciplines | included swimmers |
| Ciȩszczyk | 2011 | Is the C34T polymorphism of the AMPD1 gene associated with athlete performance in rowing? | Sport disciplines | included rowers |
| Döring | 2011 | Single nucleotide polymorphisms in the myostatin (MSTN) and muscle creatine kinase (CKM) genes are not associated with elite endurance performance | Sport disciplines | included cross-country skiers, biathletes etc |
| Eynon | 2011 | Mitochondrial biogenesis related endurance genotype score and sports performance in athletes | Duplicate study population | See reference 79, 128-130 |
| Eynon | 2011 | Physiological variables and mitochondrial-related genotypes of an athlete who excels in both short and long-distance running | Case study | case study |
| Fiuza-Luces | 2011 | Are 'endurance' alleles 'survival' alleles? Insights from the ACTN3 R577X polymorphism | Genotype frequency | Genotype frequency not reported |
| He | 2011 | Are calcineurin genes associated with athletic status? A function, replication study | More than 30 polymorphisms analyzed | 55 polymorphisms in 5 genes investigated |
| Kothari | 2011 | ACTN3 R577X polymorphism in Asian Indian athletes. | Sport disciplines | included <5000m runners, swimmers etc. |
| Maciejewska | 2011 | Variation in the PPARα gene in Polish rowers | Sport disciplines | included rowers |
| Mikami | 2011 | Mitochondrial haplogroups associated with elite Japanese athlete status | Sport disciplines | included <5000m runners and sailors |
| Nogales-Gadea | 2011 | Are mitochondrial haplogroups associated with elite athletic status? A study on a Spanish cohort | Sport disciplines | included <5000m runners |
| O'Connell | 2011 | COL6A1 gene and Ironman triathlon performance | Control definition | no control group |
| Posthumus | 2011 | The COL5A1 gene: a novel marker of endurance running performance | Control definition | no control group |
| Smith | 2011 | Genetic variation and endurance running: Comparing ACE1 and ACTN3 polymorphisms in marathon runners and sprinters | Conference abstract | Conference abstract |
| Cięszczyk | 2012 | Variation in the HIF1A gene in elite rowers | Sport disciplines | included rowers |
| Eynon | 2012 | The ACTN3 R577X polymorphism across three groups of elite male European athletes | Sport disciplines | included water polo players |
| Kim | 2012 | MtDNA haplogroups and elite Korean athlete status | Sport disciplines | included handball players, hockey players etc. |
| Kothari | 2012 | Molecular analysis of genetic variation in angiotensin I-converting enzyme identifies no association with sporting ability: First report from Indian population | Sport disciplines | included <5000m runners, swimmers etc. |
| Kunorozva | 2012 | Chronotype and PERIOD3 variable number tandem repeat polymorphism in individual sports athletes | Performance level | athlete level described as well-trained |
| Maciejewska | 2012 | The PPARGC1A gene Gly482Ser in Polish and Russian athletes | Sport disciplines | included triathletes,  cross-country skiers etc. |
| Maciejewska-Karłowska | 2012 | The GABPB1 gene A/G polymorphism in Polish rowers | Sport disciplines | included rowers |
| Sgourou | 2012 | Association of genome variations in the renin-angiotensin system with physical performance | Sport disciplines | included swimmers, handball players etc. |
| Yvert | 2012 | Acyl coenzyme A synthetase long-chain 1 (ACSL1) gene polymorphism (rs6552828) and elite endurance athletic status: a replication study | Sport disciplines | included rowers |
| Ben-Zaken | 2013 | Genetic profiles and prediction of the success of young athletes' transition from middle- to long-distance runs: an exploratory study | Control definition | no control group |
| Ben-Zaken | 2013 | Can IGF-I polymorphism affect power and endurance athletic performance? | Sport disciplines | included <5000m runners |
| Djarova | 2013 | Performance enhancing genetic variants, oxygen uptake, heart rate, blood pressure and body mass index of elite high altitude mountaineers | Sport disciplines | included mountaineers |
| Domingo | 2013 | ACE activity and endurance performance during the South African Ironman triathlons | Control definition | no control group |
| Drozdovska | 2013 | The association of gene polymorphisms with athlete status in ukrainians | Sport disciplines | included cross-country skiers and rowers |
| Eynon | 2013 | The rs12594956 polymorphism in the NRF-2 gene is associated with top-level Spanish athlete's performance status | Sport disciplines | included rowers |
| Grealy | 2013 | The genetics of endurance: frequency of the ACTN3 R577X variant in Ironman World Championship athletes | Control definition | no control group |
| Gronek | 2013 | CKM Gene G (Ncoi-) Allele Has a Positive Effect on Maximal Oxygen Uptake in Caucasian Women Practicing Sports Requiring Aerobic and Anaerobic Exercise Metabolism | Sport disciplines | sport disciplines not specified |
| Holdys | 2013 | Genetic variants of uncoupling proteins-2 and -3 in relation to maximal oxygen uptake in different sports | Performance level | included various athlete levels |
| Maciejewska-Karlowska | 2013 | Association between the Pro12Ala polymorphism of the peroxisome proliferator-activated receptor gamma gene and strength athlete status | Sport disciplines | included triathletes, cross-country skiers etc. |
| Mikami | 2013 | Comprehensive analysis of common and rare mitochondrial DNA variants in elite Japanese athletes: a case-control study | Sport disciplines | included <5000m runners, swimmers etc. |
| Mikami | 2013 | Polymorphisms in the control region of mitochondrial DNA associated with elite Japanese athlete status | Sport disciplines | included <5000m runners, swimmers etc. |
| Orysiak | 2013 | The association between ace gene variation and aerobic capacity in winter endurance disciplines | Sport disciplines | included cross-country skiers, biathletes etc. |
| Persi | 2013 | Polymorphisms of alpha-actinin-3 and ciliary neurotrophic factor in national-level Italian athletes | Performance level | VO2max <  71 ml/kg/min,  included biathlon |
| Ruiz | 2013 | ACTN3 genotype in Spanish elite swimmers: no "heterozygous advantage" | Sport disciplines | included rowers |
| Sawczuk | 2013 | Association of the ADRB2 Gly16Arg and Glu27Gln polymorphisms with athlete status | Sport disciplines | included triathletes, swimmers etc. |
| Ulucan | 2013 | Preliminary findings of alpha-actinin-3 gene distribution in elite Turkish wind surfers | Sport disciplines | included windsurfers |
| Wang | 2013 | Association analysis of ACE and ACTN3 in elite Caucasian and East Asian swimmers | Sport disciplines | included swimmers |
| Zarebska | 2013 | Association of rs699 (m235t) polymorphism in the agt gene with power but not endurance athlete status | Sport disciplines | included triathletes, swimmers etc. |
| Abrahams | 2014 | A polymorphism in a functional region of the COL5A1 gene: association with ultraendurance-running performance and joint range of motion | Control definition | no control group |
| Ben-Zaken | 2014 | IGF-I and IGF-I receptor polymorphisms among elite swimmers | Sport disciplines | included swimmers |
| Bertuzzi | 2014 | Is the COL5A1 rs12722 gene polymorphism associated with running economy? | Performance level | included “recreationally active” athletes |
| Eynon | 2014 | ACTN3 R577X polymorphism and team-sport performance: a study involving three European cohorts | Sport disciplines | included rowers, swimmers etc. |
| Fedotovskaya | 2014 | A common polymorphism of the MCT1 gene and athletic performance | Sport disciplines | included <5000m runners, swimmers etc. |
| Ginevičienė | 2014 | AMPD1 rs17602729 is associated with physical performance of sprint and power in elite Lithuanian athletes | Sport disciplines | included skiers, biathletes etc. |
| Grenda | 2014 | Bdkrb2 gene -9/+9 polymorphism and swimming performance | Sport disciplines | included swimmers |
| Grenda | 2014 | Interaction Between ACE I/D and ACTN3 R557X Polymorphisms in Polish Competitive Swimmers | Sport disciplines | included swimmers |
| Gronek | 2014 | Maximal oxygen uptake is associated with allele -202 A of insulin-like growth factor binding protein-3 (IGFBP3) promoter polymorphism and (CA)n tandem repeats of insulin-like growth factor IGF1 in Caucasians from Poland | Performance level | athlete level not defined |
| Gunel | 2014 | Effect of angiotensin I-converting enzyme and α-actinin-3 gene polymorphisms on sport performance | Sport disciplines | sport disciplines not specified |
| Lifanov | 2014 | Influence of the GPX1 gene polymorphism to aerobic capacity and efficiency of glutathione supplementation in athletes | Full text not retrievable | Full text not available, authors have been contacted |
| Maciejewska-Karlowska | 2014 | Genomic haplotype within the Peroxisome Proliferator-Activated Receptor Delta (PPARD) gene is associated with elite athletic status | Sport disciplines | included triathletes, cross-country skiers etc. |
| Malczewska-Lenczowska | 2014 | NO ASSOCIATION BETWEEN tHbmass AND POLYMORPHISMS IN THE HBB GENE IN ENDURANCE ATHLETES | Control definition | no control group |
| Maruszak | 2014 | Mitochondrial DNA variation is associated with elite athletic status in the Polish population | Sport disciplines | included swimmers, rowers etc. |
| Mikami | 2014 | ACTN3 R577X genotype is associated with sprinting in elite Japanese athletes | Sport disciplines | included race walkers and <5000m runners |
| Mustafina | 2014 | AGTR2 gene polymorphism is associated with muscle fibre composition, athletic status and aerobic performance | Performance level | athlete level not defined |
| Orysiak | 2014 | Relationship between ACTN3 R577X polymorphism and maximal power output in elite Polish athletes | Sport disciplines | included swimmers, volleyball players etc. |
| Sawczuk | 2014 | Is gnb3 c825t polymorphism associated with elite status of polish athletes? | Sport disciplines | included triathletes, cross-country skiers etc. |
| Tural | 2014 | PPAR-α and PPARGC1A gene variants have strong effects on aerobic performance of Turkish elite endurance athletes | Performance level | VO2max < 71 ml/kg/min |
| Voisin | 2014 | EPAS1 gene variants are associated with sprint/power athletic performance in two cohorts of European athletes | Sport disciplines | included rowers, skaters etc. |
| Zarebska | 2014 | Association of the MTHFR 1298A>C (rs1801131) polymorphism with speed and strength sports in Russian and Polish athletes | Sport disciplines | included triathletes, cross-country skiers etc. |
| Ahmetov | 2015 | Genome-wide association study identifies three novel genetic markers associated with elite endurance performance | Sport disciplines | included <5000m runners |
| Banting | 2015 | Elite athletes' genetic predisposition for altered risk of complex metabolic traits | Sport disciplines | included rowers, <5000m runners etc. |
| Ben-Zaken | 2015 | Genetic score of power-speed and endurance track and field athletes | Genotype frequency | allele frequency not given |
| Ben-Zaken | 2015 | IGF-I receptor 275124A>C (rs1464430) polymorphism and athletic performance | Sport disciplines | included <5000m runners |
| Bosnyák | 2015 | ACE and ACTN3 genes polymorphisms among female Hungarian athletes in the aspect of sport disciplines | Sport disciplines | included kayakers, rowers, etc. |
| Eider | 2015 | CKM gene polymorphism in Russian and Polish rowers | Sport disciplines | included rowers |
| Filonzi | 2015 | The potential role of myostatin and neurotransmission genes in elite sport performances | Sport disciplines | included soccer players, basketball players etc. |
| Grealy | 2015 | Evaluation of a 7-Gene Genetic Profile for Athletic Endurance Phenotype in Ironman Championship Triathletes | Control definition | no control group |
| Grenda | 2015 | Does the GNB3 C825T Polymorphism Influence Swimming Performance in Competitive Swimmers? | Sport disciplines | included swimmers |
| He | 2015 | PGC-related gene variants and elite endurance athletic status in a Chinese cohort: a functional study | Sport disciplines | included <5000m runners |
| Hermine | 2015 | Eighty percent of French sport winners in Olympic, World and Europeans competitions have mutations in the hemochromatosis HFE gene | Sport disciplines | included nordic skiers and rowers |
| Massidda | 2015 | ACTN3 R577X polymorphism is not associated with team sport athletic status in Italians | Sport disciplines | included triathletes |
| Orysiak | 2015 | Relationship Between ACTN3 R577X Polymorphism and Physical Abilities in Polish Athletes | Sport disciplines | included volleyball players, swimmers etc. |
| Orysiak | 2015 | Overrepresentation of the ACTN3 XX genotype in elite canoe and kayak paddlers | Sport disciplines | included canoeists and kayakers |
| Saunders | 2015 | A variant within the AQP1 3'-untranslated region is associated with running performance, but not weight changes, during an Ironman Triathlon | Control definition | no control group |
| Sawczuk | 2015 | MCT1 A1470T: a novel polymorphism for sprint performance? | Sport disciplines | included cross-country skiers, kayakers etc. |
| Szelid | 2015 | Right Ventricular Adaptation Is Associated with the Glu298Asp Variant of the NOS3 Gene in Elite Athletes | Sport disciplines | included swimmers, rowers etc. |
| van Breda | 2015 | The COMT val(158)met polymorphism in ultra-endurance athletes | Sport disciplines | included triathletes |
| Duvallet | 2016 | Do mutations H63D and C282Y of the gene HFE influence the kinetics of iron metabolism in elite cyclist? | Full text not retrievable | full text not available, authors have been contacted |
| Jin | 2016 | Is there a relationship between PPARD T294C/PPARGC1A Gly482Ser variations and physical endurance performance in the Korean population?. | Sport disciplines | included basketball players, climbers etc. |
| Jones | 2016 | A genetic-based algorithm for personalized resistance training | Sport disciplines | included squashers, swimmers etc. |
| Kikuchi | 2016 | ACTN3 R577X genotype and athletic performance in a large cohort of Japanese athletes | Sport disciplines | included <5000m runners |
| Mägi | 2016 | The Association Analysis between ACE and ACTN3 Genes Polymorphisms and Endurance Capacity in Young Cross-Country Skiers: Longitudinal Study | Sport disciplines | included cross-country skiers |
| Pasqua | 2016 | Influence of ACTN3 R577X polymorphism on ventilatory thresholds related to endurance performance | Performance level | included moderately active individuals |
| Rankinen | 2016 | No Evidence of a Common DNA Variant Profile Specific to World Class Endurance Athletes | Sport disciplines | sport disciplines not specified |
| Sanhueza | 2016 | Association of Anxiety-Related Polymorphisms with Sports Performance in Chilean Long Distance Triathletes: A Pilot Study | Control definition | control group were athletes |
| Tumer | 2016 | Investigation of NRF-1 genotypes and ace gene polymorphismin elite athletes | Sport disciplines | football, basketball, volleyball |
| Voisin | 2016 | ACVR1B rs2854464 Is Associated with Sprint/Power Athletic Status in a Large Cohort of Europeans but Not Brazilians | Sport disciplines | included <5000m runners |
| Yvert | 2016 | Lack of replication of associations between multiple genetic polymorphisms and endurance athlete status in Japanese population | Sport disciplines | included <5000m runners |
| Zmijewski | 2016 | Effect of BDKRB2 Gene -9/+9 Polymorphism on Training Improvements in Competitive Swimmers | Sport disciplines | included swimmers |
| Znazen | 2016 | Genetic advantageous predisposition of angiotensin converting enzyme id polymorphism in Tunisian athletes | Sport disciplines | sport disciplines not specified |
| Ben-Zaken | 2017 | Increased Prevalence of the IL-6-174C Genetic Polymorphism in Long Distance Swimmers | Duplicate study population | See reference 84 |
| Ben-Zaken | 2017 | High prevalence of the IGF2 rs680 GG polymorphism among top-level sprinters and jumpers | Sport disciplines | included <5000m runners |
| Ben-Zaken | 2017 | The combined frequency of IGF and myostatin polymorphism among track & field athletes and swimmers | Genotype frequency | Combined genotype frequency for IGF and MSTN reported |
| Durmic | 2017 | Polymorphisms in ACE and ACTN3 Genes and Blood Pressure Response to Acute Exercise in Elite Male Athletes from Serbia | Sport disciplines | included rowers, football players etc. |
| Guilherme | 2017 | Single Nucleotide Polymorphisms in Carnosinase Genes (CNDP1 and CNDP2) are Associated With Power Athletic Status | Sport disciplines | sport disciplines not specified |
| Li | 2017 | ACTN3 R577X genotype and performance of elite middle-long distance swimmers in China | Sport disciplines | included swimmers |
| Peplonska | 2017 | Genetic variants associated with physical and mental characteristics of the elite athletes in the Polish population | Sport disciplines | included swimmers, <5000m runners etc. |
| Zarebska | 2017 | GSTP1 c.313A>G polymorphism in Russian and Polish athletes | Sport disciplines | included biathletes, triathletes etc. |
| Abe | 2018 | Association between COMT Val158Met polymorphism and competition results of competitive swimmers | Control definition | no control group |
| Amato | 2018 | TOTAL GENETIC SCORE: AN INSTRUMENT TO IMPROVE THE PERFORMANCE IN THE ELITE ATHLETES | Sport disciplines | included basketball players and soccer players |
| Arica | 2018 | -174 G/C polymorphism of interleukin 6 gene is not significantly different in Turkish professional short and long distance runners | Control definition | no control group |
| Eroğlu | 2018 | Prevalence of alpha actinin-3 gene (ACTN3) R577X and angiotensin converting enzyme (ACE) insertion / deletion gene polymorphisms in national and amateur Turkish athletes | Sport disciplines | sport disciplines not specified |
| Gronek | 2018 | Polygenic Study of Endurance-Associated Genetic Markers NOS3 (Glu298Asp), BDKRB2 (-9/+9), UCP2 (Ala55Val), AMPD1 (Gln45Ter) and ACE (I/D) in Polish Male Half Marathoners | Control definition | no control group |
| Guilherme | 2018 | The AGTR2 rs11091046 (A>C) polymorphism and power athletic status in top-level Brazilian athletes | Sport disciplines | included <5000m runners, rowers etc. |
| Guilherme | 2018 | Analysis of sports-relevant polymorphisms in a large Brazilian cohort of top-level athletes | Sport disciplines | included <5000m runners, rowers etc. |
| Karpowicz | 2018 | The relationship between CA repeat polymorphism of the IGF-1 gene and the structure of motor skills in young athletes | Performance level | athlete level not defined |
| Papadimitriou | 2018 | No association between ACTN3 R577X and ACE I/D polymorphisms and endurance running times in 698 Caucasian athletes | Control definition | no control group, included <5000m runners |
| Stebbings | 2018 | TTN genotype is associated with fascicle length and marathon running performance | Control definition | control group was recreationally active |
| Zmijewski | 2018 | The NOS3 G894T (rs1799983) and -786T/C (rs2070744) polymorphisms are associated with elite swimmer status | Sport disciplines | included swimmers |
| Al-Khelaifi | 2019 | Metabolic GWAS of elite athletes reveals novel genetically-influenced metabolites associated with athletic performance | Sport disciplines | included heptathletes, kayakers etc. |
| Chiu | 2019 | Impact of angiotension I converting enzyme gene I/D polymorphism on running performance, lipid, and biochemical parameters in ultra-marathoners | Control definition | no control group |
| Flecha-Velazquez | 2019 | KCNA4 Gene Variant is Auxiliary in Endurance Running Performance Level | Control definition | endurance athletes as control |
| Guilherme | 2019 | The A-allele of the FTO gene rs9939609 polymorphism is associated with decreased proportion of slow oxidative muscle fibers and over-represented in heavier athletes. | Sport disciplines | including swimmers and triathletes |
| Guilherme | 2019 | Association study of SLC6A2 gene Thr99Ile variant (rs1805065) with athletic status in the Brazilian population | Sport disciplines | included <5000m runners, rowers etc. |
| Kiiskilä | 2019 | Analysis of functional variants in mitochondrial DNA of Finnish athletes | Sport disciplines | included <5000m runners |
| Parfenteva | 2019 | Influence of the A/T polymorphism of the FTO gene and sport specializations on the body composition of young Russian athletes | Sport disciplines | sport disciplines not specified |
| Peplonska | 2019 | Association of serotoninergic pathway gene variants with elite athletic status in the Polish population | Sport disciplines | included swimmers, <5000m runners etc. |
| Wuyun | 2019 | The Short Tandem Repeat of the DMT1 Gene as a Molecular Marker of Elite Long-Distance Runners | Rs-number not correct | rs2076732 |
| Al-Khelaifi | 2020 | Genome-Wide Association Study Reveals a Novel Association Between MYBPC3 Gene Polymorphism, Endurance Athlete Status, Aerobic Capacity and Steroid Metabolism. | Sport disciplines | included boxers, kayakers etc. |
| Bosnyák | 2020 | Lack of association between the GNB3 rs5443, HIF1A rs11549465 polymorphisms, physiological and functional characteristics | Performance level | VO2max <71 ml/kg/min |
| Boulygina | 2020 | Whole genome sequencing of elite athletes. | Sport disciplines | included boxers, wrestlers etc. |
| Ghosh | 2020 | Exploring the underlying biology of intrinsic cardiorespiratory fitness through integrative analysis of genomic variants and muscle gene expression profiling. | Performance level | athlete level not defined |
| Guilherme | 2020 | Total genotype score and athletic status: An exploratory cross-sectional study of a Brazilian athlete cohort. | Sport disciplines | included power athletes |
| Guilherme | 2020 | The BDNF-Increasing Allele is Associated With Increased Proportion of Fast-Twitch Muscle Fibers, Handgrip Strength, and Power Athlete Status. | Sport disciplines | included rowers, cross-country skiers etc. |
| Guilherme | 2020 | Are Genome-Wide Association Study Identified Single-Nucleotide Polymorphisms Associated With Sprint Athletic Status? A Replication Study With 3 Different Cohorts. | Sport disciplines | included <5000m runners |
| Harvey | 2020 | Genetic variants associated with exercise performance in both moderately trained and highly trained individuals | Sport disciplines | included triathletes |
| Homma | 2020 | Ciliary Neurotrophic Factor Receptor rs41274853 Polymorphism Is Associated With Weightlifting Performance in Japanese Weightlifters. | Sport disciplines | included weightlifters |
| Iglesia | 2020 | A Potential Endurance Algorithm Prediction in the Field of Sports Performance | Control definition | no control group |
| Kusić | 2020 | Striated muscle-specific serine/threonine-protein kinase beta segregates with high versus low responsiveness to endurance exercise training | Sport disciplines | included <5000m runners, swimmers etc. |
| Mavlyanov | 2020 | RELATIVE FEATURES OF THE PPARA (rs4253778), PPARGC1A(rs8192678) AND PPARG2(rs1801282) POLYMORPHISMS GENES IN ATHLETES ENGAGED IN CYCLIC TYPES OF SPORTS | Sport disciplines | included rowers, no control group |
| Moreland | 2020 | Polygenic Profile of Elite Strength Athletes. | Sport disciplines | included weightlifters,  powerlifters |
| Nursal | 2020 | A case-control study investigating the effect of MTHFR C677T variant on performance of elite athletes. | Full text not retrievable | full text not available, authors have been contacted |
| Neto | 2020 | Interaction Between ACTN3 (R577X), ACE (I/D), and BDKRB2 (-9/+9) Polymorphisms and Endurance Phenotypes in Brazilian Long-Distance Swimmers. | Sport disciplines | included swimmers |
| Pickering | 2020 | Title: Can genetic testing predict talent? A case study of five elite athletes | Case study | case study |
| Semenova | 2020 | The association of HFE gene H63D polymorphism with endurance athlete status and aerobic capacity: novel findings and a meta-analysis | Sport disciplines | included rowers, kayakers etc. |
| Wojciechowicz | 2020 | Are KIF6 and APOE polymorphisms associated with power and endurance athletes? | Sport disciplines | included triathletes, cross-country skiers etc. |
| Yvert | 2020 | PPARGC1A rs8192678 and NRF1 rs6949152 Polymorphisms Are Associated with Muscle Fiber Composition in Women. | Performance level | athlete level not defined |
| Ben-Zaken | 2021 | Genetic Basis for the Dominance of Israeli Long-Distance Runners of Ethiopian Origin | Sport disciplines | included <5000m runners |
| Ben-Zaken | 2021 | Insulin-like Growth Factor Axis Genetic Score and Sports Excellence | Genotype frequency | genetic score of 6 polymorphisms together presented |
| Bosnyák | 2021 | Lack of association between the GNB3 rs5443, HIF1A rs11549465 polymorphisms, physiological and functional characteristics. | Sport disciplines | included modern pentathlon, rowers etc. |
| Fichna | 2021 | Rare Variant in the SLC6A2 Encoding a Norepinephrine Transporter Is Associated with Elite Athletic Performance in the Polish Population | Sport disciplines | endurance sports defined as > 8 min |
| Ginevičienė | 2021 | Variants in the Myostatin Gene and Physical Performance Phenotype of Elite Athletes | Sport disciplines | included swimmers, rowers, etc. |
| Guilherme | 2021 | The MCT1 gene Glu490Asp polymorphism (rs1049434) is associated with endurance athlete status, lower blood lactate accumulation and higher maximum oxygen uptake | Sport disciplines | included rowing, swimming, etc |
| Gutiérrez-Hellín | 2021 | Effect of ACTN3 R577X Genotype on Injury Epidemiology in Elite Endurance Runners | Control definition | no control group,  800 m runners included |
| Hall | 2021 | Genetic Polymorphisms Related to VO2max Adaptation Are Associated With Elite Rugby Union Status and Competitive Marathon Performance | Performance level | marathon time of ≤ 3 hours |
| Kobayashi | 2021 | Association Between Low Bone Mineral Density Risk Factors and Estrogen Receptor α Gene Polymorphisms in Japanese Female Athletes. | Performance level | athletes defined as university students |
| Peplonska | 2021 | Common Myelin Regulatory Factor Gene Variants Predisposing to Excellence in Sports. | Sport disciplines | endurance defined as > 8 min |
| Yang | 2021 | Prediction and Identification of Power Performance Using Polygenic Models of Three Single-Nucleotide Polymorphisms in Chinese Elite Athletes | Control definition | power athletes were used as control group |

# **Supplementary 7.** Key characteristics of all included studies (n=43), sorted by year of publication.

| Year | First author | Rs-number/ marker  (Reported in article) | Genotypes | Gene (dbSNP)  (Reported in article) | N athletes, cases/total  N controls,  cases/total | Odds ratio  [95% confidence interval] | Sport  discipline | Performance level | Sex | Country  of study population | Risk of bias | Source | Comments |
| --- | --- | --- | --- | --- | --- | --- | --- | --- | --- | --- | --- | --- | --- |
| 1999 | Myerson^61^ | ***ACE* I/D^†^** | **II** | ***ACE*** | **14/34**  **457/1906** | **2.22**  **[1.11; 4.43]** | Running | International | Male,  Female | Britain | 8 | Medline |  |
| 2000 | Alvarez^62^ | *ACE* I/D**^†^** | II | *ACE* | 12/45  62/400 | 1.98  [0.97; 4.05] | Running,  Cycling | National, International | Male | Spain | 8 | Medline | No ethnicity for cyclers |
| 2001 | Nazarov^63^ | *ACE* I/D**^†^** | II | *ACE* | 2/10  105/449 | 0.82  [0.17; 3.92] | Running | National | Male,  Female | Russia | 7 | Embase | No ethnicity for runners |
| 2002 | Scanavini^64^ | *ACE* I/D**^†^** | II | *ACE* | 5/17  19/152 | 2.92  [0.92; 9.20] | Running,  Cycling | International | Male,  Female | Italy | 5 | Embase | Letter to the editor |
| 2006 | Lucia^74^ | rs1815739**^†^** | **TT** | *ACTN3* | 14/50  22/123 | 1.79  [0.83; 3.86] | Cycling | National | Male | Spain | 8 | Embase |  |
| 2007 | Amir^65^ | *ACE* I/D**^†^** | II | *ACE* | 7/79  26/247 | 0.83  [0.34; 1.98] | Running | National | Male,  Female | Israel | 9 | Embase |  |
| 2009 | Eynon^75^ | **rs1815739^†^** | **TT** | ***ACTN3*** | **24/74**  **42/240** | **2.26**  **[1.25; 4.08]** | Running | National, International | Male,  Female | Israel | 8 | Embase | Duplicate^76^ |
| 2009 | Eynon^128^ | rs2016520 | TT | *PPARD* | 29/74  93/240 | 1.02  [0.60; 1.74] | Running | National, International | Male,  Female | Israel | 9 | Embase | *PPARGC1A* excluded^79^,  Duplicate^56^ |
| 2009 | Eynon^129^ | **rs7181866** | **GG+AG** | ***GABPB1***  ***(NRF2)*** | **9/74**  **5/240** | **6.51**  **[2.1; 20.09]** | Running | National, International | Male,  Female | Israel | 9 | Medline | DM,  Duplicate^56^ |
| 2009 | Eynon^87^ | **rs5443** | **TT** | ***GNB3*** | **14/74**  **20/234** | **2.50**  **[1.19; 5.24]** | Running | National, International | Male,  Female | Israel | 8 | Medline | Duplicate^30^ |
| 2009 | Gomez-Gallego^66^ | *ACE* I/D***^†^***  rs1815739 | II  TT | *ACE*  *ACTN3* | 13/46  11/46  11/46  8/46 | 1.25  [0.49; 3.19]  1.49  [0.54; 4.14] | Cycling | International | Male | Spain | 7 | Medline |  |
| 2009 | Gomez-Gallego^127^ | rs699^‡^ | CC | *AGT* | 16/100  19/119 | 1.00  [0.49; 2.07] | Running,  Cycling | International | Male | Spain | 8 | Medline | Duplicate^69‡^ |
| 2009 | Ruiz^67^ | *ACE* I/D**^†^**  rs1815739  **rs17602729**  rs8111989  rs1799945  rs1805086  **rs8192678** | II  TT  **CC**  TT  GG+CG  GG+AG  **GG** | *ACE*  *ACTN3*  ***AMPD1***  *CKMM*  *(Ncol)*  *HFE*  *MSTN*  *(GDF8)*  ***PPARGC1A*** | 14/46  23/123  11/46  22/123  **44/46**  **101/123**  21/46  43/123  22/46  41/123  6/46  12/123  **26/46**  **47/123** | 1.90  [0.88; 4.13]  1.44  [0.64; 3.27]  **4.79**  **[1.08;21.27]**  1.56  [0.79; 3.11]  1.83  [0.92; 3.65]  1.39  [0.49; 3.94]  **2.10**  **[1.06; 4.18]** | Running,  Cycling | National, International | Male | Spain | 5 | Embase | DM  DM |
| 2010 | Eynon^79^ | **rs8192678**  **rs4253778** | **GG**  **GG** | ***PPARGC1A***  ***PPARA*** | **37/74**  **79/240**  **7/74**  **10/240** | **2.04**  **[1.2; 3.46]**  **2.40**  **[0.88; 6.56]** | Running | National, International | Male,  Female | Israel | 9 | Medline | Duplicate^56,128^  Duplicate^56^ |
| 2010 | Eynon^130^ | **rs12594956**  **rs8031031** | **AA**  **TT+CT** | ***GABPB1***  ***(NRF2)***  ***GABPB1***  ***(NRF2)*** | **43/74**  **102/240**  **8/74**  **9/240** | **1.88**  **[1.11; 3.18]**  **3.11**  **[1.56; 8.38]** | Running | National, International | Male,  Female | Israel | 8 | Medline | Duplicate^56^  DM, Duplicate^56^ |
| 2010 | Eynon^76^ | rs11549465 | TT+CT | *HIF1A* | 21/74  67/240 | 1.02  [0.57; 1.82] | Running | National, International | Male,  Female | Israel | 8 | Embase | DM, *ACTN3* excluded^75^ |
| 2010 | Muniesa^68^ | *ACE* I/D**^†^**  rs1815739  rs8192678  rs17602729  rs8111989  rs1805086 | II  TT  GG  CC  TT  GG+AG | *ACE*  *ACTN3*  *PPARGC1A*  *AMPD1*  *CKMM*  *(Ncol)*  *MSTN*  *(GDF8)* | 31/102  24/123  22/102  22/123  49/102  47/123  93/102  101/123  45/102  43/123  12/102  12/123 | 1.80  [0.97; 3.33]  1.26  [0.65; 2.44]  1.49  [0.88; 2.55]  2.25  [0.99; 5.14]  1.47  [0.86; 2.52]  1.23  [0.53;2.88] | Running,  Cycling | International | Male | Spain | 7 | Medline | DM |
| 2010 | Ruiz^69^ | *ACE* I/D  (rs1799752)  **rs1815739**    rs1805086 | II  **TT**  GG+AG | *ACE*  ***ACTN3***  *MSTN*  *(GDF8)* | 44/100  44/100  **29/100**  **13/100**  10/100  8/100 | 1.0  [0.57; 1.75]  **2.73**  **[1.32; 5.65]**  1.28  [0.48; 3.38] | Running,  Cycling | National, International | Male | Spain | 5 | Embase | *AGT*^127^, *IL6*^85^,  *NOS3*^89^ excluded  DM |
| 2010 | Ruiz^85^ | rs1800795 | CC | *IL6* | 15/100  12/100 | 1.29  [0.57; 2.93] | Running,  Cycling | National, International | Male | Spain | 7 | Medline | Duplicate^69^ |
| 2010 | Tobina^70^ | *ACE* I/D**^†^** | II | *ACE* | 19/37  155/335 | 1.23  [0.62; 2.42] | Running | National, International | Male | Japan | 7 | Embase |  |
| 2011 | Ash^71^ | *ACE* I/D**^†^**  rs4363 | II  AA | *ACE*  *ACE* | 12/76  36/408  10/76  50/410 | 1.94  [0.96; 3.92]  1.09  [0.53; 2.26] | Running | International | Male,  Female | Ethiopia | 8 | Embase |  |
| 2011 | Eynon^31^ | rs1800795 | CC | *IL6* | 2/74  5/205 | 1.11  [0.21; 5.85] | Running | National, International | Male,  Female | Israel,  Spain | 9 | Medline | Spanish cohort excluded,  Duplicate^69^ |
| 2011 | Eynon^30^ | *BDKRB2 -9/+9*  (rs5810761) | +9/+9 | *BDKRB2* | 22/74  63/240 | 1.19  [0.67; 2.11] | Running | National, International | Male,  Female | Israel | 9 | Medline | *GNB3* excluded^87^ |
| 2011 | Ruiz^88^ | rs5443 | TT | *GNB3* | 9/100  13/100 | 0.66  [0.27; 1.63] | Running,  Cycling | International | Male | Israel  Spain | 8 | Medline | Israel cohort excluded^30^ |
| Year | **First author** | **Rs-number/ marker**  **(Reported in article)** | **Effect**  **allele** | **Gene**  **(Reported in article)** | **N athletes, cases/total**  **N controls,**  **cases/total** | **Odds ratio**  **[95% confidence interval]** | **Sport**  **discipline** | **Performance level** | **Sex** | **Country**  **of study population** | **Risk of bias** | **Source** | **Comments** |
| 2011 | Santiago^86^ | rs1801253  rs1042713  rs1042714  **rs4994** | CC  GG  CC  **CC+CT** | *ADRB1*  *ADRB2*  *ADRB2*  ***ADRB3*** | 48/100  43/100  36/100  34/100  50/100  48/100  **27/100**  **8/100** | 1.22  [0.70; 2.14]  1.09  [0.61; 1.95]  1.08  [0.62; 1.89]  **4.25**  **[1.82; 9.92]** | Running,  Cycling | International | Male | Spain | 6 | Medline | DM |
| 2012 | Eynon^89^ | rs2070744 | TT | *NOS3* | 33/100  34/100 | 0.96  [0.53; 1.72] | Running,  Cycling | International | Male | Spain | 8 | Medline | Duplicate^57,69^ |
| 2012 | Maciejewska^80^ | rs8192678 | GG | *PPARGC1A* | 9/14  280/684 | 2.60  [0.86; 7.83] | Cycling | International | Male,  Female | Poland  Russia | 7 | Embase | Russian cohort excluded |
| 2013 | Ben-Zaken^131^ | **rs4880**  **(rs1799725)** | **CC** | ***SOD2***  ***(MnSOD)*** | **29/121**  **33/240** | **1.98**  **[1.13; 3.45]** | Running | National, International | Male,  Female | Israel | 8 | Medline |  |
| 2013 | Eynon^132^ | rs9939609 | AA | *FTO* | 5/49  5/60 | 1.25  [0.34; 4.59] | Running | International | Male | Spain  Russia  Poland | 7 | Medline | Russian and Polish cohort excluded |
| 2013 | Sawzcuk^82^ | *BDKRB* -9/+9  (rs5810761) | +9/+9 | *BDKRB2* | 4/14  297/684 | 0.99  [0.31; 3.19] | Cycling | National, International | Male,  Female | Poland  Russia | 8 | Medline | Russian cohort excluded |
| 2014 | Shahmoradi^72^ | *ACE* I/D**^†^** | II | *ACE* | 6/37  27/163 | 0.97  [0.37; 2.56] | Cycling | National, International | Male | Iran | 8 | Medline |  |
| 2014 | Xia^27^ | **rs5418** | **AA** | ***SLC2A4*** | **63/102**  **94/206** | **1.92**  **[1.19; 3.12]** | Running | International | Male,  Female | China | 7 | Medline |  |
| 2015 | Ben-Zaken^77^ | **rs1815739** | **TT** | ***ACTN3*** | **23/65**  **40/217** | **2.42**  **[1.31; 4.47]** | Running | National, International | Male,  Female | Israel | 7 | Medline |  |
| 2015 | Ben-Zaken^133^ | rs1049434 | AA | *SLC16A1*  *(MCT1)* | 33/61  56/128 | 1.52  [0.82; 2.80] | Running | National, International | Male,  Female | Israel | 8 | Medline |  |
| 2015 | Ben-Zaken^81^ | rs1805086 | GG+AG | *MSTN*  *(GDF8)* | 18/113  12/118 | 1.67  [0.77; 3.66] | Running | National, International | Male,  Female | Israel | 7 | Medline | DM |
| 2016 | Malczewska^134^ | Intron 2, +16 C/G***^†^***  -551 C/T***^†^*** | CC  CC | *HBB*  *HBB* | 6/89  3/109  18/89  19/109 | 2.55  [0.62;10.52]  1.20  [0.59; 2.46] | Cycling | National | Male,  Female | Poland | 6 | Medline |  |
| 2017 | Lin^126^ | **rs1472955**  rs4672568  **rs11904281** | **AA**  AA+AG  **GG** | ***MYL1***  *MYL1*  ***MYL1*** | **15/31**  **62/206**  23/31  148/206  **16/31**  **68/206** | **2.18**  **[1.01; 4.68]**  1.13  [0.48; 2.66]  **2.16**  **[1.01; 4.64]** | Running | National, International | Male,  Female | China | 8 | Medline | DM |
| 2017 | Yang^78^ | rs1815739 | TT | *ACTN3* | 14/44  17/50 | 0.91  [0.38; 2.15] | Running | National, International | Male,  Female | China | 9 | Embase |  |
| 2019 | Delgado^29^ | **rs3892097**  Functional(+)/  Null(-)***^†^***  **rs1695**  **Functional(+)/**  **Null(-)*^†^*** | GG  -  **AA**  **+** | ***CYP2D6***  *GSTM1*  ***GSTP1***  ***(GSTP)***  ***GSTT*** | **105/123**  **76/122**  87/123  77/122  **77/123**  **61/122**  **89/123**  **70/122** | **3.53**  **[1.9; 6.56]**  1.41  [0.83; 2.41]  **1.67**  **[1.01; 2.79]**  **1.94**  **[1.14; 3.32]** | Running,  Cycling | International | Male | Spain | 6 | Medline |  |
| 2019 | Flück^73^ | *ACE* I/D  (rs1799752)  rs1815739  rs2104772 | II  TT  AA | *ACE*  *ACTN3*  *TNC* | 8/30  9/63  10/30  25/63  6/30  12/63 | 2.18  [0.75; 6.38]  0.76  [0.31; 1.89]  1.06  [0.36; 3.17] | Running,  Cycling | International | Male,  Female | Switzer  land | 8 | Embase |  |
| 2020 | Ben-Zaken^84^ | rs1800795  rs2854744 | CC  AA | *IL6*  *IGFBP3* | 2/63  2/64  19/63  13/64 | 1.02  [0.14; 7.45]  1.69  [0.75; 3.82] | Running | National, International | Male,  Female | Israel | 8 | Medline | Duplicate^32^ |
| 2020 | Delgado^28^ | **rs17602729**  rs8192678  **rs1799945**  rs1800562 | **CC**  GG  **GG+CG**  GG | ***AMPD1***  *PPARGC1A*  ***HFE***  *HFE* | **98/123**  **81/122**  77/123  65/122  **74/123**  **34/122**  114/123  113/122 | **1.98**  **[1.11; 3.54]**  1.47  [0.88; 2.44]  **4.19**  **[2.45; 7.16]**  1.01  [0.39; 2.63] | Running,  Cycling | International | Male | Spain | 5 | Embase | DM |
| 2021 | Delgado^83^ | ***ACE* I/D**  (rs4340)  **rs2070744**  rs1799983  rs1800544  rs553668  rs1042713  rs1042714  ***BDKRB2***  ***-9/+9^†^*** | **II**  **TT**  GG  CC  GG  GG  CC  **+9/+9** | ***ACE***  ***NOS3***  *NOS3*  *ADRA2A*  *ADRA2A*  *ADRB2*  *ADRB2*  ***BDKRB2*** | **82/123**  **44/122**  **62/123**  **39/122**  53/123  47/122  7/123  3/122  94/123  87/122  44/123  46/122  31/123  21/122  **43/123**  **27/122** | **0.43**  **[0.16; 1.17]**  **2.16**  **[1.29; 3.64]**  1.21  [0.73; 2.01]  2.39  [0.60; 9.48]  1.30  [0.74; 2.31]  0.92  [0.55; 1.55**]**  1.62  [0.87; 3.02]  **1.89**  **[1.07; 3.33]** | Running,  Cycling | International | Male | Spain | 5 | Embase |  |

DM=dominant inheritance model assumed.

Statistically significant results are displayed in bold.

**†** Rs-number not mentioned in original article.

‡ Gomez-Gallego et al., (2009)^127^ and Ruiz et al., (2010)^69^ report contrary results for genotype distribution of identical athletes (rs699, *AGT*). Ruiz et al., (2010)^69^ reported a significantly higher CC genotype of athletes compared to controls (CC vs. TC+TT) (odds ratio [95%CI]: 2.70 [1.38-5.32]).

# **Supplementary 8**. Allele and genotype frequencies.

| Year | First author | Rs-number/ marker  (Reported in article) | Genotypes | Gene (dbSNP)  (Reported in article) | Genotypes / Alleles | Frequencies  Athletes | | | | | Frequencies  Controls | | | | | Frequencies  World population | |
| --- | --- | --- | --- | --- | --- | --- | --- | --- | --- | --- | --- | --- | --- | --- | --- | --- | --- |
|  |  |  |  |  |  | Genotype (%) | | | Allele (%) | | Genotype (%) | | | Allele (%) | | Allele (%) | |
| 1999 | Myerson^61^ | *ACE* I/D^†^ | II | *ACE* | II ID DD / I D | 0.41 | 0.41 | 0.18 | 0.62 | 0.38 | 0.24 | 0.50 | 0.26 | 0.49 | 0.51 | - | - |
| 2000 | Alvarez^62^ | *ACE* I/D^†^ | II | *ACE* | II ID DD / I D | 0.27 | 0.55 | 0.18 | 0.54 | 0.46 | 0.15 | 0.46 | 0.39 | 0.38 | 0.62 | - | - |
| 2001 | Nazarov^63^ | *ACE* I/D^†^ | II | *ACE* | II ID DD / I D | 0.20 | 0.50 | 0.30 | 0.45 | 0.55 | 0.23 | 0.53 | 0.24 | 0.50 | 0.50 | - | - |
| 2002 | Scanavini^64^ | *ACE* I/D^†^ | II | *ACE* | II ID DD / I D | 0.30 | 0.35 | 0.35 | 0.48 | 0.52 | 0.13 | 0.43 | 0.44 | 0.35 | 0.65 | - | - |
| 2006 | Lucia^74^ | rs1815739^†^ | TT | *ACTN3* | TT CT CC / T C | 0.28 | 0.46 | 0.26 | 0.51 | 0.49 | 0.18 | 0.53 | 0.29 | 0.45 | 0.55 | 0.43 | 0.57 |
| 2007 | Amir^65^ | *ACE* I/D^†^ | II | *ACE* | II ID DD / I D | 0.09 | 0.29 | 0.62 | 0.23 | 0.77 | 0.10 | 0.47 | 0.43 | 0.34 | 0.66 | - | - |
| 2009 | Eynon^75^ | rs1815739^†^ | TT | *ACTN3* | TT CT CC / T C | 0.32 | 0.49 | 0.19 | 0.57 | 0.43 | 0.18 | 0.62 | 0.20 | 0.49 | 0.51 | 0.43 | 0.57 |
| 2009 | Eynon^128^ | rs2016520 | TT^‡^ | *PPARD* | CC CT TT / C T | 0.11 | 0.50 | 0.39 | 0.36 | 0.64 | 0.12 | 0.49 | 0.39 | 0.36 | 0.64 | 0.20 | 0.80 |
| 2009 | Eynon^129^ | rs7181866 | GG+AG | *GABPB1*  *(NRF2)* | GG AG AA / G A | 0 | 0.12 | 0.88 | 0.06 | 0.94 | 0 | 0.02 | 0.98 | 0.01 | 0.99 | 0.04 | 0.96 |
| 2009 | Eynon^87^ | rs5443 | TT | *GNB3* | TT TC CC / T C | 0.19 | 0.47 | 0.34 | 0.43 | 0.57 | 0.09 | 0.53 | 0.38 | 0.35 | 0.65 | 0.35 | 0.65 |
| 2009 | Gomez-  Gallego^66^ | *ACE* I/D*^†^*  rs1815739 | II  TT | *ACE*  *ACTN3* | II ID DD / I D  TT CT CC / T C | 0.28  0.24 | 0.31  NR | 0.41  NR | 0.44  - | 0.56  - | 0.24  0.17 | 0.46  NR | 0.30  NR | 0.47  - | 0.53  - | -  0.43 | -  0.57 |
| 2009 | Gomez-Gallego^127^ | rs699^‡^ | CC | *AGT* | CC CT TT / C T | 0.16 | 0.50 | 0.34 | 0.41 | 0.59 | 0.16 | 0.50 | 0.34 | 0.41 | 0.59 | 0.46 | 0. 54 |
| 2009 | Ruiz^67^ | *ACE* I/D^†^  rs1815739  rs17602729  rs8111989  rs1799945  rs1805086  rs8192678 | II  TT  CC  TT  GG+CG  GG+AG^‡^  GG | *ACE*  *ACTN3*  *AMPD1*  *CKMM*  *(Ncol)*  *HFE*  *MSTN*  *(GDF8)*  *PPARGC1A* | II ID DD / I D  TT CT CC / T C  TT CT CC / T C  CC CT TT / T C  GG CG CC / G C  GG AG AA / G A  AA AG GG / A G | 0.30  0.24  0  0.11  0.07  0  0.04 | 0.22  0.54  0.04  0.43  0.42  0.13  0.39 | 0.48  0.22  0.96  0.46  0.51  0.87  0.57 | 0.41  0.51  0.02  0.33  0.28  0.06  0.24 | 0.59  0.49  0.98  0.67  0.72  0.94  0.76 | 0.19  0.28  0.01  0.12  0.09  0  0.11 | 0.46  0.54  0.17  0.53  0.24  0.10  0.51 | 0.35  0.18  0.82  0.35  0.67  0.90  0.38 | 0.42  0.55  0.10  0.39  0.21  0.05  0.37 | 0.58  0.45  0.90  0.61  0.79  0.95  0.63 | -  0.43  0.12  0.33  0.14  0.03  0.33 | -  0.57  0.88  0.67  0.86  0.97  0.67 |
| 2010 | Eynon^79^ | rs8192678  rs4253778 | GG  GG | *PPARGC1A*  *PPARA* | AA AG GG / A G  GG GC CC / G C | 0  0.10 | 0.50  0.28 | 0.50  0.62 | 0.25  0.24 | 0.75  0.76 | 0.18  0.04 | 0.49  0.28 | 0.33  0.68 | 0.43  0.18 | 0.57  0.82 | 0.33  0.11 | 0.67  0.89 |
| 2010 | Eynon^130^ | rs12594956  rs8031031 | AA  TT+CT | *GABPB1*  *(NRF2)*  *GABPB1*  *(NRF2)* | CC AC AA / C A  TT CT CC / T C | 0.05  0 | 0.37  0.11 | 0.58  0.89 | 0.24  0.05 | 0.76  0.95 | 0.11  0 | 0.46  0.04 | 0.43  0.96 | 0.34  0.02 | 0.66  0.98 | 0.39  0.06 | 0.61  0.94 |
| 2010 | Eynon^76^ | rs11549465 | TT+CT | *HIF1A* | TT CT CC / T C | 0 | 0.28 | 0.72 | 0.14 | 0.86 | 0.02 | 0.26 | 0.72 | 0.15 | 0.85 | 0.11 | 0.89 |
| 2010 | Muniesa^68^ | *ACE* I/D^†^  rs1815739  rs8192678  rs17602729  rs8111989  rs1805086 | II  TT  GG  CC  TT  GG+AG | *ACE*  *ACTN3*  *PPARGC1A*  *AMPD1*  *CKMM*  *(Ncol)*  *MSTN*  *(GDF8)* | II ID DD / I D  TT CT CC / T C  AA AG GG / A G  TT CT CC / T C  CC CT TT / T C  GG AG AA / G A | 0.30  0.22  0.16  0  0.06  0.02 | 0.28  0.52  0.36  0.09  0.50  0.10 | 0.42  0.26  0.48  0.91  0.44  0.88 | 0.44  0.48  0.34  0.05  0.31  0.07 | 0.56  0.52  0.66  0.95  0.69  0.93 | 0.20  0.18  0.11  0.01  0.12  0 | 0.45  0.54  0.51  0.17  0.53  0.10 | 0.35  0.28  0.38  0.82  0.35  0.90 | 0.43  0.45  0.37  0.10  0.39  0.05 | 0.57  0.55  0.63  0.90  0.61  0.95 | -  0.43  0.33  0.12  0.33  0.03 | -  0.57  0.67  0.88  0.67  0.97 |
| 2010 | Ruiz^69^ | *ACE* I/D  (rs1799752)  rs1815739    rs1805086 | II  TT  GG+AG | *ACE*  *ACTN3*  *MSTN*  *(GDF8)* | II ID DD / I D  TT CT CC / T C  GG AG AA / G A | 0.44  0.29  0 | 0.36  0.46  0.10 | 0.20  0.25  0.90 | 0.62  0.52  0.05 | 0.38  0.48  0.95 | 0.44  0.13  0 | 0.43  0.58  0.08 | 0.13  0.29  0.92 | 0.66  0.42  0.04 | 0.34  0.58  0.96 | -  0.43  0.03 | -  0.57  0.97 |
| 2010 | Ruiz^85^ | rs1800795 | CC | *IL6* | CC CG GG / C G | 0.15 | 0.43 | 0.42 | 0.37 | 0.63 | 0.12 | 0.46 | 0.42 | 0.35 | 0.65 | 0.36 | 0.64 |
| 2010 | Tobina^70^ | *ACE* I/D^†^ | II | *ACE* | II ID DD / I D | 0.51 | 0.27 | 0.22 | 0.65 | 0.35 | 0.46 | 0.44 | 0.10 | 0.68 | 0.32 | - | - |
| 2011 | Ash^71^ | *ACE* I/D^†^  rs4363 | II  AA | *ACE*  *ACE* | II ID DD / I D  AA AG GG / A G | 0.16  0.13 | 0.45  0.45 | 0.39  0.42 | 0.39  0.35 | 0.61  0.65 | 0.09  0.12 | 0.46  0.46 | 0.45  0.42 | 0.32  0.35 | 0.68  0.65 | -  0.48 | -  0.52 |
| 2011 | Eynon^31^ | rs1800795 | CC | *IL6* | CC CG GG / C G | 0.03 | 0.26 | 0.71 | 0.16 | 0.84 | 0.02 | 0.26 | 0.72 | 0.15 | 0.85 | 0.36 | 064 |
| 2011 | Eynon^30^ | *BDKRB2 -9/+9*  (rs5810761) | +9/+9^‡^ | *BDKRB2* | -9/-9 +9/-9 +9/+9 | 0.16 | 0.54 | 0.30 | 0.43 | 0.57 | 0.18 | 0.56 | 0.26 | 0.46 | 0.54 | - | - |
| 2011 | Ruiz^88^ | rs5443 | TT | *GNB3* | TT TC CC / T C | 0.09 | 0.59 | 0.32 | 0.39 | 0.61 | 0.13 | 0.45 | 0.42 | 0.35 | 0.65 | 0.33 | 0.67 |
| 2011 | Santiago^86^ | rs1801253  rs1042713  rs1042714  rs4994 | CC  GG  CC  CC+CT | *ADRB1*  *ADRB2*  *ADRB2*  *ADRB3* | GG CG CC / GC  AA AG GG / AG  GG GC CC / GC  CC TC TT / CT | 0.05  0.22  0.15  0 | 0.47  0.42  0.35  0.27 | 0.48  0.36  0.50  0.73 | 0.28  0.43  0.33  0.14 | 0.72  0.57  0.67  0.86 | 0.10  0.26  0.14  0 | 0.47  0.40  0.38  0.08 | 0.43  0.34  0.48  0.92 | 0.23  0.46  0.33  0.04 | 0.77  0.54  0.67  0.96 | 0.29  0.39  0.20  0.08 | 0.71  0.61  0.80  0.92 |
| 2012 | Eynon^89^ | rs2070744 | TT^‡^ | *NOS3* | CC TC TT / C T | 0.27 | 0.40 | 0.33 | 0.47 | 0.53 | 0.21 | 0.45 | 0.34 | 0.43 | 0.57 | 0.35 | 0.65 |
| 2012 | Maciejewska^80^ | rs8192678 | GG | *PPARGC1A* | AA AG GG / A G | 0 | 0.36 | 0.64 | 0.18 | 0.82 | 0.13 | 0.46 | 0.41 | 0.36 | 0.64 | 0.33 | 0.67 |
| 2013 | Ben-Zaken^131^ | rs4880  (rs1799725) | CC | *SOD2*  *(MnSOD)* | CC CT TT / C T | 0.24 | 0.45 | 0.31 | 0.46 | 0.54 | 0.14 | 0.31 | 0.55 | 0.30 | 0.70 | 0.49 | 0.51 |
| 2013 | Eynon^132^ | rs9939609 | AA^‡^ | *FTO* | AA AT TT / A T | 0.11 | 0.28 | 0.61 | 0.25 | 0.75 | 0.80 | 0.12 | 0.08 | 0.86 | 0.14 | 0.40 | 0.60 |
| 2013 | Sawzcuk^82^ | *BDKRB* -9/+9  (rs5810761) | +9/+9 | *BDKRB2* | -9/-9 +9/-9 +9/+9 | 0.29 | 0.57 | 0.14 | 0.58 | 0.42 | 0.29 | 0.51 | 0.20 | 0.55 | 0.45 | - | - |
| 2014 | Shahmoradi^72^ | *ACE* I/D^†^ | II | *ACE* | II ID DD / I D | 0.16 | 0.41 | 0.43 | 0.36 | 0.64 | 0.16 | 0.45 | 0.39 | 0.39 | 0.61 | - | - |
| 2014 | Xia^27^ | rs5418 | AA | *SLC2A4* | GG GA AA / G A | 0.13 | 0.25 | 0.62 | 0.25 | 0.75 | 0.14 | 0.41 | 0.45 | 0.35 | 0.65 | 0.43 | 0.57 |
| 2015 | Ben-Zaken^77^ | rs1815739 | TT | *ACTN3* | TT CT CC / T C | 0.35 | 0.45 | 0.20 | 0.58 | 0.42 | 0.18 | 0.60 | 0.22 | 0.48 | 0.52 | 0.43 | 0.57 |
| 2015 | Ben-Zaken^133^ | rs1049434 | AA | *SLC16A1*  *(MCT1)* | AA AT TT / A T | 0.54 | 0.38 | 0.08 | 0.73 | 0.27 | 0.44 | 0.44 | 0.12 | 0.66 | 0.34 | 0.41 | 0.59 |
| 2015 | Ben-Zaken^81^ | rs1805086 | GG+AG | *MSTN*  *(GDF8)* | GG AG AA / G A | 0.01 | 0.15 | 0.84 | 0.09 | 0.91 | 0.02 | 0.08 | 0.90 | 0.06 | 0.94 | 0.03 | 0.97 |
| 2016 | Malczewska^134^ | Intron 2, +16 C/G*^†^*  -551 C/T*^†^* | CC  CC | *HBB*  *HBB* | CC CG GG / C G  CC CT TT / C T | 0.06  0.20 | 0.34  0.52 | 0.60  0.28 | 0.23  0.46 | 0.77  0.54 | 0.02  0.17 | 0.37  0.57 | 0.61  0.26 | 0.21  0.45 | 0.79  0.55 | -  - | -  - |
| 2017 | Lin^126^ | rs1472955  rs4672568  rs11904281 | AA  AA+AG  GG | *MYL1*  *MYL1*  *MYL1* | GG GA AA / G A  AA AG GG / A G  CC CG GG / C G | 0  0  0 | 0.52  0.74  0.48 | 0.48  0.26  0.52 | 0.26  0.37  0.24 | 0.74  0.63  0.76 | 0.16  0.16  0.15 | 0.54  0.56  0.52 | 0.30  0.28  0.33 | 0.43  0.44  0.41 | 0.57  0.56  0.59 | 0.40  0.26  0.39 | 0.60  0.74  0.61 |
| 2017 | Yang^78^ | rs1815739 | TT | *ACTN3* | TT CT CC / T C | 0.32 | 0.36 | 0.32 | 0.50 | 0.50 | 0.34 | 0.40 | 0.26 | 0.54 | 0.46 | 0.43 | 0.57 |
| 2019 | Delgado^29^ | rs3892097  Functional(+)/  Null(-)*^†^*  rs1695  Functional(+)/  Null(-)*^†^* | GG  -^‡^  AA  + | *CYP2D6*  *GSTM1*  *GSTP1*  *(GSTP)*  *GSTT* | AA GA GG / A G  (+) (-)  GG AG AA / G A  (+) (-) | 0.01  0.29  0.06  0.72 | 0.14  0.31 | 0.85  0.71  0.63  0.28 | 0.08  0.29  0.22  0.72 | 0.92  0.71  0.78  0.28 | 0.04  0.18  0.41  0.36 | 0.27  0.45 | 0.69  0.82  0.14  0.64 | 0.18  0.18  0.64  0.36 | 0.82  0.82  0.36  0.64 | 0.18  -  0.35  - | 0.82  -  0.65  - |
| 2019 | Flück^73^ | *ACE* I/D  (rs1799752)  rs1815739  rs2104772 | II  TT  AA | *ACE*  *ACTN3*  *TNC* | II ID DD - I D  TT CT CC / T C  AA AT TT / A T | 0.28  0.30  0.20 | 0.48  0.48  0.65 | 0.24  0.22  0.15 | 0.52  0.54  0.53 | 0.48  0.46  0.47 | 0.14  0.35  0.19 | 0.47  0.39  0.64 | 0.39  0.26  0.17 | 0.38  0.55  0.51 | 0.62  0.45  0.49 | -  0.43  0.44 | -  0.57  0.56 |
| 2020 | Ben-Zaken^84^ | rs1800795  rs2854744 | CC  AA | *IL6*  *IGFBP3* | CC CG GG / C G  AA AC CC / A C | 0.03  0.30 | 0.22  0.51 | 0.75  0.19 | 0.14  0.55 | 0.86  0.45 | 0.03  0.20 | 0.34  0.56 | 0.63  0.24 | 0.20  0.48 | 0.80  0.52 | 0.36  0.30 | 0.64  0.70 |
| 2020 | Delgado^28^ | rs17602729  rs8192678  rs1799945  rs1800562 | CC  GG  GG+CG  GG | *AMPD1*  *PPARGC1A*  *HFE*  *HFE* | TT CT CC / T C  AA AG GG / A G  GG CG CC / G C  AA AG GG/ A/G | 0  0  0.07  0 | 0.20  0.37  0.55  0.07 | 0.80  0.63  0.38  0.93 | 0.10  0.19  0.35  0.04 | 0.90  0.81  0.65  0.96 | 0  0.07  0  0 | 0.34  0.40  0.28  0.07 | 0.66  0.53  0.72  0.93 | 0.17  0.27  0.50  0.04 | 0.83  0.73  0.50  0.96 | 0.12  0.33  0.14  0.01 | 0.88  0.67  0.86  0.99 |
| 2021 | Delgado^83^ | *ACE* I/D  (rs4340)  rs2070744  rs1799983  rs1800544  rs553668  rs1042713  rs1042714  *BDKRB2*  *-9/+9^†^* | II  TT  GG  CC  GG  GG^‡^  CC  +9/+9^‡^ | *ACE*  *NOS3*  *NOS3*  *ADRA2A*  *ADRA2A*  *ADRB2*  *ADRB2*  *BDKRB2* | II ID DD - I D  CC TC TT / C T  TT GT GG / T G  GG CG CC / G C  AA GA GG / A G  AA AG GG / A G  GG GC CC / G C  -9/-9 +9/-9 +9/+9 | 0.05  0.20  0.03  0.69  0.02  0.09  0.14  0.21 | 0.28  0.29  0.54  0.25  0.21  0.55  0.61  0.44 | 0.67  0.51  0.43  0.06  0.77  0.36  0.25  0.35 | 0.19  0.35  0.30  0.82  0.13  0.37  0.45  0.43 | 0.81  0.65  0.70  0.18  0.87  0.63  0.55  0.57 | 0.11  0.24  0.10  0.55  0.07  0.08  0.32  0.25 | 0.53  0.44  0.51  0.43  0.22  0.54  0.51  0.53 | 0.36  0.32  0.39  0.02  0.71  0.38  0.17  0.22 | 0.38  0.46  0.36  0.77  0.18  0.35  0.58  0.52 | 0.62  0.54  0.64  0.23  0.82  0.65  0.42  0.48 | -  0.35  0.31  0.33  0.17  0.39  0.40  - | -  0.65  0.69  0.67  0.83  0.61  0.60  - |

Estimated world population allele frequencies retrieved from ALFA: [www.ncbi.nlm.nih.gov/snp/docs/gsr/alfa/](http://www.ncbi.nlm.nih.gov/snp/docs/gsr/alfa/) (last accessed on 26^th^ of April 2022).

For indels, no global allele frequency was reported.

NR=genotype frequency not reported in original article.

**†** Rs-number not mentioned in original article.

‡ Original article reports the opposite “effect allele” as favorable for endurance performance.

# **Supplementary 9.** Risk of bias assessment of the 43 articles included in the systematic review.


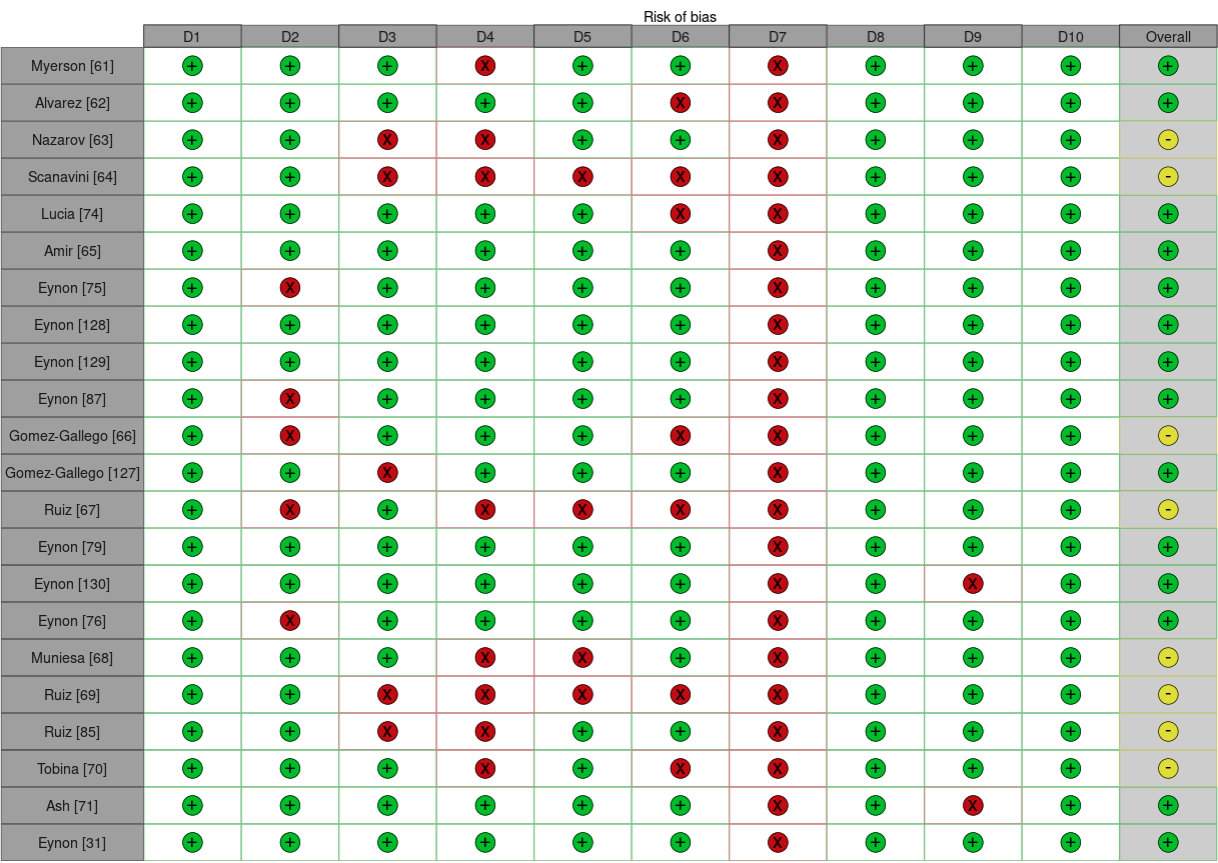


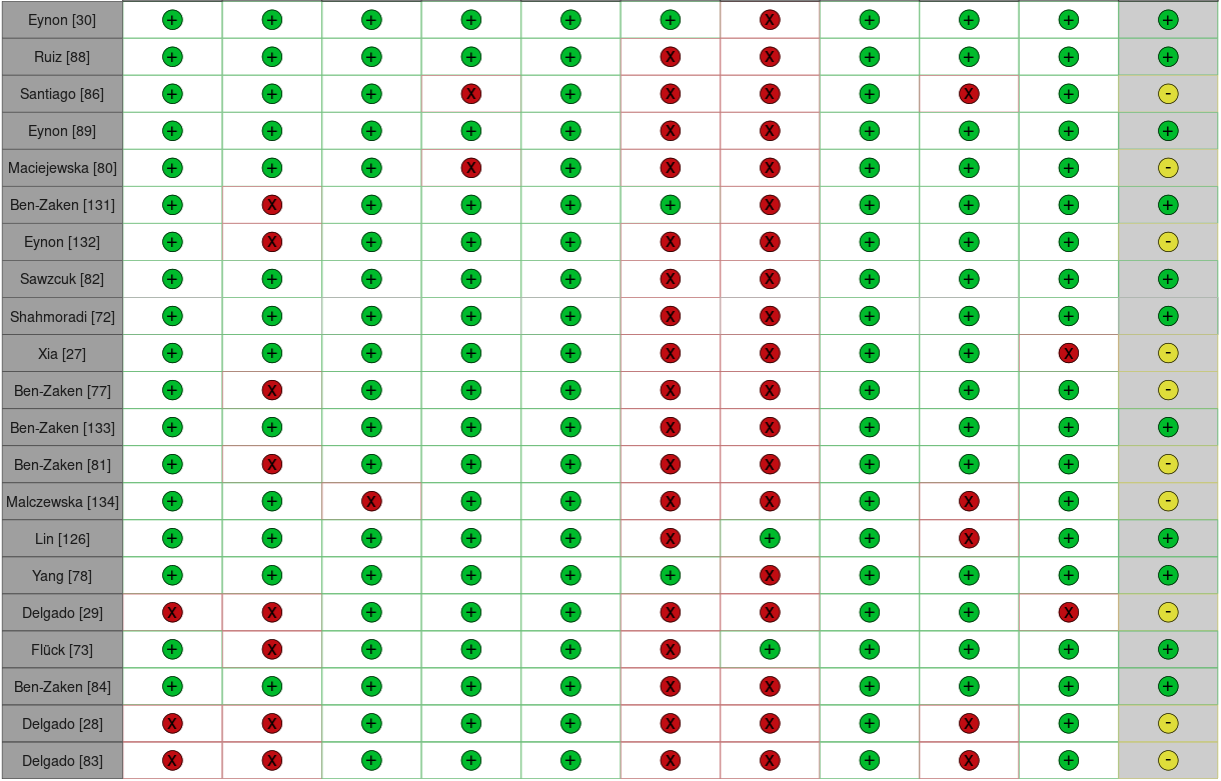


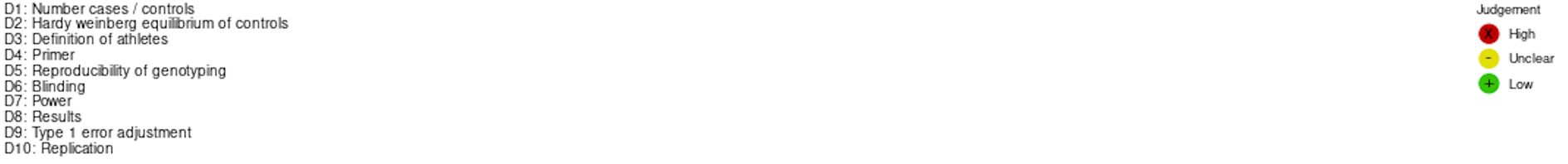


# **Supplementary 10**. Unique polymorphisms without statistical pooling (n=29).

| Year | First author | Rs-number/ marker  (Reported in article) | Genotypes | Gene  (Reported in article) | N athletes, cases/total  N controls,  cases/total | Odds ratio  [95% confidence interval] | Sport  discipline | Performance level | Sex | Country  of study population | Risk of bias |
| --- | --- | --- | --- | --- | --- | --- | --- | --- | --- | --- | --- |
| 2009 | Eynon^128^ | rs2016520 | TT | *PPARD* | 29/74  93/240 | 1.02  [0.60; 1.74] | Running | National, International | Male,  Female | Israel | 9 |
| 2009 | Eynon^129^ | **rs7181866** | **GG+AG^†^** | ***GABPB1***  ***(NRF2)*** | **9/74**  **5/240** | **6.51**  **[2.1; 20.09]** | Running | National, International | Male,  Female | Israel | 9 |
| 2009 | Gomez-Gallego^127^ | rs699 | CC | *AGT* | 16/100  19/119 | 1.00  [0.49; 2.07] | Running,  Cycling | International | Male | Spain | 8 |
| 2010 | Eynon^79^ | **rs4253778** | **GG** | ***PPARA*** | **7/74**  **10/240** | **2.40**  **[0.88; 6.56]** | Running | National, International | Male,  Female | Israel | 9 |
| 2010 | Eynon^130^ | **rs12594956**  **rs8031031** | **AA**  **T^†^** | ***GABPB1***  ***(NRF2)***  ***GABPB1***  ***(NRF2)*** | **43/74**  **102/240**  **8/74**  **9/240** | **1.88**  **[1.11; 3.18]**  **3.11**  **[1.56; 8.38]** | Running | National, International | Male,  Female | Israel | 8 |
| 2010 | Eynon^76^ | rs11549465 | T**^†^** | *HIF1A* | 21/74  67/240 | 1.02  [0.57; 1.82] | Running | National, International | Male,  Female | Israel | 8 |
| 2011 | Ash^71^ | rs4363 | AA | *ACE* | 10/76  50/410 | 1.09  [0.53; 2.26] | Running | International | Male,  Female | Ethiopia | 8 |
| 2011 | Santiago^86^ | rs1801253  **rs4994** | CC  **CC+CT^†^** | *ADRB1*  ***ADRB3*** | 48/100  43/100  **27/100**  **8/100** | 1.22  [0.70; 2.14]  **4.25**  **[1.8; 9.92]** | Running,  Cycling | International | Male | Spain | 6 |
| 2013 | Ben-Zaken^131^ | **rs4880**  **(rs1799725)** | **CC** | ***SOD2***  ***(MnSOD)*** | **29/121**  **33/240** | **1.98**  **[1.13; 3.45]** | Running | National, International | Male,  Female | Israel | 8 |
| 2013 | Eynon^132^ | rs9939609 | AA | *FTO* | 5/49  5/60 | 1.25  [0.34; 4.59] | Running | International | Male | Spain | 7 |
| 2014 | Xia^27^ | **rs5418** | **AA** | ***SLC2A4*** | **63/102**  **94/206** | **1.92**  **[1.19; 3.12]** | Running | International | Male,  Female | China | 7 |
| 2015 | Ben-Zaken^133^ | rs1049434 | AA | *SLC16A1*  *(MCT1)* | 33/61  56/128 | 1.52  [0.82; 2.80] | Running | National, International | Male,  Female | Israel | 8 |
| 2016 | Malczewska^134^ | Intron 2, +16 C/G^†^  -551 C/T^†^ | CC  CC | *HBB*  *HBB* | 6/89  3/109  18/89  19/109 | 2.55  [0.62;10.52]  1.20  [0.59; 2.46] | Cycling | National | Male,  Female | Poland | 6 |
| 2017 | Lin^126^ | **rs1472955**  rs4672568  **rs11904281** | **AA**  AA+AG**^†^**  **GG** | ***MYL1***  *MYL1*  ***MYL1*** | **15/31**  **62/206**  23/31  148/206  **16/31**  **68/206** | **2.18**  **[1.01; 4.68]**  1.13  [0.48; 2.66]  **2.16**  **[1.01; 4.64]** | Running | National, International | Male,  Female | China | 8 |
| 2019 | Delgado^29^ | **rs3892097**  Functional(+)/  Null(-)  **rs1695**  **Functional(+)/**  **Null(-)** | **GG**  -  **AA**  **+** | ***CYP2D6***  *GSTM1*  ***GSTP1***  ***(GSTP)***  ***GSTT*** | **105/123**  **76/122**  87/123  77/122  **77/123**  **61/122**  **89/123**  **70/122** | **3.53**  **[1.9; 6.56]**  1.41  [0.83; 2.41]  **1.67**  **[1.01; 2.79]**  **1.94**  **[1.14; 3.32]** | Running,  Cycling | International | Male | Spain | 6 |
| 2019 | Flück^73^ | rs2104772 | AA | *TNC* | 6/30  12/63 | 1.06  [0.36; 3.17] | Running,  Cycling | International | Male,  Female | Switzer  land | 8 |
| 2020 | Ben-Zaken^84^ | rs2854744 | AA | *IGFBP3* | 19/63  13/64 | 1.69  [0.75; 3.82] | Running | National, International | Male,  Female | Israel | 8 |
| 2020 | Delgado^28^ | rs1800562 | GG | *HFE* | 114/123  113/122 | 1.01  [0.39; 2.63] | Running,  Cycling | International | Male | Spain | 5 |
| 2021 | Delgado^83^ | rs1799983  rs1800544  rs553668 | GG  CC  GG | *NOS3*  *ADRA2A*  *ADRA2A* | 53/123  47/122  7/123  3/122  94/123  87/122 | 1.21  [0.73; 2.01]  2.39  [0.60; 9.48]  1.30  [0.74; 2.31] | Running,  Cycling | International | Male | Spain | 5 |

**†** a dominant inheritance model was assumed.

Significant results are displayed in bold.

# **Supplementary 11.** Characteristics of studies analyzing *ACE* I/D sorted by Odds ratio.

| Year | First author | Rs-number/ marker | Genotype | Gene | N athletes, cases/total  N controls,  cases/total | Odds ratio  [95% confidence interval] | Sport  discipline | Performance level | Sex | Country  of study population | Risk of bias |
| --- | --- | --- | --- | --- | --- | --- | --- | --- | --- | --- | --- |
| 2021 | Delgado^83^ | *ACE* I/D | II | *ACE* | 41/123  78/122 | 0.43  [0.16; 1.17] | Running,  Cycling | International | Male | Spain | 6 |
| 2001 | Nazarov^63^ | *ACE* I/D | II | *ACE* | 2/10  105/449 | 0.82  [0.17; 3.92] | Running | National | Male,  Female | Russia | 7 |
| 2007 | Amir^65^ | *ACE* I/D | II | *ACE* | 7/79  26/247 | 0.83  [0.34; 1.98] | Running | National | Male,  Female | Israel | 9 |
| 2014 | Shahmoradi^72^ | *ACE* I/D | II | *ACE* | 6/37  27/163 | 0.97  [0.37; 2.56] | Cycling | National,  International | Male | Iran | 8 |
| 2010 | Ruiz^69^ | *ACE* I/D | II | *ACE* | 44/100  44/100 | 1.0  [0.57; 1.75] | Running,  Cycling | National,  International | Male | Spain | 5 |
| 2010 | Tobina^70^ | *ACE* I/D | II | *ACE* | 19/37  155/335 | 1.23  [0.62; 2.42] | Running | National,  International | Male | Japan | 7 |
| 2009 | Gomez-Gallego^66^ | *ACE* I/D | II | *ACE* | 13/46  11/46 | 1.25  [0.49; 3.19] | Cycling | International | Male | Spain | 7 |
| 2010 | Muniesa^68^ | *ACE* I/D | II | *ACE* | 31/102  24/123 | 1.80  [0.97; 3.33] | Running,  Cycling | International | Male | Spain | 7 |
| 2009 | Ruiz^67^ | *ACE* I/D | II | *ACE* | 14/46  23/123 | 1.90  [0.88; 4.13] | Running,  Cycling | National,  International | Male | Spain | 6 |
| 2011 | Ash^71^ | *ACE* I/D | II | *ACE* | 12/76  36/408 | 1.94  [0.96; 3.92] | Running | International | Male,  Female | Ethiopia | 8 |
| 2000 | Alvarez^62^ | *ACE* I/D | II | *ACE* | 12/45  62/400 | 1.98  [0.97; 4.05] | Running,  Cycling | National,  International | Male | Spain | 8 |
| 2019 | Flück^73^ | *ACE* I/D | II | *ACE* | 8/30  9/63 | 2.18  [0.75; 6.38] | Running,  Cycling | International | Male,  Female | Switzerland | 9 |
| 1999 | **Myerson^61^** | ***ACE* I/D** | **II** | ***ACE*** | **14/34**  **457/1906** | **2.22**  **[1.11; 4.43]** | **Running** | **International** | **Male,**  **Female** | **Britain** | **8** |
| 2002 | Scanavini^64^ | *ACE* I/D | II | *ACE* | 5/17  19/152 | 2.92  [0.92; 9.20] | Running,  Cycling | International | Male,  Female | Italy | 5 |

Significant results are shown in bold.

Risk of Bias assessment might deviate from general risk of bias assessment (online supplement F) due to Hardy Weinberg test of controls.

# **Supplementary 12.** Risk of bias assessment of *ACE* I/D sorted by odds ratio; assessment might deviate from general risk of bias assessment (ESM 9) due to Hardy Weinberg test of controls.


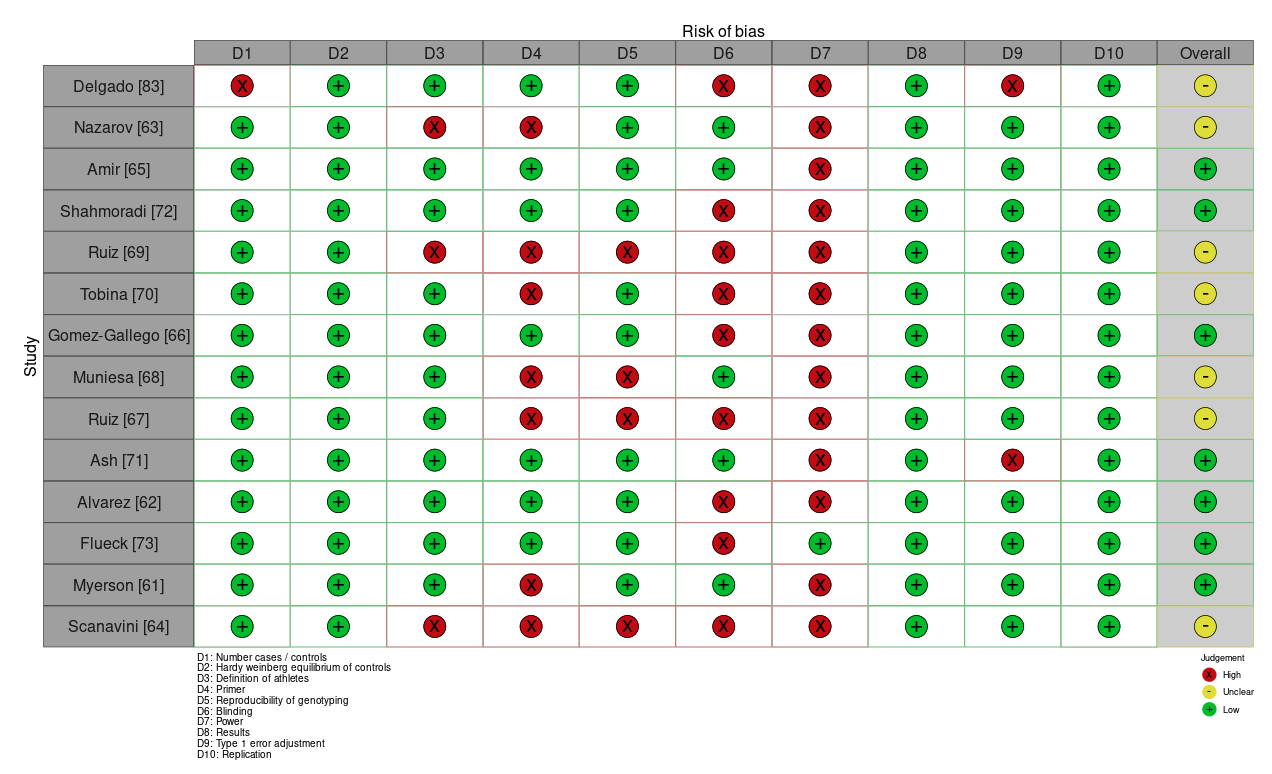


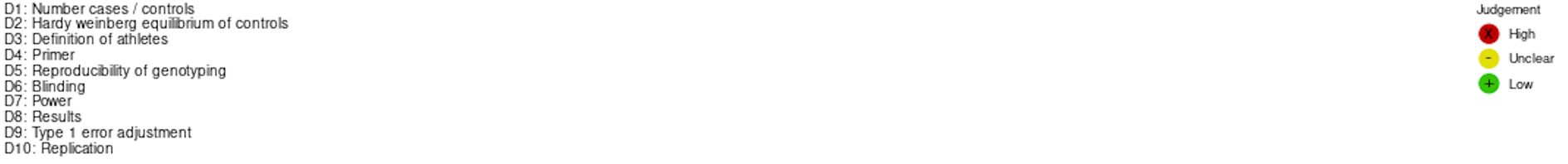


# **Supplementary 13.** Funnel plot *ACE* I/D.


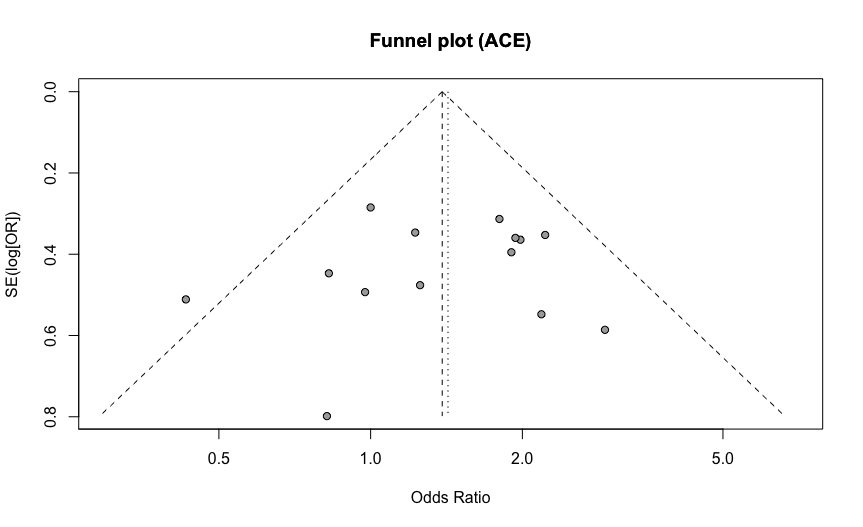


# **Supplementary 14.** Characteristics of studies analyzing *ACTN3* (rs1815739) sorted by Odds ratio.

| Year | First author | Rs-number/ marker | Genotype | Gene | N athletes, cases/total  N controls,  cases/total | Odds ratio  [95% confidence interval] | Sport  discipline | Performance level | Sex | Country  of study population | Risk of bias |
| --- | --- | --- | --- | --- | --- | --- | --- | --- | --- | --- | --- |
| 2019 | Flück^73^ | rs1815739 | TT | *ACTN3* | 10/30  25/63 | 0.76  [0.31; 1.89] | Running,  Cycling | International | Male,  Female | Switzerland | 8 |
| 2017 | Yang^78^ | rs1815739 | TT | *ACTN3* | 14/44  17/50 | 0.91  [0.38; 2.15] | Running | National,  International | Male,  Female | China | 9 |
| 2010 | Muniesa^68^ | rs1815739 | TT | *ACTN3* | 22/102  22/123 | 1.26  [0.65; 2.44] | Running,  Cycling | International | Male | Spain | 7 |
| 2009 | Ruiz^67^ | rs1815739 | TT | *ACTN3* | 11/46  22/123 | 1.44  [0.64; 3.27] | Running,  Cycling | National,  International | Male | Spain | 6 |
| 2009 | Gomez-Gallego^66^ | rs1815739 | TT | *ACTN3* | 11/46  8/46 | 1.49  [0.54; 4.14] | Cycling | International | Male | Spain | 7 |
| 2006 | Lucia^74^ | rs1815739 | TT | *ACTN3* | 14/50  22/123 | 1.79  [0.83; 3.86] | Cycling | National | Male | Spain | 8 |
| 2009 | **Eynon^75^** | **rs1815739** | TT | ***ACTN3*** | **24/74**  **42/240** | **2.26**  **[1.25; 4.08]** | **Running** | **National,**  **International** | **Male,**  **Female** | **Israel** | **8** |
| 2015 | **Ben-Zaken^77^** | **rs1815739** | TT | ***ACTN3*** | **23/65**  **40/217** | **2.42**  **[1.31; 4.47]** | **Running** | **National,**  **International** | **Male,**  **Female** | **Israel** | **7** |
| 2010 | **Ruiz^69^** | **rs1815739** | TT | ***ACTN3*** | **29/100**  **13/100** | **2.73**  **[1.32; 5.65]** | **Running,**  **Cycling** | **National,**  **International** | **Male** | **Spain** | **5** |

Significant results are shown in bold.

Risk of Bias assessment might deviate from general risk of bias assessment (ESM 9) due to Hardy Weinberg test of controls.

# **Supplementary 15.** Risk of bias assessment of *ACTN3* (rs1815739) sorted by odds ratio; assessment might deviate from general risk of bias assessment (ESM 9) due to Hardy Weinberg test of controls.

*
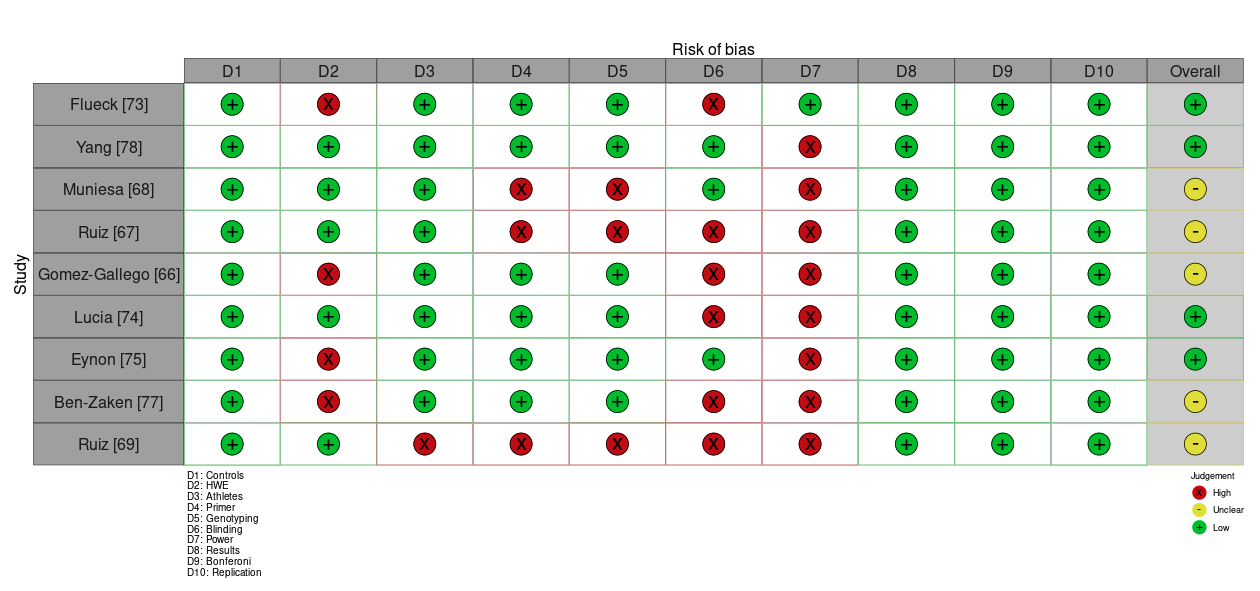
*


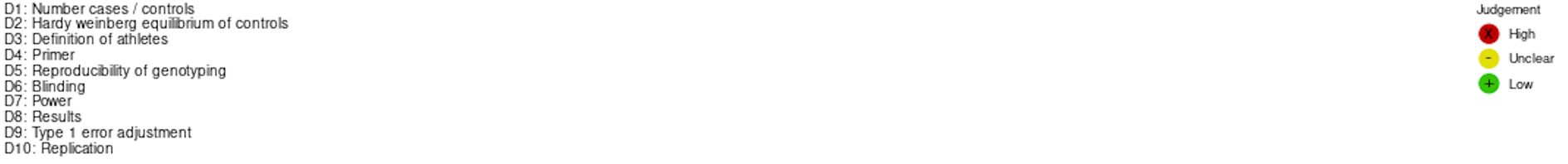


# **Supplementary 16.** Funnel plot *ACTN3* (rs1815739).

*
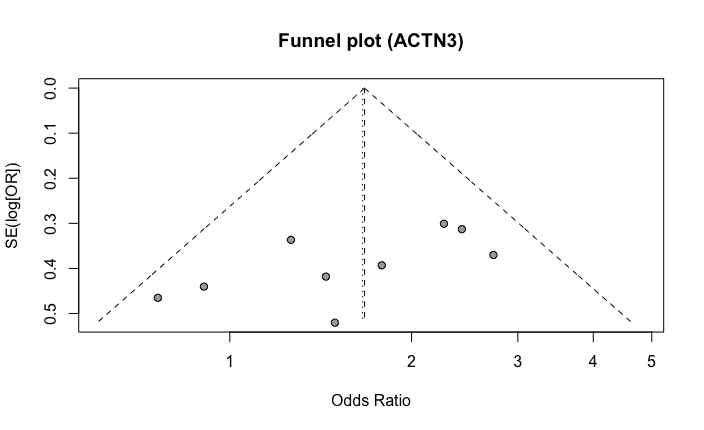
*

# **Supplementary 17.** Characteristics of studies analyzing *PPARGC1A* (rs8192678) sorted by Odds ratio.

| Year | First author | Rs-number/ marker | Genotype | Gene | N athletes, cases/total  N controls,  cases/total | Odds ratio  [95% confidence interval] | Sport  discipline | Performance level | Sex | Country  of study population | Risk of bias |
| --- | --- | --- | --- | --- | --- | --- | --- | --- | --- | --- | --- |
| 2020 | Delgado^28^ | rs8192678 | GG | *PPARGC1A* | 77/123  65/122 | 1.47  [0.88; 2.44] | Running,  Cycling | International | Male | Spain | 6 |
| 2010 | Muniesa^68^ | rs8192678 | GG | *PPARGC1A* | 49/102  47/123 | 1.49  [0.88; 2.55] | Running,  Cycling | International | Male | Spain | 7 |
| 2010 | **Eynon^79^** | **rs8192678** | **GG** | ***PPARGC1A*** | **37/74**  **79/240** | **2.04**  **[1.2; 3.46]** | **Running** | **National,**  **International** | **Male,**  **Female** | **Israel** | **9** |
| 2009 | **Ruiz^67^** | **rs8192678** | **GG** | ***PPARGC1A*** | **26/46**  **47/123** | **2.10**  **[1.06; 4.18]** | **Running,**  **Cycling** | **National,**  **International** | **Male** | **Spain** | **6** |
| 2012 | Maciejewska^80^ | rs8192678 | GG | *PPARGC1A* | 9/14  280/684 | 2.60  [0.86; 7.83] | Cycling | International | Male,  Female | Poland  Russia | 7 |

Significant results are shown in bold.

Risk of Bias assessment might deviate from general risk of bias assessment (ESM 9) due to Hardy Weinberg test of controls.

# **Supplementary 18.** Risk of bias assessment of *PPARGC1A* (rs8192678) sorted by odds ratio; assessment might deviate from general risk of bias assessment (ESM 9) due to Hardy Weinberg test of controls.


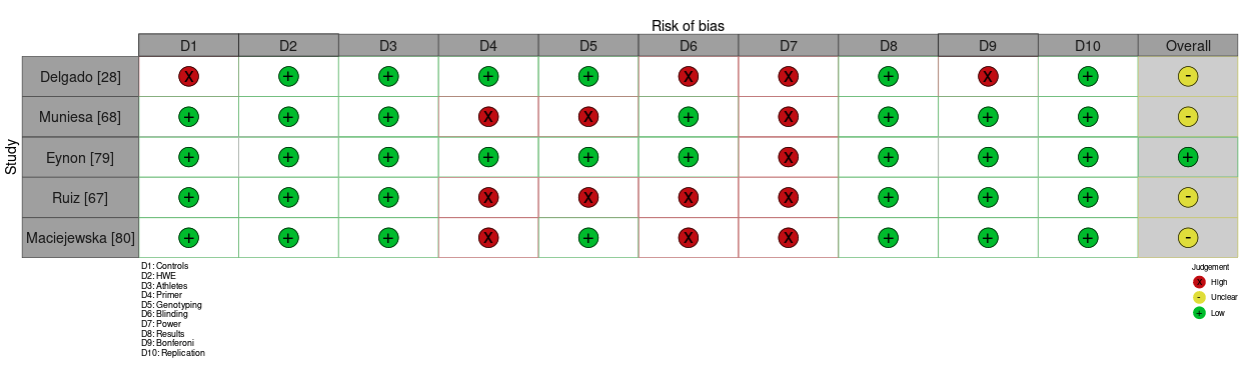


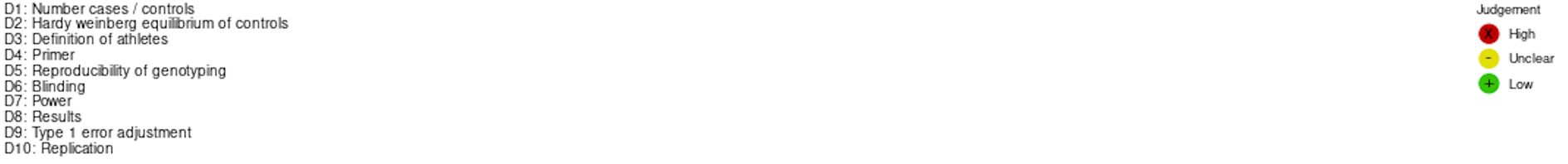


# **Supplementary 19.** Funnel plot *PPARGC1A* (rs8192678).

*
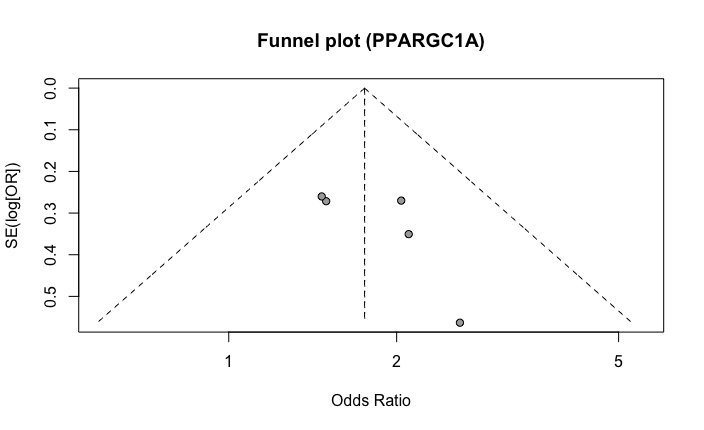
*

# **Supplementary 20.** Characteristics of studies analyzing *AMPD1* (rs17602729) sorted by Odds ratio.

| Year | First author | Rs-number/ marker | Genotype | Gene | N athletes, cases/total  N controls,  cases/total | Odds ratio  [95% confidence interval] | Sport  discipline | Performance level | Sex | Country  of study population | Risk of bias |
| --- | --- | --- | --- | --- | --- | --- | --- | --- | --- | --- | --- |
| 2020 | **Delgado^28^** | **rs17602729** | **CC** | **AMPD1** | **98/123**  **81/122** | **1.98**  **[1.11; 3.54]** | **Running,**  **Cycling** | **International** | **Male** | **Spain** | **5** |
| 2010 | Muniesa^68^ | rs17602729 | CC | AMPD1 | 93/102  101/123 | 2.25  [0.99; 5.14] | Running,  Cycling | International | Male | Spain | 7 |
| 2009 | **Ruiz^67^** | **rs17602729** | **CC** | **AMPD1** | **44/46**  **101/123** | **4.79**  **[1.08; 21.27]** | **Running,**  **Cycling** | **National,**  **International** | **Male** | **Spain** | **6** |

Significant results are shown in bold.

Risk of Bias assessment might deviate from general risk of bias assessment (ESM 9) due to Hardy Weinberg test of controls.

# **Supplementary 21.** Risk of bias assessment *AMPD1* (rs17602729) sorted by odds ratio; assessment might deviate from general risk of bias assessment (ESM 9) due to Hardy Weinberg test of controls.


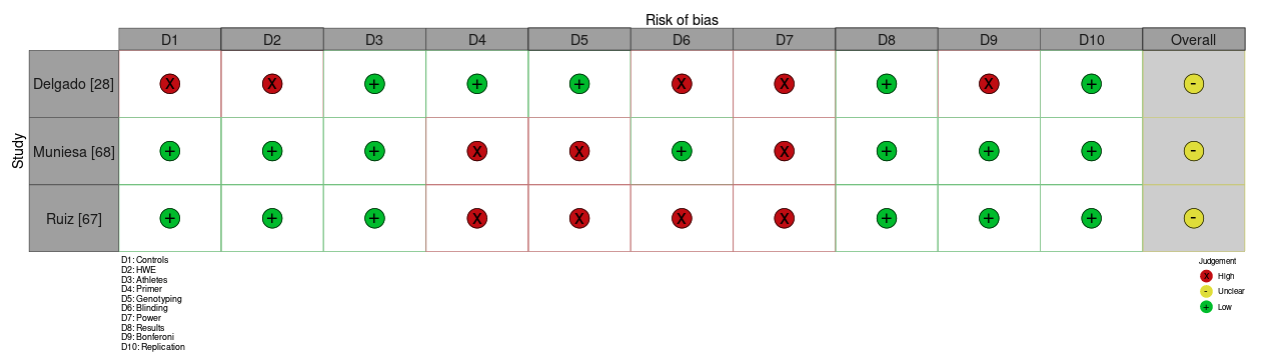


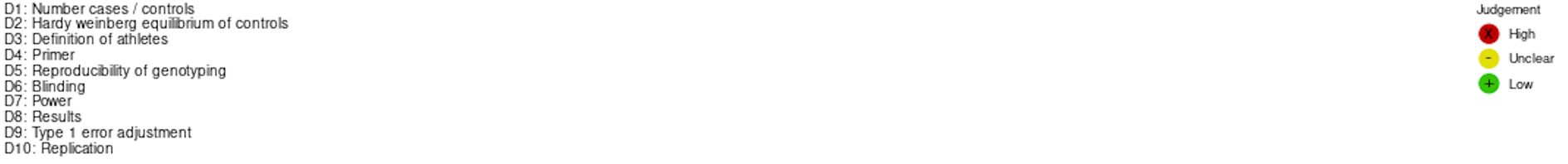


# **Supplementary 22.** Characteristics of studies analyzing *HFE* (rs1799945) sorted by Odds ratio, a dominant inheritance model was assumed.

| Year | First author | Rs-number/ marker | Genotype | Gene | N athletes, cases/total  N controls,  cases/total | Odds ratio  [95% confidence interval] | Sport  discipline | Performance level | Sex | Country  of study population | Risk of bias |
| --- | --- | --- | --- | --- | --- | --- | --- | --- | --- | --- | --- |
| 2009 | Ruiz^67^ | rs1799945 | GG+CG | *HFE* | 22/46  41/123 | 1.83  [0.92; 3.65] | Running,  Cycling | National,  International | Male | Spain | 5 |
| 2020 | **Delgado^28^** | **rs1799945** | **GG+CG** | ***HFE*** | **74/123**  **34/122** | **4.19**  **[2.45; 7.16]** | **Running,**  **Cycling** | **International** | **Male** | **Spain** | **6** |

Significant results are shown in bold.

Risk of Bias assessment might deviate from general risk of bias assessment due to Hardy Weinberg test of controls.

# **Supplementary 23.** Risk of bias assessment HFE (rs1799945) sorted by odds ratio; assessment might deviate from general risk of bias assessment (ESM 9) due to Hardy Weinberg test of controls.


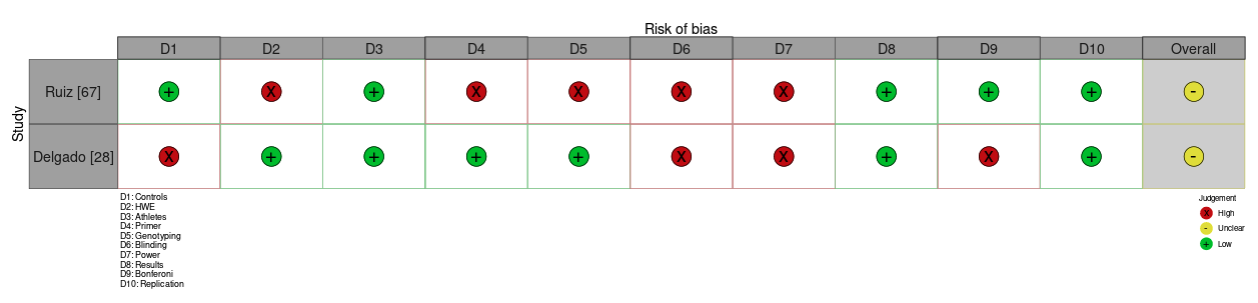


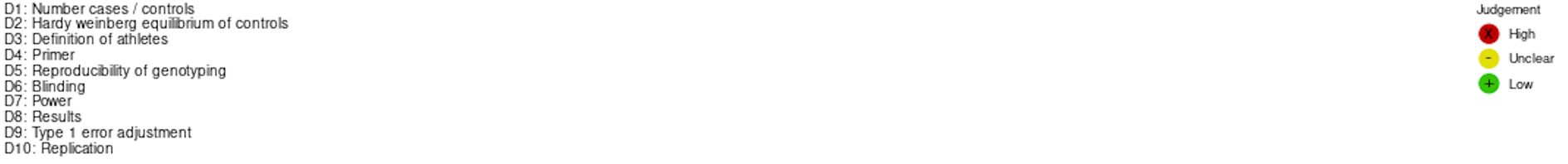


# **Supplementary 24.** Sensitivity analysis.

| Rs-number/ marker | Genotypes | Gene  (Reported in article) | N of included studies | Main model | Sensitivity analysis 1 | Sensitivity analysis 2 | Sensitivity analysis 3 | Sensitivity analysis 4 |
| --- | --- | --- | --- | --- | --- | --- | --- | --- |
| *ACE* I/D | **II** | ***ACE*** | **14** | **1.42**  **[1.12-1.81]** | **1.39**  **[1.12-1.71]** | 0.82  [0.57-1.17] | **1.42**  **[1.09-1.86]** | NA |
| rs1815739 | **TT** | ***ACTN3*** | **9** | **1.66**  **[1.26-2.19]** | **1.67**  **[1.31-2.14]** | 1.14  [0.89-1.46] | **1.66**  **[1.20-2.30]** | **1.55**  **[1.09-2.21]** |
| rs8192678 | **GG** | ***PPARGC1A*** | **5** | **1.75**  **[1.34-2.29]** | **1.75**  **[1.34-2.29]** | 4.11  [0.69 -24.47] | **1.75**  **[1.35-2.27]** | NA |
| rs17602729 | **CC** | ***AMPD1*** | **3** | **2.23**  **[1.42-3.51]** | **2.31**  **[1.48-3.61]** | 1.69  [0.17-16.43] | **2.23**  **[1.05-4.76]** | **2.69**  **[1.31-5.53]** |
| rs1799945 | **GG+CG** | ***HFE*** | **2** | NA | NA | **2.85**  **[1.27-6.39]** | 2.85  [0.02-533.22] | - |
| rs1805086 | GG+AG | *MSTN*  *(GDF8)* | 4 | NA | NA | 1.40  [0.90-2.19] | **1.40**  **[1.11-1.78]** | 1.29  [0.75-2.22] |
| *BDKRB* -9/+9 | +9/+9 | *BDKRB2* | 3 | 1.44  [0.98-2.11] | 1.44  [0.99-2.10] | 1.22  [0.79-1.88] | 1.44  [0.66-3.13] | NA |
| rs1800795 | CC | *IL6* | 3 | 1.23  [0.62-2.44] | 1.23  [0.62-2.43] | 0.88  [0.62-1.26] | 1.23  [0.93-1.61] | NA |
| rs1042713 | GG | *ADRB2* | 2 | 0.99  [0.67-1.46] | 0.99  [0.67-1.46] | 1.12  [0.66-1.89] | 0.99  [0.34-2.92] | - |
| rs1042714 | CC | *ADRB2* | 2 | 1.29  [0.86-1.96] | 1.30  [0.86-1.96] | 1.68  [0.54-5.22] | 1.29  [0.10-16.45] | - |
| rs8111989 | TT | *CKMM* | 2 | 1.50  [0.98-2.30] | 1.50  [0.98-2.30] | 1.64  [0.79-3.38] | **1.50**  **[1.03-2.20]** | - |
| rs5443 | TT | *GNB3* | 2 | 1.32  [0.36-4.86] | 1.32  [0.36-4.86] | 1.36  [0.92-2.03] | 1.32  [0.00-6032.61] | - |
| rs2070744 | TT | *NOS3* | 2 | 1.46  [0.65-3.24] | **1.51**  **[1.03-2.22]** | 0.15  [0.03-0.82] | 1.46  [0.01-259.81] | - |

Main model: Recessive inheritance model assumed; random effects model used; NA=not applicable (i.e., zero counts were reported for the effect allele).

Sensitivity analysis 1: Recessive inheritance model assumed; fixed effect model used.

Sensitivity analysis 2: Dominant inheritance model assumed; random effects model used.

Sensitivity analysis 3: Hartung-Knapp adjustment for main model.

Sensitivity analysis 4: Excluding studies with control group not in Hardy Weinberg equilibrium (only applied when n of included studies >2).

Significant results are displayed in bold.

# **REFERENCES**

1. Tucker R, Collins M. What makes champions? A review of the relative contribution of genes and training to sporting success. *Br J Sports Med.* 2012;46(8):555-61.

2. Midgley AW, McNaughton LR, Jones AM. Training to enhance the physiological determinants of long-distance running performance: can valid recommendations be given to runners and coaches based on current scientific knowledge? *Sports Med.* 2007;37(10):857-80.

3. Bonetti DL, Hopkins WG. Sea-level exercise performance following adaptation to hypoxia: a meta-analysis. *Sports Med*. 2009;39(2):107-27.

4. Gould D, Dieffenbach K, Moffett A. Psychological Characteristics and Their Development in Olympic Champions. *J Appl Sport Psychol.* 2002;14(3):172-204.

5. Hawley JA. Nutritional strategies to modulate the adaptive response to endurance training. *Nestle Nutr Inst Workshop Ser.* 2013;75:1-14.

6. Moreland E, Borisov OV, Semenova EA, et al. Polygenic Profile of Elite Strength Athletes. *J Strength Cond Res.* 2020. doi.org/10.1519/jsc.0000000000003901

7. Ahmetov II, Williams AG, Popov DV, et al. The combined impact of metabolic gene polymorphisms on elite endurance athlete status and related phenotypes. *Hum Genet.* 2009;126(6):751-61.

8. Bouchard C, An P, Rice T, et al. Familial aggregation of VO(2max) response to exercise training: results from the HERITAGE Family Study. *J Appl Physiol (1985).* 1999;87(3):1003-8.

9. De Moor MH, Spector TD, Cherkas LF, et al. Genome-wide linkage scan for athlete status in 700 British female DZ twin pairs. *Twin Res Hum Genet.* 2007;10(6):812-20.

10. Ahmetov II, Egorova ES, Gabdrakhmanova LJ, et al. Genes and Athletic Performance: An Update. *Med Sport Sci.* 2016;61:41-54.

11. Puthucheary Z, Skipworth JR, Rawal J, et al. The ACE gene and human performance: 12 years on. *Sports Med.* 2011;41(6):433-48.

12. Ma F, Yang Y, Li X, et al. The association of sport performance with ACE and ACTN3 genetic polymorphisms: a systematic review and meta-analysis. *PLoS One.* 2013;8(1):e54685.

13. Degens H, Stasiulis A, Skurvydas A, et al. Physiological comparison between non-athletes, endurance, power and team athletes. *Eur J Appl Physiol.* 2019;119(6):1377-86.

14. Ahmetov II, Fedotovskaya ON. Current Progress in Sports Genomics. *Adv Clin Chem.* 2015;70:247-314.

15. Al-Khelaifi F, Diboun I, Donati F, et al. Metabolic GWAS of elite athletes reveals novel genetically-influenced metabolites associated with athletic performance. *Sci Rep.* 2019;9(1):19889.

16. Al-Khelaifi F, Yousri NA, Diboun I, et al. Genome-Wide Association Study Reveals a Novel Association Between MYBPC3 Gene Polymorphism, Endurance Athlete Status, Aerobic Capacity and Steroid Metabolism. *Front Genet.* 2020;11:595.

17. Boraita A, de la Rosa A, Heras ME, et al. Cardiovascular adaptation, functional capacity and Angiotensin-converting enzyme I/D polymorphism in elite athletes. *Rev Esp Cardiol.* 2010;63(7):810-9.

18. Mitchell JH, Haskell W, Snell P, et al. Task Force 8: classification of sports*. J Am Coll Cardiol.* 2005;45(8):1364-7.

19. Williams A, Day S, Lockey S, et al. Genomics as a practical tool in sport - have we reached the starting line? *Cell Mol Exerc Physiol.* 2014;3:e6.

20. Hall GV, Jensen-Urstad M, Rosdahl H, et al. Leg and arm lactate and substrate kinetics during exercise. *Am J Physiol Endocrinol Metab.* 2003;284(1):E193-E205.

21. Calbet JAL, Holmberg H-C, Rosdahl H, et al. Why do arms extract less oxygen than legs during exercise? *Am J Physiol Regul Integr Comp Physiol.* 2005;289(5):R1448-R58.

22. De Pauw K, Roelands B, Cheung SS, et al. Guidelines to classify subject groups in sport-science research. *Int J Sports Physiol Perform.* 2013;8(2):111-22.

23. Swann C, Moran A, Piggott D. Defining elite athletes: Issues in the study of expert performance in sport psychology. *Psychol Sport Exerc.* 2015;16:3-14.

24. Araújo CGS, Scharhag J. Athlete: a working definition for medical and health sciences research. *Scand J Med Sci Sports.* 2016;26(1):4-7.

25. Greco T, Zangrillo A, Biondi-Zoccai G, et al. Meta-analysis: pitfalls and hints. *Heart Lung Vessel.* 2013;5(4):219-25.

26. Page MJ, McKenzie JE, Bossuyt PM, et al. The PRISMA 2020 statement: An updated guideline for reporting systematic reviews. *PLoS Med.* 2021;18(3):e1003583.

27. Xia X, Hu Y, Xu L, et al. A functional promoter polymorphism of SLC2A4 is associated with aerobic endurance in a Chinese population. *Eur J Sport Sci.* 2014;14(1):53-9.

28. Varillas Delgado D, Tellería Orriols JJ, Monge Martín D, et al. Genotype scores in energy and iron-metabolising genes are higher in elite endurance athletes than in nonathlete controls. *Appl Physiol Nutr Metab.* 2020;45(11):1225-31.

29. Varillas Delgado D, Tellería Orriols JJ, Martín Saborido C. Liver-Metabolizing Genes and Their Relationship to the Performance of Elite Spanish Male Endurance Athletes; a Prospective Transversal Study. *Sports Med Open.* 2019;5(1):50.

30. Eynon N, Meckel Y, Alves AJ, et al. Is there an interaction between BDKRB2 -9/+9 and GNB3 C825T polymorphisms and elite athletic performance? *Scand J Med Sci Sports.* 2011;21(6):e242-6.

31. Eynon N, Ruiz JR, Meckel Y, et al. Is the -174 C/G polymorphism of the IL6 gene associated with elite power performance? A replication study with two different Caucasian cohorts. *Exp Physiol.* 2011;96(2):156-62.

32. Ben-Zaken S, Meckel Y, Nemet D, et al. Increased Prevalence of the IL-6-174C Genetic Polymorphism in Long Distance Swimmers. *J Hum Kinet.* 2017;58:121-30.

33. Jeukendrup AE, Craig NP, Hawley JA. The bioenergetics of World Class Cycling. *J Sci Med Sport*. 2000;3(4):414-33.

34. Lorenz DS, Reiman MP, Lehecka BJ, et al. What performance characteristics determine elite versus nonelite athletes in the same sport? *Sports Health.* 2013;5(6):542-7.

35. Thornton A, Lee P. Publication bias in meta-analysis: its causes and consequences. *J Clin Epidemiol.* 2000;53(2):207-16.

36. Clark MF, Baudouin SV. A systematic review of the quality of genetic association studies in human sepsis. *Intensive Care Med.* 2006;32(11):1706-12.

37. McGuinness LA, Higgins JPT. Risk-of-bias VISualization (robvis): An R package and Shiny web app for visualizing risk-of-bias assessments. *Res Synth Methods*. 2021;12(1):55-61.

38. Borenstein M, Hedges LV, Higgins JP, et al. A basic introduction to fixed-effect and random-effects models for meta-analysis. *Res Synth Methods.* 2010;1(2):97-111.

39. Higgins JP, Thompson SG, Spiegelhalter DJ. A re-evaluation of random-effects meta-analysis. *J R Stat Soc Ser A Stat Soc.* 2009;172(1):137-59.

40. DerSimonian R, Laird N. Meta-analysis in clinical trials. *Control Clin Trials.* 1986;7(3):177-88.

41. Olkin I, Dahabreh IJ, Trikalinos TA. GOSH - a graphical display of study heterogeneity. *Res Synth Methods.* 2012;3(3):214-23.

42. Baujat B, Mahé C, Pignon JP, et al. A graphical method for exploring heterogeneity in meta-analyses: application to a meta-analysis of 65 trials. *Stat Med.* 2002;21(18):2641-52.

43. Sterne JAC, Sutton AJ, Ioannidis JPA, et al. Recommendations for examining and interpreting funnel plot asymmetry in meta-analyses of randomised controlled trials. *BMJ.* 2011;343:d4002.

44. Peters JL, Sutton AJ, Jones DR, et al. Comparison of two methods to detect publication bias in meta-analysis. *Jama.* 2006;295(6):676-80.

45. Higgins J, Thompson S. Controlling the risk of spurious findings from meta-regression. *Stat Med.* 2004;23:1663-82.

46. Jackson D, Law M, Rücker G, et al. The Hartung-Knapp modification for random-effects meta-analysis: A useful refinement but are there any residual concerns? *Stat Med.* 2017;36(25):3923-34.

47. Minelli C, Thompson JR, Abrams KR, et al. How should we use information about HWE in the meta-analyses of genetic association studies? *Int J Epidemiol.* 2007;37(1):136-46.

48. Schünemann HJ, Higgins JP, Vist GE, et al. Chapter 14: Completing ‘Summary of findings’ tables and grading the certainty of the evidence. In: Higgins JPT, Thomas J, Chandler J, et al (editors). Cochrane Handbook for Systematic Reviews of Interventions version 6.2 (updated February 2021). Cochrane, 2021. [www.training.cochrane.org/handbook](http://www.training.cochrane.org/handbook). Accessed 15 Nov 2021.

49. Duvallet A, Duvallet E, Lhuissier F, et al. Do mutations H63D and C282Y of the gene HFE influence the kinetics of iron metabolism in elite cyclist? *FASEB J.* 2016;30(S1):1287.3-.3.

50. Lifanov AD, Khadyeva MN, Rakhmatullina L, et al. Influence of the GPX1 gene polymorphism to aerobic capacity and efficiency of glutathione supplementation in athletes. *Ross Fiziol Zh Im I M Sechenova.* 2014;100(2):248-55.

51. Nursal AF, Yigit S, Rustemoglu H, et al. A case-control study investigating the effect of MTHFR C677T variant on performance of elite athletes. *Endocr Metab Immune Disord Drug Targets.* 2020. doi.org/10.2174/1568026620666201022144819

52. Lucia A, Martin MA, Esteve-Lanao J, et al. C34T mutation of the AMPD1 gene in an elite white runner. *BMJ Case Rep.* 2009;2009:bcr0720080535.

53. Eynon N, Birk R, Meckel Y, et al. Physiological variables and mitochondrial-related genotypes of an athlete who excels in both short and long-distance running. *Mitochondrion.* 2011;11(5):774-7.

54. Gonzalez-Freire M, Santiago C, Verde Z, et al. Unique among unique. Is it genetically determined? *Br J Sports Med.* 2009;43(4):307-9.

55. Pickering C, Kiely J. Can Genetic Testing Predict Talent? A Case Study of 5 Elite Athletes. *Int J Sports Physiol Perform.* 2020;16(3):429-34.

56. Eynon N, Ruiz JR, Meckel Y, et al. Mitochondrial biogenesis related endurance genotype score and sports performance in athletes. *Mitochondrion.* 2011;11(1):64-9.

57. Gómez-Gallego F, Ruiz JR, Buxens A, et al. The -786 T/C polymorphism of the NOS3 gene is associated with elite performance in power sports. *Eur J Appl Physiol.* 2009;107(5):565-9.

58. Wuyun G, Hu Y, He Z, et al. The Short Tandem Repeat of the DMT1 Gene as a Molecular Marker of Elite Long-Distance Runners. *Int J Genomics.* 2019;2019:7064703.

59. Scott RA, Fuku N, Onywera VO, et al. Mitochondrial haplogroups associated with elite Kenyan athlete status. *Med Sci Sports Exerc.* 2009;41(1):123-8.

60. He ZH, Hu Y, Li YC, et al. Are calcineurin genes associated with athletic status? A function, replication study. *Med Sci Sports Exerc.* 2011;43(8):1433-40.

61. Myerson S, Hemingway H, Budget R, et al. Human angiotensin I-converting enzyme gene and endurance performance. *J Appl Physiol (1985).* 1999;87(4):1313-6.

62. Alvarez R, Terrados N, Ortolano R, et al. Genetic variation in the renin-angiotensin system and athletic performance. *Eur J Appl Physiol.* 2000;82(1-2):117-20.

63. Nazarov IB, Woods DR, Montgomery HE, et al. The angiotensin converting enzyme I/D polymorphism in Russian athletes*. Eur J Hum Genet.* 2001;9(10):797-801.

64. Scanavini D, Bernardi F, Castoldi E, et al. Increased frequency of the homozygous II ACE genotype in Italian Olympic endurance athletes. *Eur J Hum Genet.* 2002;10(10):576-7.

65. Amir O, Amir R, Yamin C, et al. The ACE deletion allele is associated with Israeli elite endurance athletes. *Exp Physiol.* 2007;92(5):881-6.

66. Gómez-Gallego F, Santiago C, González-Freire M, et al. Endurance performance: genes or gene combinations? *Int J Sports Med.* 2009;30(1):66-72.

67. Ruiz JR, Gómez-Gallego F, Santiago C, et al. Is there an optimum endurance polygenic profile? *J Physiol.* 2009;587(Pt 7):1527-34.

68. Muniesa CA, González-Freire M, Santiago C, et al. World-class performance in lightweight rowing: is it genetically influenced? A comparison with cyclists, runners and non-athletes. *Br J Sports Med.* 2010;44(12):898-901.

69. Ruiz JR, Arteta D, Buxens A, et al. Can we identify a power-oriented polygenic profile? *J Appl Physiol (1985).* 2010;108(3):561-6.

70. Tobina T, Michishita R, Yamasawa F, et al. Association between the angiotensin I-converting enzyme gene insertion/deletion polymorphism and endurance running speed in Japanese runners. *J Physiol Sci.* 2010;60(5):325-30.

71. Ash GI, Scott RA, Deason M, et al. No association between ACE gene variation and endurance athlete status in Ethiopians. *Med Sci Sports Exerc.* 2011;43(4):590-7.

72. Shahmoradi S, Ahmadalipour A, Salehi M. Evaluation of ACE gene I/D polymorphism in Iranian elite athletes. *Adv Biomed Res.* 2014;3:207.

73. Flück M, Kramer M, Fitze DP, et al. Cellular Aspects of Muscle Specialization Demonstrate Genotype - Phenotype Interaction Effects in Athletes. *Front Physiol.* 2019;10:526.

74. Lucia A, Gómez-Gallego F, Santiago C, et al. ACTN3 genotype in professional endurance cyclists. *Int J Sports Med.* 2006;27(11):880-4.

75. Eynon N, Duarte JA, Oliveira J, et al. ACTN3 R577X polymorphism and Israeli top-level athletes. *Int J Sports Med.* 2009;30(9):695-8.

76. Eynon N, Alves AJ, Meckel Y, et al. Is the interaction between HIF1A P582S and ACTN3 R577X determinant for power/sprint performance? *Metabolism.* 2010;59(6):861-5.

77. Ben-Zaken S, Eliakim A, Nemet D, et al. ACTN3 Polymorphism: Comparison Between Elite Swimmers and Runners. *Sports Med Open.* 2015;1(1):13.

78. Yang R, Shen X, Wang Y, et al. ACTN3 R577X Gene Variant Is Associated With Muscle-Related Phenotypes in Elite Chinese Sprint/Power Athletes. *J Strength Cond Res.* 2017;31(4):1107-15.

79. Eynon N, Meckel Y, Sagiv M, et al. Do PPARGC1A and PPARalpha polymorphisms influence sprint or endurance phenotypes? *Scand J Med Sci Sports.* 2010;20(1):e145-50.

80. Maciejewska A, Sawczuk M, Cieszczyk P, et al. The PPARGC1A gene Gly482Ser in Polish and Russian athletes. *J Sports Sci.* 2012;30(1):101-13.

81. Ben-Zaken S, Meckel Y, Nemet D, et al. Frequency of the MSTN Lys(K)-153Arg(R) polymorphism among track & field athletes and swimmers. *Growth Horm IGF Res.* 2015;25(4):196-200.

82. Sawczuk M, Timshina YI, Astratenkova IV, et al. The -9/+9 polymorphism of the bradykinin receptor Beta 2 gene and athlete status: a study involving two European cohorts. *Hum Biol.* 2013;85(5):741-56.

83. Varillas-Delgado D, Tellería Orriols JJ, Del Coso J. Genetic Profile in Genes Associated with Cardiorespiratory Fitness in Elite Spanish Male Endurance Athletes. *Genes (Basel).* 2021;12(8):1230.

84. Ben-Zaken S, Meckel Y, Nemet D, et al. The combined frequencies of the IL-6 G-174C and IGFBP3 A-202C polymorphisms among swimmers and runners. *Growth Horm IGF Res.* 2020;51:17-21.

85. Ruiz JR, Buxens A, Artieda M, et al. The -174 G/C polymorphism of the IL6 gene is associated with elite power performance. *J Sci Med Sport.* 2010;13(5):549-53.

86. Santiago C, Ruiz JR, Buxens A, et al. Trp64Arg polymorphism in ADRB3 gene is associated with elite endurance performance. *Br J Sports Med.* 2011;45(2):147-9.

87. Eynon N, Oliveira J, Meckel Y, et al. The guanine nucleotide binding protein beta polypeptide 3 gene C825T polymorphism is associated with elite endurance athletes. *Exp Physiol.* 2009;94(3):344-9.

88. Ruiz JR, Eynon N, Meckel Y, et al. GNB3 C825T Polymorphism and elite athletic status: A replication study with two ethnic groups. *Int J Sports Med.* 2011;32(2):151-3.

89. Eynon N, Ruiz JR, Yvert T, et al. The C allele in NOS3 -786 T/C polymorphism is associated with elite soccer player's status. *Int J Sports Med.* 2012;33(7):521-4.

90. Lavoie JL, Sigmund CD. Minireview: Overview of the Renin-Angiotensin System-An Endocrine and Paracrine System. *Endocrinology.* 2003;144(6):2179-83.

91. Rigat B, Hubert C, Alhenc-Gelas F, et al. An insertion/deletion polymorphism in the angiotensin I-converting enzyme gene accounting for half the variance of serum enzyme levels. *J Clin Invest.* 1990;86(4):1343-6.

92. van Ginkel S, de Haan A, Woerdeman J, et al. Exercise intensity modulates capillary perfusion in correspondence with ACE I/D modulated serum angiotensin II levels. *Appl Transl Genom.* 2015;4:33-7.

93. Ipekoglu G, Bulbul A, Cakir HI. A meta-analysis on the association of ACE and PPARA gene variants and endurance athletic status. *J Sports Med Phys Fitness.* 2021. doi.org/10.23736/s0022-4707.21.12417-x

94. Houweling PJ, Papadimitriou ID, Seto JT, et al. Is evolutionary loss our gain? The role of ACTN3 p.Arg577Ter (R577X) genotype in athletic performance, ageing, and disease. *Hum Mutat.* 2018;39(12):1774-87.

95. North KN, Yang N, Wattanasirichaigoon D, et al. A common nonsense mutation results in α-actinin-3 deficiency in the general population. *Nat Genet.* 1999;21(4):353-4.

96. Tharabenjasin P, Pabalan N, Jarjanazi H. Association of the ACTN3 R577X (rs1815739) polymorphism with elite power sports: A meta-analysis. *PloS one.* 2019;14(5):e0217390-e.

97. Alfred T, Ben-Shlomo Y, Cooper R, et al. ACTN3 genotype, athletic status, and life course physical capability: meta-analysis of the published literature and findings from nine studies. *Hum Mutat.* 2011;32(9):1008-18.

98. Wilson JM, Loenneke JP, Jo E, et al. The effects of endurance, strength, and power training on muscle fiber type shifting. *J Strength Cond Res.* 2012;26(6):1724-9.

99. Vincent B, De Bock K, Ramaekers M, et al. ACTN3 (R577X) genotype is associated with fiber type distribution. *Physiol Genomics.* 2007;32(1):58-63.

100. Papadimitriou ID, Lockey SJ, Voisin S, et al. No association between ACTN3 R577X and ACE I/D polymorphisms and endurance running times in 698 Caucasian athletes. *BMC Genomics.* 2018;19(1):13.

101. Tharabenjasin P, Pabalan N, Jarjanazi H. Association of PPARGC1A Gly428Ser (rs8192678) polymorphism with potential for athletic ability and sports performance: A meta-analysis. *PLoS One.* 2019;14(1):e0200967.

102. Valle I, Alvarez-Barrientos A, Arza E, et al. PGC-1alpha regulates the mitochondrial antioxidant defense system in vascular endothelial cells. *Cardiovasc Res.* 2005;66(3):562-73.

103. Liang H, Ward WF. PGC-1alpha: a key regulator of energy metabolism. *Adv Physiol Educ.* 2006;30(4):145-51.

104. Ling C, Poulsen P, Carlsson E, et al. Multiple environmental and genetic factors influence skeletal muscle PGC-1alpha and PGC-1beta gene expression in twins. *J Clin Invest.* 2004;114(10):1518-26.

105. Lin J, Wu H, Tarr PT, et al. Transcriptional co-activator PGC-1 alpha drives the formation of slow-twitch muscle fibres. *Nature.* 2002;418(6899):797-801.

106. Chen Y, Wang D, Yan P, et al. Meta-analyses of the association between the PPARGC1A Gly482Ser polymorphism and athletic performance. *Biol Sport.* 2019;36(4):301-9.

107. Yvert T, Miyamoto-Mikami E, Tobina T, et al. PPARGC1A rs8192678 and NRF1 rs6949152 Polymorphisms Are Associated with Muscle Fiber Composition in Women. *Genes (Basel).* 2020;11(9):1012.

108. Moir HJ, Kemp R, Folkerts D, et al. Genes and Elite Marathon Running Performance: A Systematic Review. *J Sports Sci Med.* 2019;18(3):559-68.

109. Fedotovskaya ON, Danilova AA, Akhmetov II. Effect of AMPD1 Gene Polymorphism on Muscle Activity in Humans. *Bull Exp Biol Med.* 2013;154(4):489-91.

110. Morisaki T, Gross M, Morisaki H, et al. Molecular basis of AMP deaminase deficiency in skeletal muscle. *Proc Natl Acad Sci USA.* 1992;89(14):6457-61.

111. Burt MJ, George PM, Upton JD, et al. The significance of haemochromatosis gene mutations in the general population: implications for screening. *Gut.* 1998;43(6):830-6.

112. Abbaspour N, Hurrell R, Kelishadi R. Review on iron and its importance for human health. *J Res Med Sci.* 2014;19(2):164-74.

113. Hinton PS. Iron and the endurance athlete. *Appl Physiol Nutr Metab.* 2014;39(9):1012-8.

114. Buratti P, Gammella E, Rybinska I, et al. Recent Advances in Iron Metabolism: Relevance for Health, Exercise, and Performance. *Med Sci Sports Exerc.* 2015;47(8):1596-604.

115. Hurrell RF. Bioavailability of iron. *Eur J Clin Nutr.* 1997;51 Suppl 1:S4-8.

116. Semenova EA, Miyamoto-Mikami E, Akimov EB, et al. The association of HFE gene H63D polymorphism with endurance athlete status and aerobic capacity: novel findings and a meta-analysis. *Eur J Appl Physiol.* 2020;120(3):665-73.

117. Gejl KD, Hvid LG, Andersson EP, et al. Contractile Properties of MHC I and II Fibers From Highly Trained Arm and Leg Muscles of Cross-Country Skiers. *Front Physiol.* 2021;12(855).

118. Helge JW. Arm and leg substrate utilization and muscle adaptation after prolonged low-intensity training. *Acta Physiologica.* 2010;199(4):519-28.

119. Ørtenblad N, Nielsen J, Boushel R, et al. The Muscle Fiber Profiles, Mitochondrial Content, and Enzyme Activities of the Exceptionally Well-Trained Arm and Leg Muscles of Elite Cross-Country Skiers. *Front Physiol.* 2018;9(1031).

120. Zinner C, Morales-Alamo D, Ørtenblad N, et al. The Physiological Mechanisms of Performance Enhancement with Sprint Interval Training Differ between the Upper and Lower Extremities in Humans. *Front Physiol.* 2016;7(426).

121. Okbay A, Rietveld CA. On improving the credibility of candidate gene studies: A review of candidate gene studies published in Emotion. *Emotion.* 2015;15(4):531-7.

122. Pickering C, Suraci B, Semenova EA, et al. A Genome-Wide Association Study of Sprint Performance in Elite Youth Football Players. *J Strength Cond Res.* 2019;33(9):2344-51.

123. Rankinen T, Fuku N, Wolfarth B, et al. No Evidence of a Common DNA Variant Profile Specific to World Class Endurance Athletes. *PLoS One.* 2016;11(1):e0147330.

124. Ahmetov II, Kulemin NA, Popov DV, et al. Genome-wide association study identifies three novel genetic markers associated with elite endurance performance. *Biol Sport.* 2015;32(1):3-9.

125. Lightfoot JT, Roth SM, Hubal MJ. Systems Exercise Genetics Research Design Standards. *Med Sci Sports Exerc.* 2021;53(5):883-7.

126. Lin X, Wang D, Wen L, et al. Intron polymorphism in MYL1 gene is associated with individual cardiac trainability to endurance training in human myocardium. *J Sports Med Phys Fitness.* 2017;57(1-2):144-53.

127. Gomez-Gallego F, Santiago C, González-Freire M, et al. The C allele of the AGT Met235Thr polymorphism is associated with power sports performance. *Appl Physiol Nutr Metab.* 2009;34(6):1108-11.

128. Eynon N, Meckel Y, Alves AJ, et al. Is there an interaction between PPARD T294C and PPARGC1A Gly482Ser polymorphisms and human endurance performance? *Exp Physiol.* 2009;94(11):1147-52.

129. Eynon N, Sagiv M, Meckel Y, et al. NRF2 intron 3 A/G polymorphism is associated with endurance athletes' status. *J Appl Physiol (1985).* 2009;107(1):76-9.

130. Eynon N, Alves AJ, Sagiv M, et al. Interaction between SNPs in the NRF2 gene and elite endurance performance. *Physiol Genomics.* 2010;41(1):78-81.

131. Ben-Zaken S, Eliakim A, Nemet D, et al. Increased prevalence of MnSOD genetic polymorphism in endurance and power athletes. *Free Radic Res.* 2013;47(12):1002-8.

132. Eynon N, Nasibulina ES, Banting LK, et al. The FTO A/T polymorphism and elite athletic performance: a study involving three groups of European athletes. *PLoS One.* 2013;8(4):e60570.

133. Ben-Zaken S, Eliakim A, Nemet D, et al. Differences in MCT1 A1470T polymorphism prevalence between runners and swimmers. *Scand J Med Sci Sports.* 2015;25(3):365-71.

134. Malczewska-Lenczowska J, Orysiak J, Majorczyk E, et al. Total Hemoglobin Mass, Aerobic Capacity, and HBB Gene in Polish Road Cyclists. *J Strength Cond Res.* 2016;30(12):3512-9.
